# Supplementary material for: Translesion synthesis by AMV, HIV, and MMLVreverse transcriptases using RNA templates containing inosine, guanosine, and their 8-oxo-7,8-dihydropurine derivatives
Source: PLoS One. 2020 Aug 28;15(8):e0235102. doi: 10.1371/journal.pone.0235102 (PMC7455023; doi:10.1371/journal.pone.0235102)
Supplement: S1 File — Includes experimental details; 1H and 13C NMR spectra of S1; FTIR of S1; 1H and 13C NMR spectra of S2; FTIR of S2; 1H and 13C NMR spectra of S3; FTIR of S3; 1H and 31P NMR spectra of S4. (PDF) [file pone.0235102.s001.pdf]

Experimental Details for the Synthesis of the Phosphoramidites Used in this Work.

General Information. ^{1}H NMR and ^{13}C NMR spectra were recorded at 300, and 75 MHz, respectively. IR spectra were recorded on a diamond ATR sampler using powders of pure materials, or of photoreactions at various time intervals. Methylene chloride was distilled over calcium hydride. Tetrahydrofuran was distilled over sodium and benzophenone. All other reagents were used as purchased without further purification. All intermediates and compounds analyzed for HRMS were carried out via ESI/APCI. UV-vis spectroscopy was carried out on a Perkin Elmer λ-650 UV/vis spectrometer. The unusual solubility of inosine and its derivatives was pointed out in a previous study, where the synthesis for the phosphoramidite of I is reported,[1,2] and is something that was experienced for the 8-oxoI and 8-BrI derivatives.

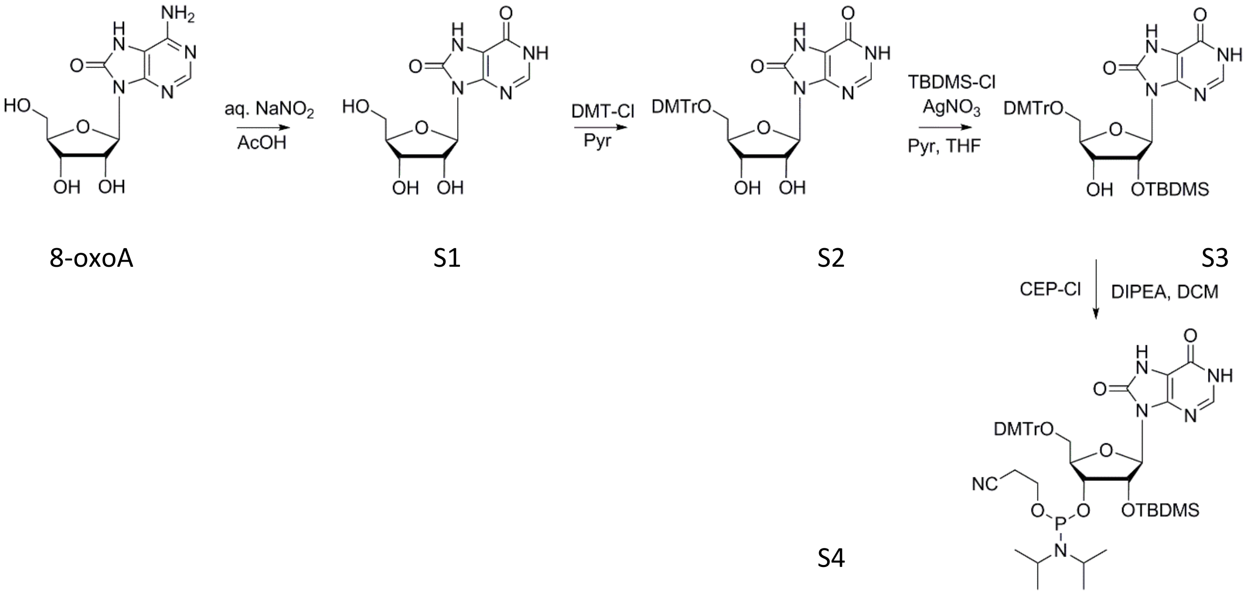

8-oxo-7,8-dihydroinosine (S1) [3]:

8-oxo-adenosine (6.6 g, 23.3 mmol) was added to a flask charged with a stirring bar, and dissolved in glacial acetic acid (120 mL). In a separate flask $NaNO_2$ (9.7 g, 140.6 mmol) was dissolved in water (45 mL) and the resultant solution was slowly added to the nucleoside solution while venting evolved gas over the first two hours. Reaction was left stirring at room temperature overnight, followed by bubbling with a stream of air for 3 hours. The residual solvent was concentrated under reduced pressure and the resultant solid was precipitated in 250 mL of 1:1 ethanol/water. The obtained powder was isolated by vacuum filtration to yield **8-oxoI** in the form of a yellowish solid 5 g (17.6 mmol, 65-90%). ^{1}HNMR (DMSO d-6): δ 7.94 (s, 1H), 5.67 (d, 1H), 4.83 (t, 1H), 4.12 (t, 1H), 3.86 (q, 1H), 3.60 (dd, 1H), 3.46 (dd, 1H). ^{13}CNMR (DMSO d-6): δ 160.49, 152.75, 150.82, 145.44, 108.60, 86.25, 71.84, 71.23, 63.16. FTIR (cm^{-1}): 3224.14, 2713.66, 1640, 1624.90, 1578.48. HRMS (m/z): 284.0768. All obtained spectra was in agreement with previously reported data.

5'-O-(4,4'-dimethoxytrityl)-8-oxo-7,8-dihydroinosine (S2):

8-oxo-inosine (1.5523 g, 5.5 mmol) was azeotropically dried over anhydrous pyridine (9 mL). Anhydrous pyridine (60 mL) was added to dry solid and cooled to 0 °C. 4,4′-dimethoxytrityl chloride (2.003 g, 5.9 mmol) was added in two portions under an atmosphere of argon. Reaction was stirred overnight and quenched with deionized water (100 mL), followed by extraction with dichloromethane (3 × 100 mL). Organic layers were combined and washed with brine (3 × 100 mL). Organic layers were then concentrated under reduced pressure to yield an oil. Purification via column chromatography was carried out using a gradient from 100% dichloromethane to 20% methanol in dichloromethane. Fractions were analyzed by TLC with an eluent of 20 % methanol in dichloromethane. Fractions were combined and concentrated under reduced pressure to yield S2 in the form of a white foam (0.500 g, 0.852 mmol, 10%) ^{1}HNMR (DMSO

d-6): δ 11.41 (s, 1H), 7.84 (s, 1H), 7.37 (d, 2H), 7.24 (m, 7H), 6.82 (m, 4H), 5.68 (d, 1H), 5.28 (d, 1H) 5.00 (d, 1H), 4.77 (t, 1H), 4.31 (t, 1H), 3.94 (t, 1H), 3.15 (m, 2H). ^{13}CNMR (DMSO d-6): δ 158.45, 152.13, 151.33, 145.50, 144.65, 136.19, 130.14, 128.23, 127.00, 113.48, 109.10, 86.86, 85.75, 82.84, 71.21,70.80, 64.52, 55.48, 46.21. FTIR (cm^{-1}): 3037.14, 2930.33, 1711.11, 1673.69, 1606.33, 1556.93, 1507.17, 1442.00. HRMS (m/z): 586.2070

2'-(t-butyldimethylsilyl)-5'-O-(4,4'-dimethoxytrityl)-8-oxo-7,8-dihydroinosine (S3):

S2 (1.5615 g, 2.7 mmol) and $AgNO_3$ (0.545 g, 3.2 mmol) were added to a foil covered flame dried flask charged with a stirring bar and the combined contents were placed under reduced pressure for 30 minutes. Anhydrous tetrahydrofuran (36 mL) and pyridine (5.45 mL) were added under an atmosphere of argon. Tert-butyldimethylchlorosilane (0.201 g, 3.3 mmol) was added and stirred over five hours. A second portion of TBDMS-Cl (0.4016 g, 1.3 mmol) and $AgNO_3$ (0.226 g, 1.3 mmol) were added and left stirring overnight. The resulting suspension was filtered and the filtrite was recollected and partitioned with ethyl acetate (50 mL) and 20% $NaHCO_3$ (20 mL). The organic layer was washed over deionized water (2 × 25 mL), and brine (1 × 25 mL). Organic layers were concentrated to a crude oil and purified by column chromatography using a gradient to 3% methanol in ethyl acetate. The desired product was the first spot to elute off the column. Fractions containing the desired regioisomer were combined and concentrated to yield a white foam corresponding to S3 (0.5 g, 0.713 mmol, 27%). ^{1}HNMR (DMSO d-6): δ 11.43 (s, 1H), 8.58 (s, 1H), 7.80 (m, 2H), 7.38 (m, 4H), 7.25 (m, 6H), 6.84 (m, 4H), 5.69 (m, 1H) 4.94 (m, 1H), 4.85 (m, 1H), 4.24 (m, 1H), 3.95 (t, 1H), 3.18 (m, 1H), 3.11 (m, 1H), 0.77 (s, 9H), -0.02 (s, 3H),-0.08 (s, 3H). ^{13}CNMR (DMSO d-6): 157.95, 151.54, 150.77, 146.57, 144.98, 144.42, 144.08, 136.06, 135.63, 129.70, 127.62, 126.50, 123.84, 113.00, 108.60, 85.96, 85.24, 82.56, 72.20, 70.17, 63.56, 54.95, 25.57, 12.87, -4.80, -5.23. FTIR (cm^{-1}): 3035.2, 2929.14, 2856.21, 1711.11, 1678.15, 1606.68, 1556.16, 1507.63, 1441.02. HRMS (m/z): 700.2964

2'-O-(t-butyldimethylsilyl)-3'-O-[(2-ethylcyano-N,N-diisopropylphosphoramidite)-5'-O-(4,4'-dimethoxytrityl)-8-oxo-7,8-dihydroinosine (S4):

S3 (0.3 g, 0.42 mmol) was added to a flame dried flask charged with a stirring bar and dried under vacuum for an hour. Dichloromethane (0.85 mL) and diisopropylethylamine (0.45 mL) were added under an atmosphere of argon. 2-Cyanoethyl N,N-diisopropylchlorophosphoramidite (CEP-Cl) (0.145 mL) was added to the solution and stirred at room temperature for one hour. After one hour additional CEP-Cl (0.15 mL) was added and the reaction was stirred for additional 30 minutes. The reaction mixture was then quenched over 20 % $NaHCO_3$ (20 mL) and extracted with dichloromethane (3 × 25 mL). The combined organic extracts were then washed with deionized water (2 × 25 mL) and brine (1 × 25 mL). The resultant organic layer was concentrated under reduced pressure and purified via column chromatography using a gradient from 40% to 60% acetone in dichloromethane. Fractions containing product were concentrated under reduced pressure to yield phosphoramidite S4 in the form of a white powder (0.15 g, 0.17 mmol, 39%). ^{1}HNMR ($CDCl_3$): δ 8.81 (d, 1H) 7.53 (d, 1H) 7.39 (m, 2H) 7.29 (m, 4H), 7.16 (m, 6H), 6.84 (m, 4H) 6.77 (m, 2H), 5.98 (d, 1H), 5.93 (d, 1H), 5.49 (m, 1H) 5.00 (m, 1H), 4.39 (m, 1H), 4.32 (m,1H), 4.15 (m, 1H), 3.81 (d, 6H), 3.74 (d, 6H), 3.50 (m, 1H) 3.21 (m, 1H), 0.88 (s, 9H), 0.83 (s, 9H), 0.10 (s, 3H), 0.00 (s, 3H), -0.03 (s, 3H), -0.15 (s, 3H).^{31}PNMR ($CDCl_3$): δ 150.72, 148.46. HRMS (m/z): 899.4036.

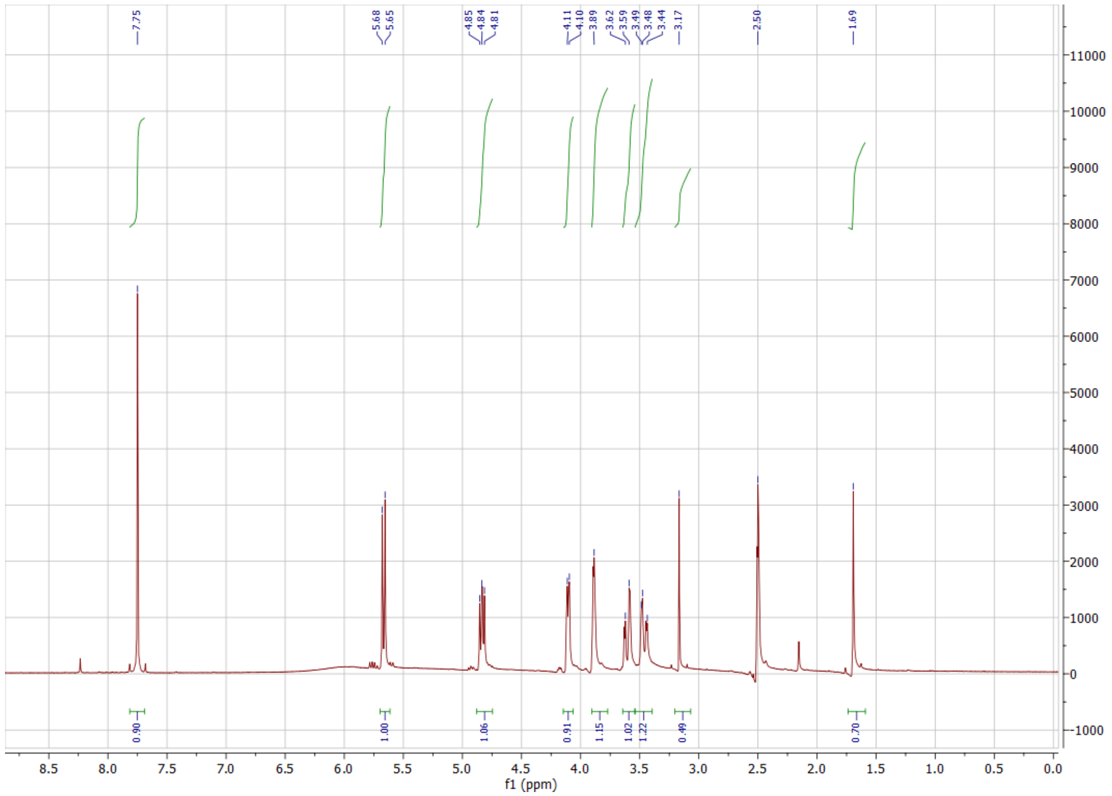

^{1}HNMR spectrum of S1

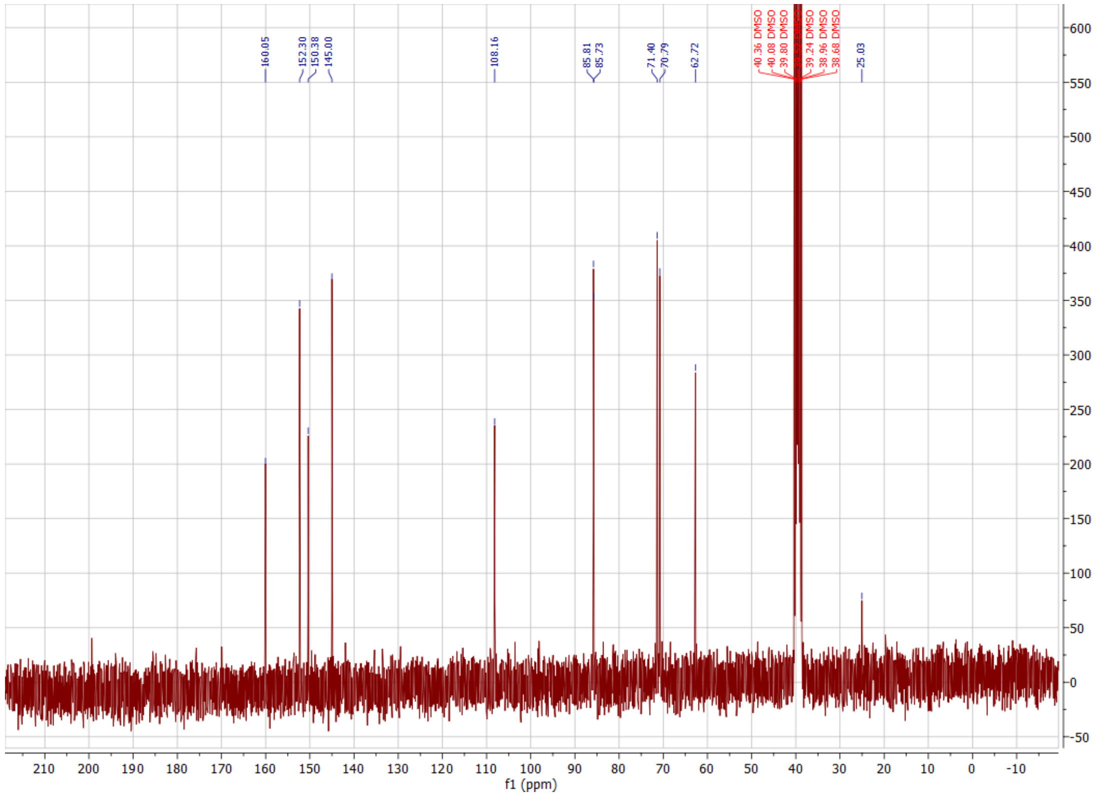

^{13}CNMR spectrum of S1

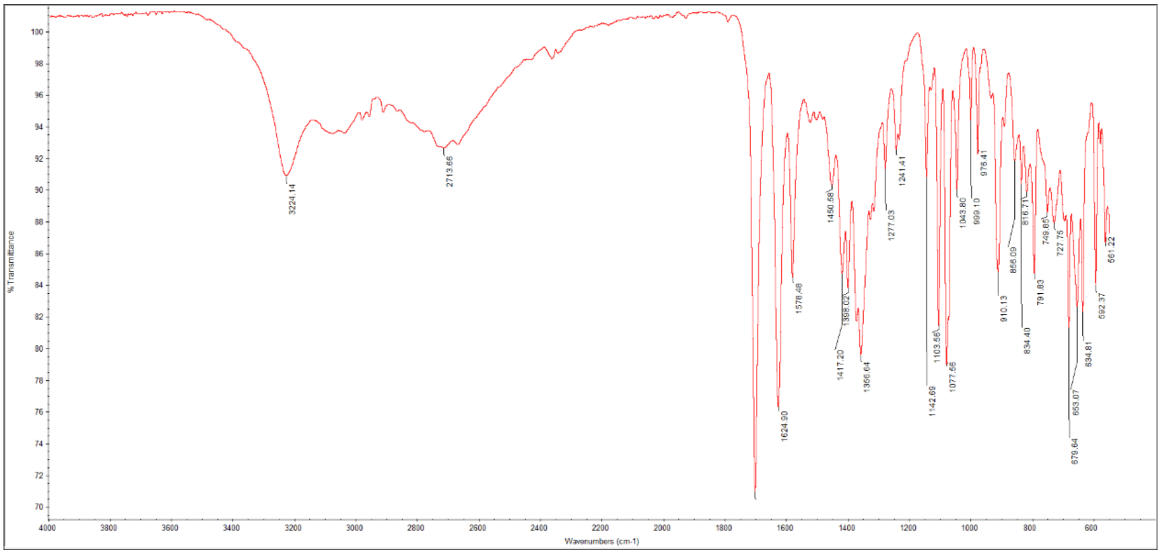

FTIR spectrum of S1

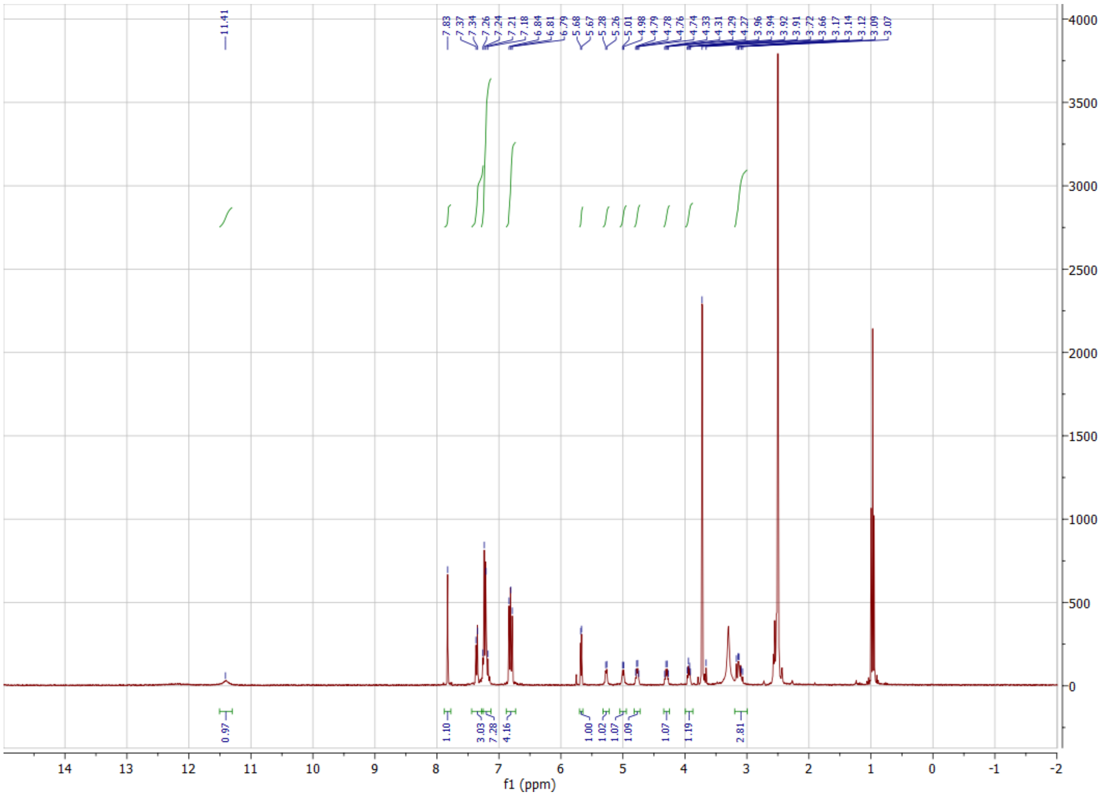

^{1}HNMR spectrum of S2

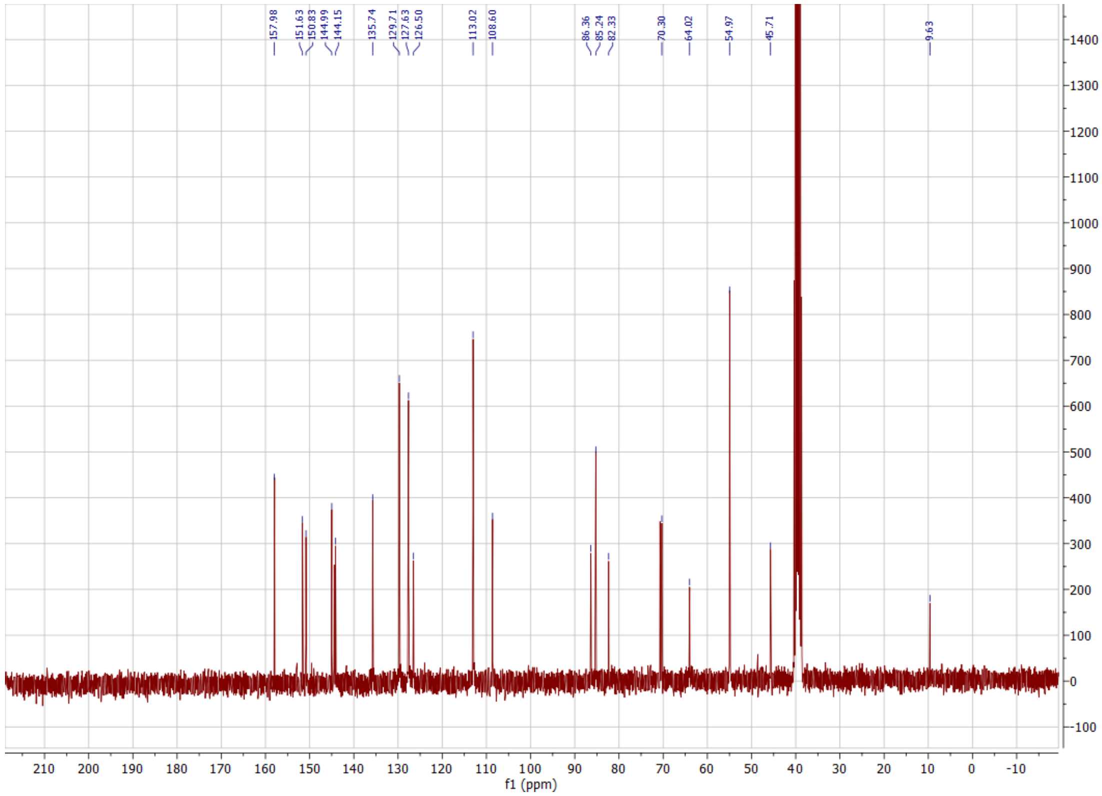

^{13}CNMR spectrum of S2

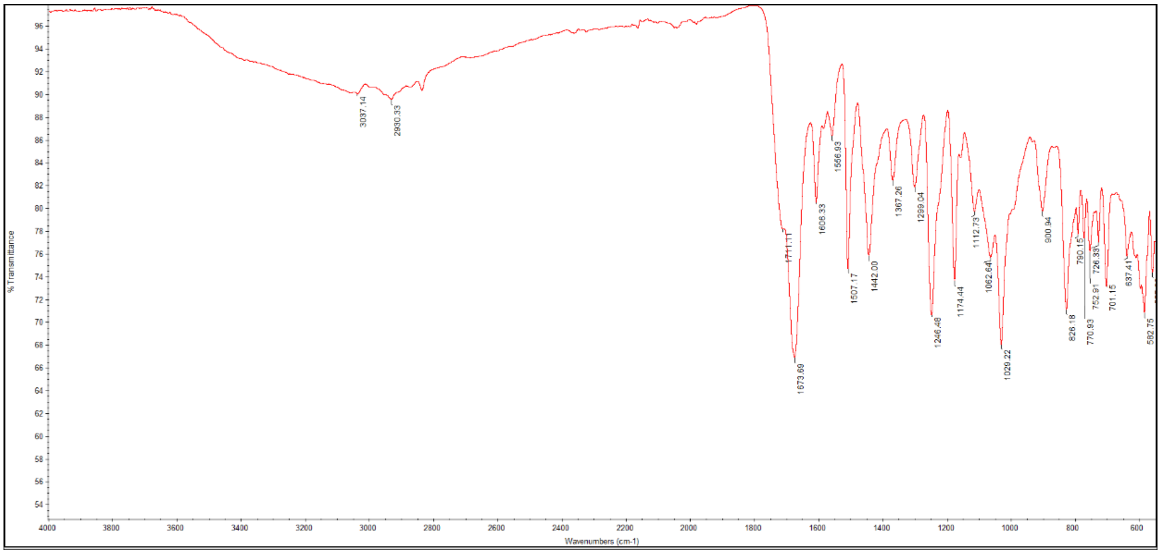

FTIR spectrum of S2

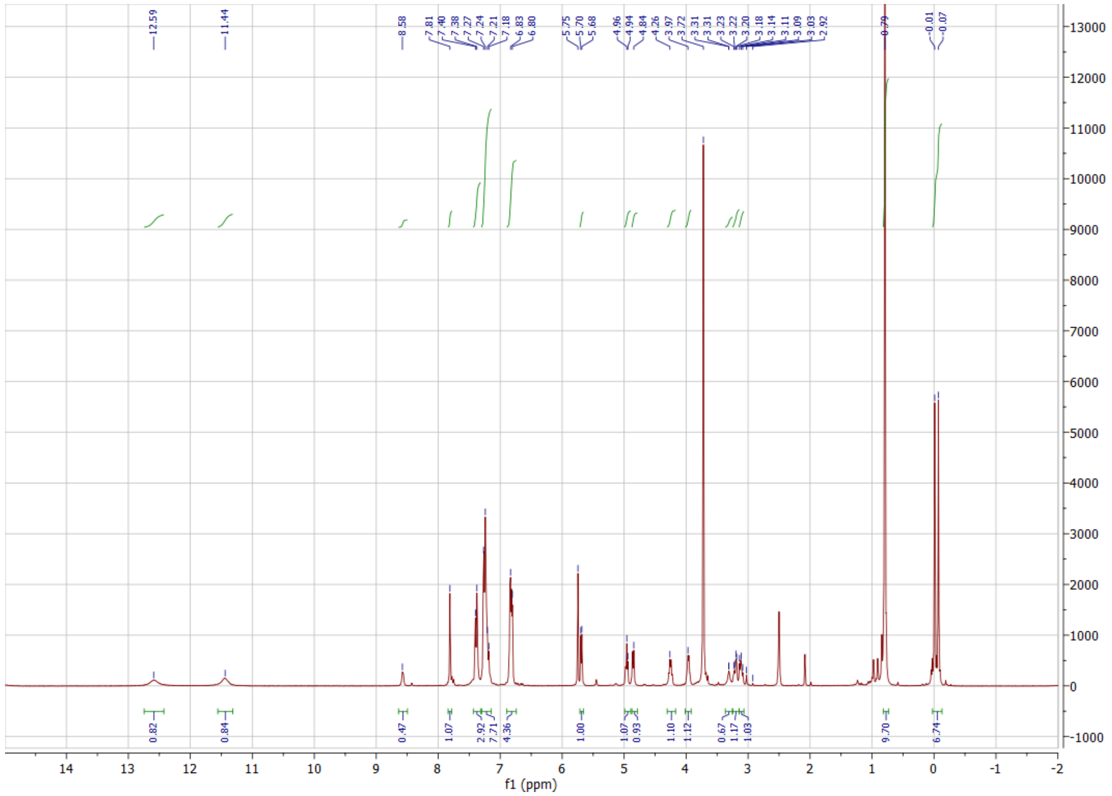

^{1}HNMR spectrum of S3

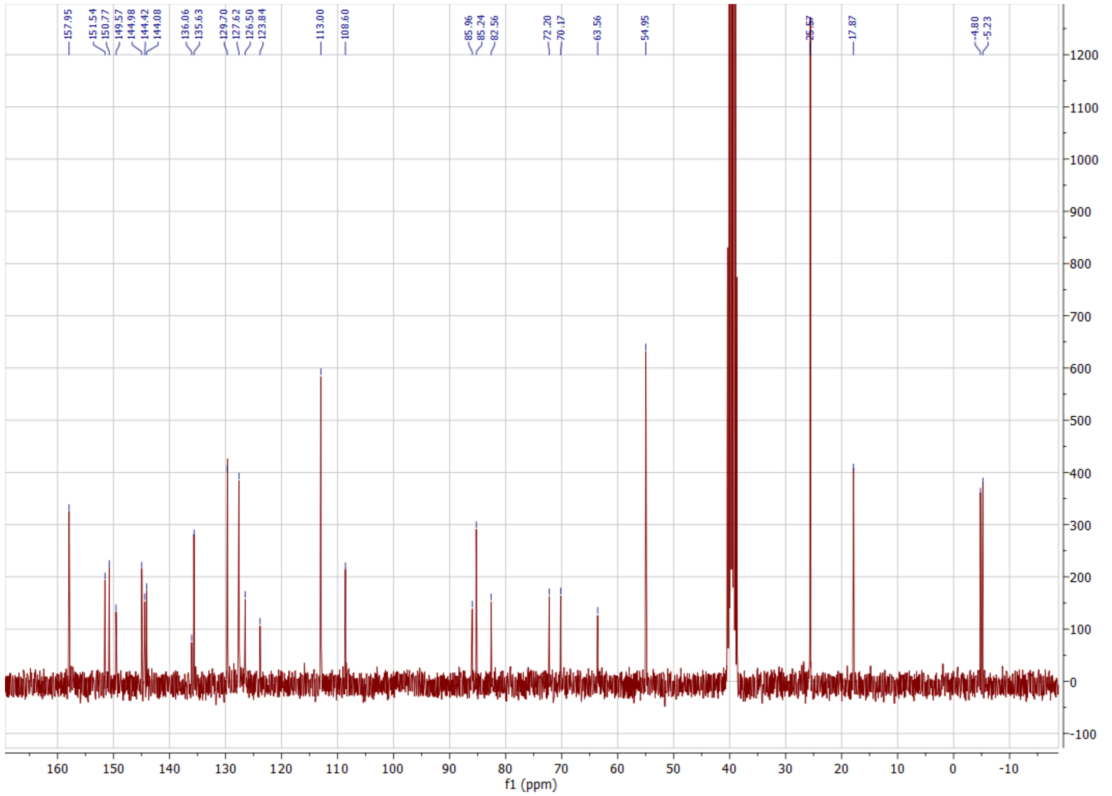

^{13}CNMR spectrum of S3

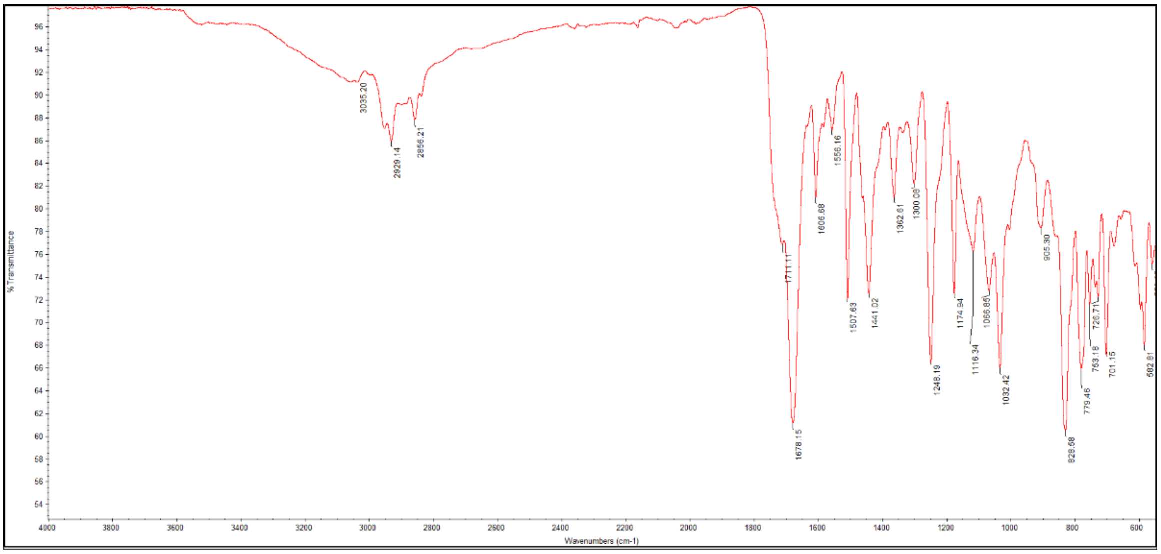

FTIR spectrum of S3

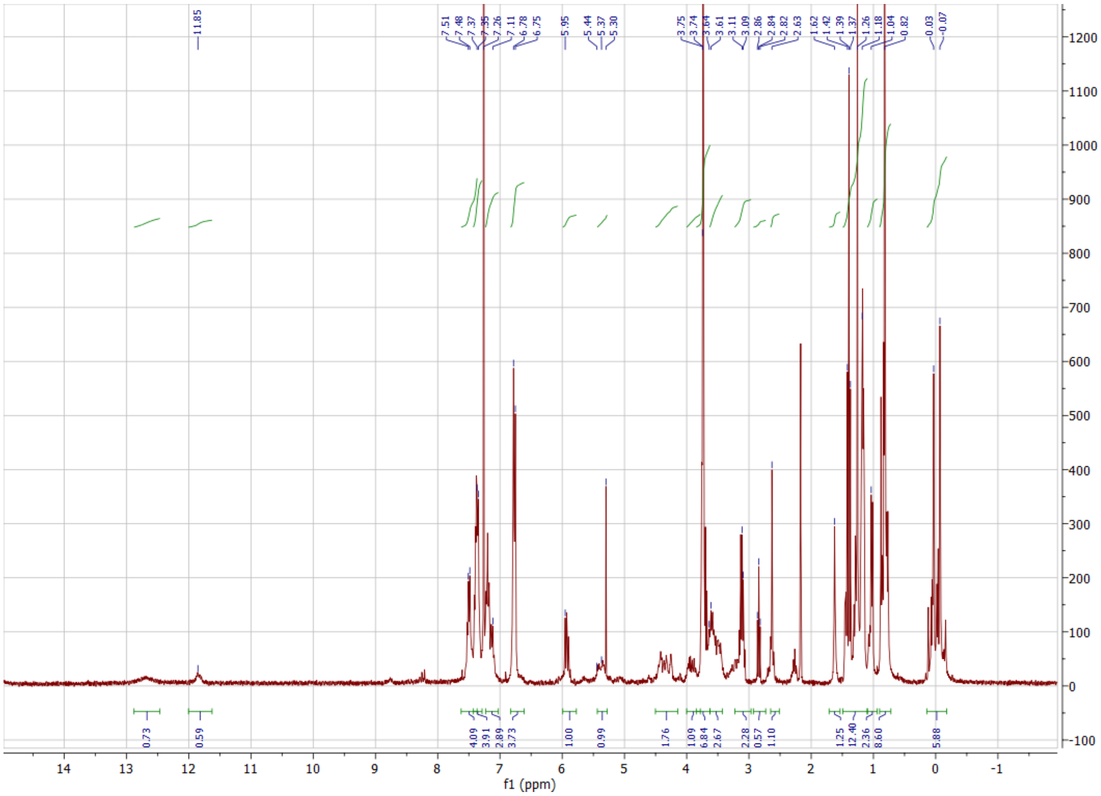

^{1}HNMR spectrum of S4

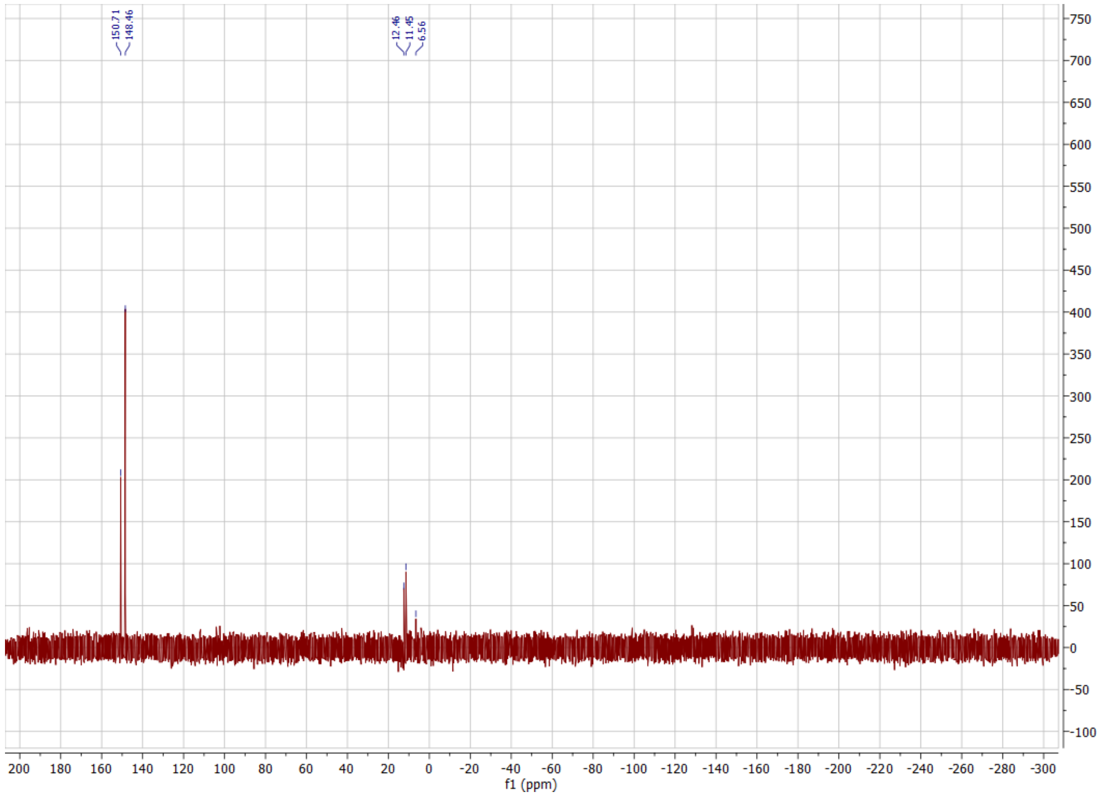

^{31}PNMR spectrum of S4

References

(1) Green, R.; Szostak, J. W.; Benner, S. A.; Rich, A.; Usman, N. Synthesis of RNA containing inosine: analysis of the sequence requirements for the 5' splice site of the *Tetrahymena* group I intron. *Nucleic Acids Res.* **1991**, 19, 4161-4166.

(2) Matulic-Adamic, J.; Beigelman, L. An improved synthesis of inosine 3'-phosphoramidite. *Syn. Commun.* **2000**, 30, 3963-3969.

(3) Lena, S.; Cremonini, M. A.; Federiconi, F.; Gottarelli, G.; Graziano, C.; Laghi, L.; Mariani, P.; Masiero, S.; Pieraccini, S.; Spada, G. P. The supramolecular helical architecture of 8-oxoinosine and 8-oxoguanosine derivatives. *Chem. Eur. J.* **2007**, 13, 3441-3449.
