# Supplementary material for: Translesion synthesis by AMV, HIV, and MMLVreverse transcriptases using RNA templates containing inosine, guanosine, and their 8-oxo-7,8-dihydropurine derivatives
Source: PLoS One. 2020 Aug 28;15(8):e0235102. doi: 10.1371/journal.pone.0235102 (PMC7455023; doi:10.1371/journal.pone.0235102)
Supplement: S2 File — Includes experimental details; 1H and 13C NMR spectra of S5; FTIR of S5; 1H and 13C NMR spectra of S6; FTIR of S6; 1H and 13C NMR spectra of S7; FTIR of S7; 1H and 31P NMR spectra of S8. (PDF) [file pone.0235102.s002.pdf]

Experimental Details for the Synthesis of the Phosphoramidites Used in this Work.

General Information. ^{1}H NMR and ^{13}C NMR spectra were recorded at 300, and 75 MHz, respectively. IR spectra were recorded on a diamond ATR sampler using powders of pure materials, or of photoreactions at various time intervals. Methylene chloride was distilled over calcium hydride. Tetrahydrofuran was distilled over sodium and benzophenone. All other reagents were used as purchased without further purification. All intermediates and compounds analyzed for HRMS were carried out via ESI/APCI. UV-vis spectroscopy was carried out on a Perkin Elmer λ-650 UV/vis spectrometer. The unusual solubility of inosine and its derivatives was pointed out in a previous study, where the synthesis for the phosphoramidite of I is reported,[1,2] and is something that was experienced for the 8-oxoI and 8-BrI derivatives.

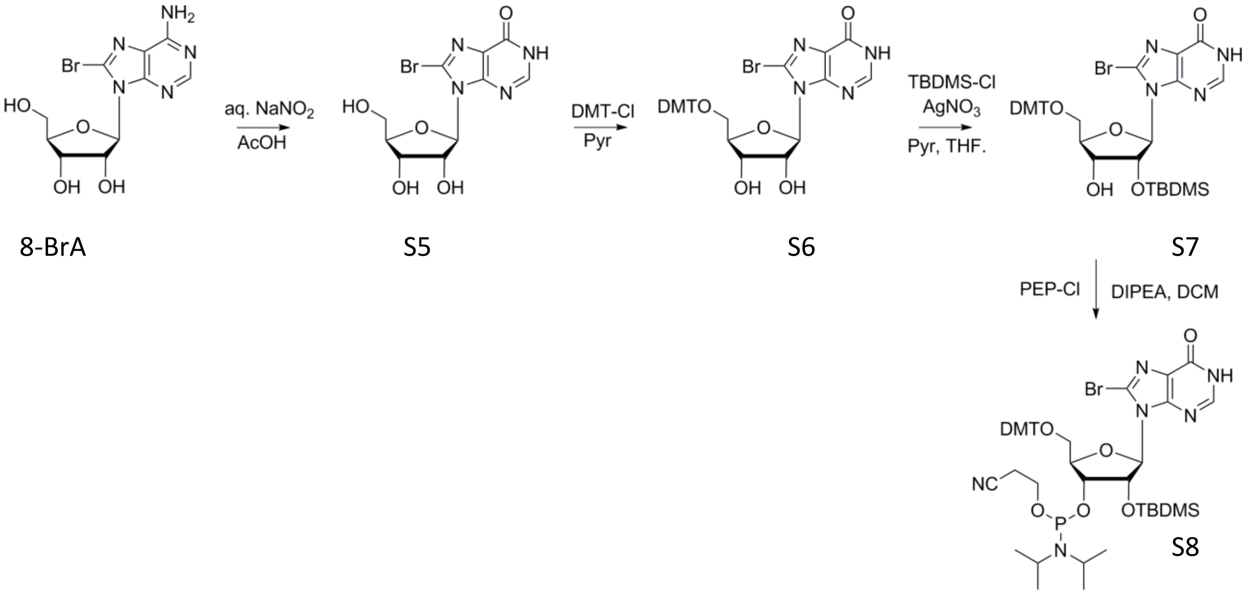

8-Bromoinosine (S5)[4]:

8-bromo-adenosine (8.5709 g, 24.6 mmol) was added to flask charged with a stirring bar and dissolved in glacial acetic acid (150 mL). In a separate flask $NaNO_2$ (10.1902 g, 147.7 mmol) was dissolved in 50 mL deionized water and the resultant solution was slowly added to the nucleoside solution, while venting the evolved gas over ca. 2 h. The reaction mixture was stirred at room temperature overnight, and bubbled with a flow of air over 3 h. The residual solvent was concentrated under reduced pressure and a solid precipitated following the addition of a 100 mL of 4:1 ethanol/water solution. An orange solid was then obtained upon filtration to yield 8-bromoinosine, 5 g (18.0 mmol, 50-85%) ^{1}HNMR (DMSO d-6): δ 7.94 (s, 1H), 5.67 (d, 1H), 4.83 (t, 1H), 4.12 (t, 1H), 3.86 (q, 1H), 3.60 (dd, 1H), 3.46 (dd, 1H). ^{13}CNMR (DMSO d-6): δ 155.58, 149.21, 146.53, 125.98, 125.37, 90.49, 86.30, 71.21, 70.50, 61.89. FTIR (cm^{-1}): 3492.73, 3347.71, 3180.44, 2924.03, 1676.00, 1588.02, 826.40. HRMS (m/z): 284.0768.

5'-O-(4,4'-dimethoxytrityl)-8-bromo-inosine (S6):

8-bromoinosine (6.086 g, 17.5 mmol) was azeotropically dried over anhydrous pyridine (35 mL). Anhydrous pyridine (90 mL) was added to dry solid and cooled to 0 °C. 4,4′-dimethoxytrityl chloride (6.43 g, 19.0 mmol) was added under an atmosphere of argon. The resulting solution was stirred overnight and quenched over 20% $NaHCO_3$ (100 mL) followed by consecutive washes with ethyl acetate (3 × 100 mL). The combined organic residues were combined and washed with deionized water (3 × 100 mL) and brine (1 × 100 mL). The organic extracts were then concentrated under reduced pressure to an oil. Purification was then achieved via column chromatography using a gradient from 100% DCM to 20% methanol in dichloromethane. Fractions were analyzed by TLC with an eluent of 20 % methanol in dichloromethane. The fractions of interest were combined and concentrated under reduced pressure to yield compound S6 in the form of a white foam (8.5455 g, 13.5 mmol, 75%) ^{1}HNMR (DMSO d-6): δ 11.41 (s, 1H), 7.84 (s, 1H), 7.37 (d, 2H), 7.24 (m, 7H), 6.82 (m, 4H), 5.68 (d, 1H), 5.28 (d, 1H) 5.00 (d, 1H), 4.77 (t, 1H), 4.31 (t, 1H), 3.94 (t, 1H), 3.15 (m, 2H). ^{13}CNMR (DMSO d-6): δ 157.96, 155.26, 149.06, 145.83, 144.88, 135.68, 135.57, 129.70, 129.58, 127.66, 126.55, 126.27, 125.20, 112.98,

85.23, 83.39, 71.08, 70.03, 63.30, 54.99, 48.60, 45.71. FTIR (cm^{-1}): 3056.08, 2930.36, 2834.62, 1682.86, 1606.15, 1584.34, 1507.14, 826.40. HRMS (m/z): 586.2070

2'-(t-butyldimethylsilyl)-5'-O-(4,4'-dimethoxytrityl)-8-bromo-inosine (S7):

S6 (8.5 g, 13.1 mmol) and $AgNO_3$ (2.89 g, 17 mmol) were added to a foil covered flame dried flask charged with a stirring bar and dried under reduced pressure for 30 minutes. Anhydrous tetrahydrofuran (36 mL) and pyridine (8 mL) were added under an atmosphere of argon. Tert-butyldimethylchlorosilane (2.367 g, 15.7 mmol) was added quickly under an atmosphere of argon and left to react for four hours. Additional TBDMS-Cl (1.112 g, 7.4 mmol) and $AgNO_3$ (0.986 g, 5.8 mmol) were added and the obtained suspension was stirred overnight. The milky suspension was then filtered, and the collected filtrite was concentrated under reduced pressure to an oily solid. Ethyl acetate (100 mL) and 20% $NaHCO_3$ (100 mL) were added to partition the crude product. Aqueous layer was extracted over ethyl acetate (5 × 100 mL). Organic layer was washed with deionized water (2 × 100 mL) and brine (1 × 100 mL). Organic layer was concentrated and the residues were purified via column chromatography using a gradient of 100% dichloromethane to 15% acetone in dichloromethane. Fractions containing the desired regioisomer were concentrated under reduced pressure to yield nucleoside S7 in the form of a white foam (1.1 g, 1.4 mmol, 11.1%) ^{1}HNMR (DMSO d-6): δ 11.43 (s, 1H), 8.58 (s, 1H), 7.80 (m, 2H), 7.38 (m, 4H), 7.25 (m, 6H), 6.84 (m, 4H), 5.69 (m, 1H) 4.94 (m, 1H), 4.85 (m, 1H), 4.24 (m, 1H), 3.95 (t, 1H), 3.18 (m, 1H), 3.11 (m, 1H), 0.77 (s, 9H), -0.02 (s, 3H),-0.08 (s, 3H). ^{13}CNMR (DMSO d-6): 157.99, 155.17, 149.61, 149.11, 145.83, 144.90, 136.1, 135.55, 129.73, 127.67, 126.58, 125.28, 123.89, 113.03, 90.43, 85.30, 83.95, 72.71, 69.95, 62.99, 54.99, 25.48, 17.78, -4.83, -5.43. FTIR (cm^{-1}): 3056.34, 2929.99, 1682.85, 1606.46, 1584.42, 1507.44, 827.02. HRMS (m/z): 700.2964

2'-O-(t-butyldimethylsilyl)-3'-O-[(2-ethylcyano-N,N-diisopropylphosphoramidite)-5'-O-(4,4'-dimethoxytrityl)-8-bromo-inosine (S8):

Nucleoside S7 (1.0128 g, 1.3 mmol) was added to a flame dried flask charged with a stirring bar and dried under reduced pressure for an hour. Dichloromethane (2.15 mL) and diisopropylethylamine (1.38 mL) were added under an atmosphere of argon. 2-Cyanoethyl N,N-diisopropylchlorophosphoramidite (0.45 mL) was added to the clear solution and stirred at room temperature for one hour. Additional 2-Cyanoethyl N,N-diisopropylchlorophosphoramidite (0.15 mL) was added and the reaction was stirred for another 30 minutes. The reaction was then quenched over 20 % $NaHCO_3$ (20 mL) and extracted with dichloromethane (3 × 25 mL). The organic layer was combined and washed with deionized water (2 × 25 mL) and brine (1 × 25 mL). The resultant solution was concentrated under reduced pressure followed by purification via column chromatography using a gradient from 0% to 10% acetone in dichloromethane. The fractions of interest were concentrated under reduced pressure to yield phosphoramidite S8 in the form of a white foam (0.96 g, 1.0 mmol, 75.6%). ^{1}HNMR ($CDCl_3$): δ 12.55 (s, 1H) 8.02 (d, 1H) 7.45 (d, 2H) 7.37 (m, 5H) 7.21 (m, 2H), 6.77 (m, 4H), 5.99 (m, 1H) 5.38 (m, 1H), 5.42 (m, 1H), 4.44 (m, 2H), 4.37 (m, 2H) 4.21 (m, 2H), 4.15 (m, 2H), 3.65 (s, 6H), 3.55 (m, 3H), 3.81 (d, 6H), 3.74 (d, 6H), 3.50 (m, 1H) 3.21 (m, 1H), 0.77 (s, 9H), -0.04 (s, 3H), -0.25 (s, 3H).^{31}PNMR ($CDCl_3$): 151.49, 148.52. HRMS (m/z): ^{79}Br 961.3349, ^{81}Br 963.3349

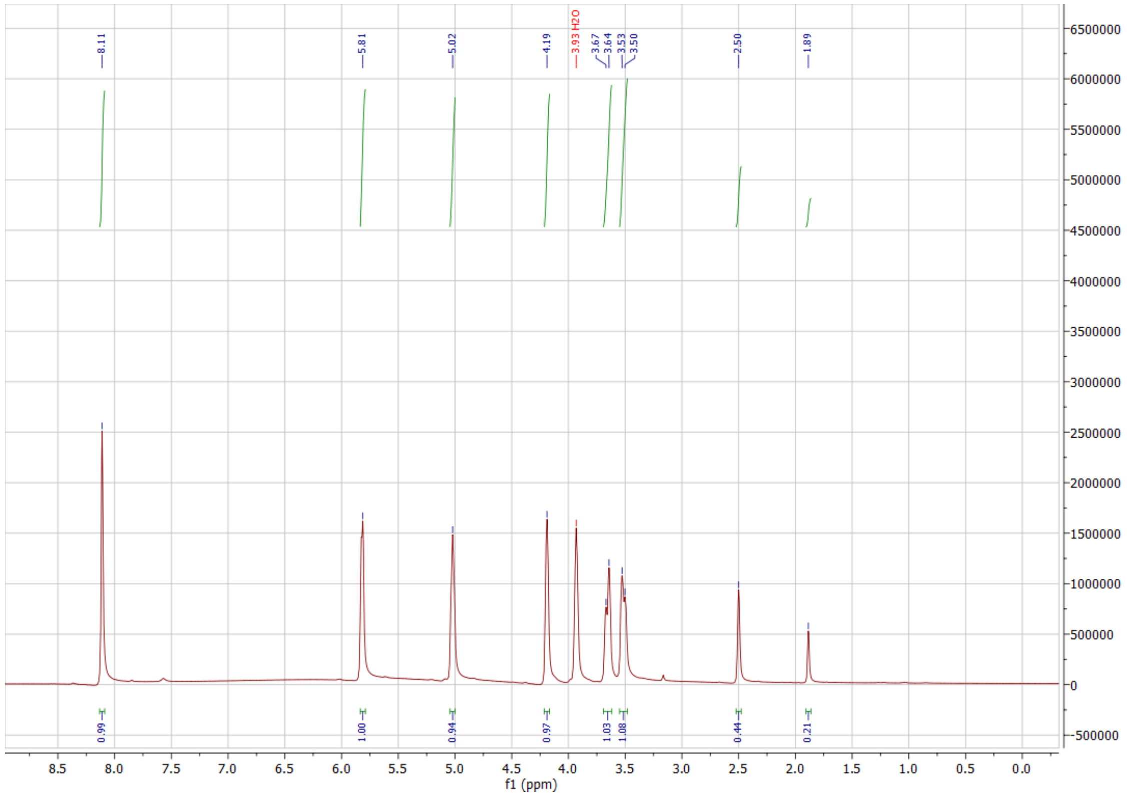

^{1}HNMR spectrum of S5

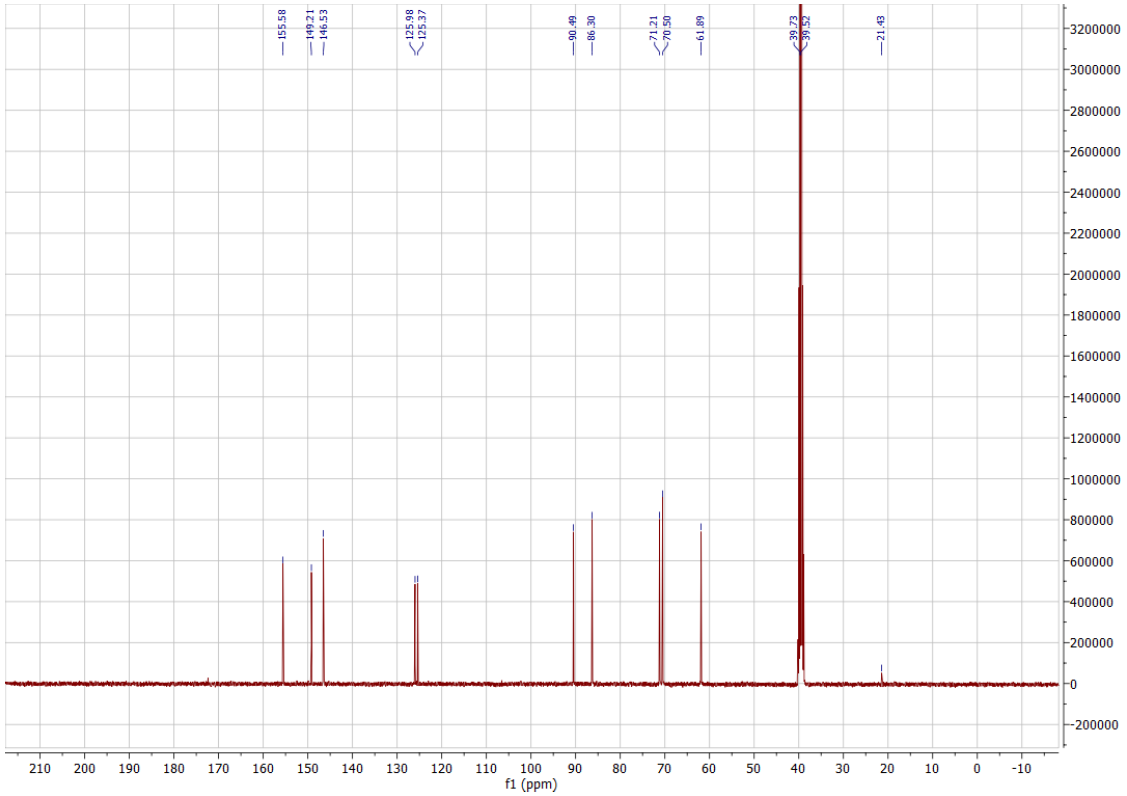

^{13}CNMR spectrum of S5

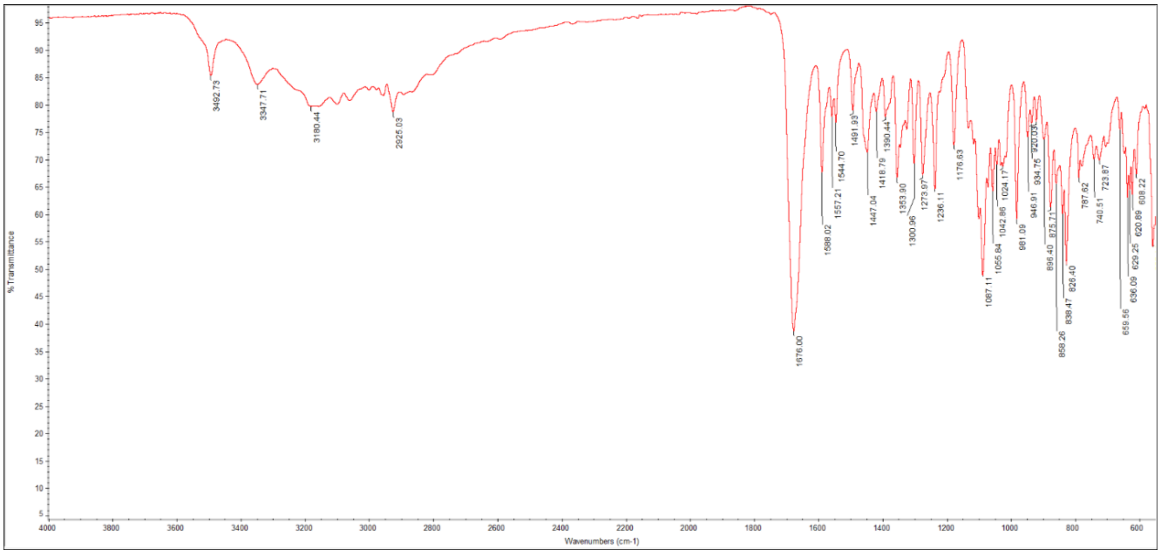

FTIR spectrum of S5

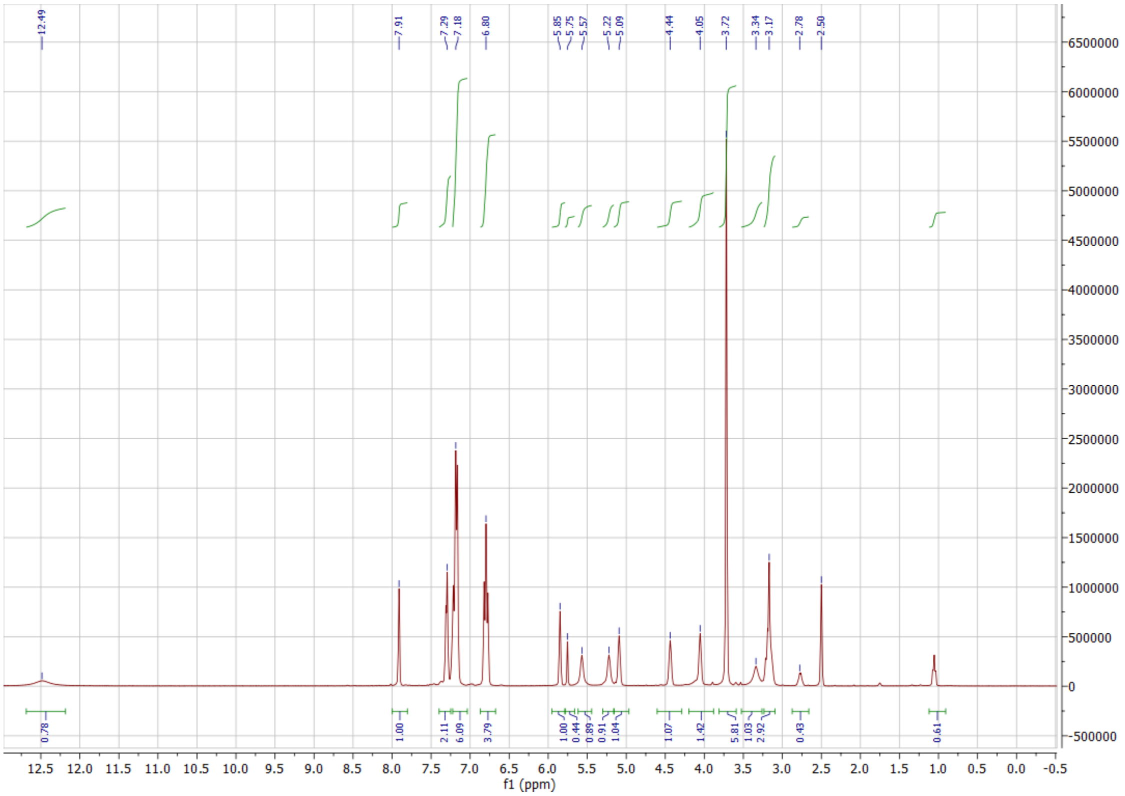

^{1}HNMR spectrum of S6

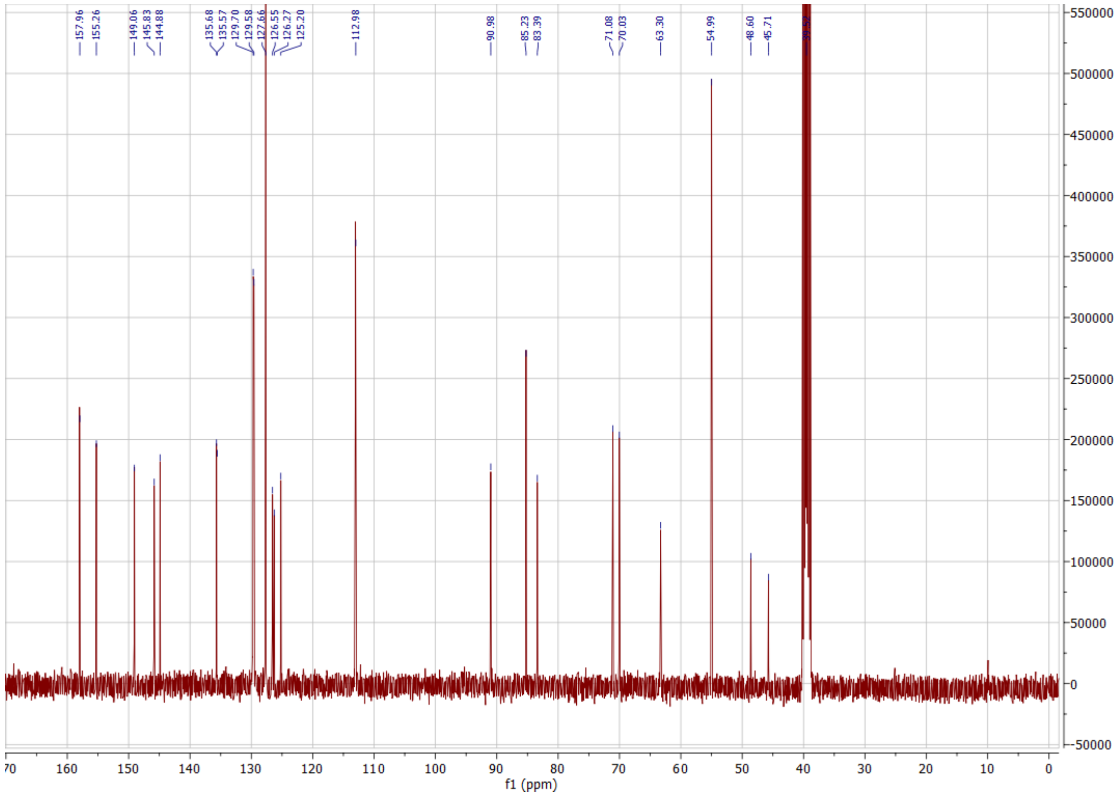

^{13}CNMR spectrum of S6

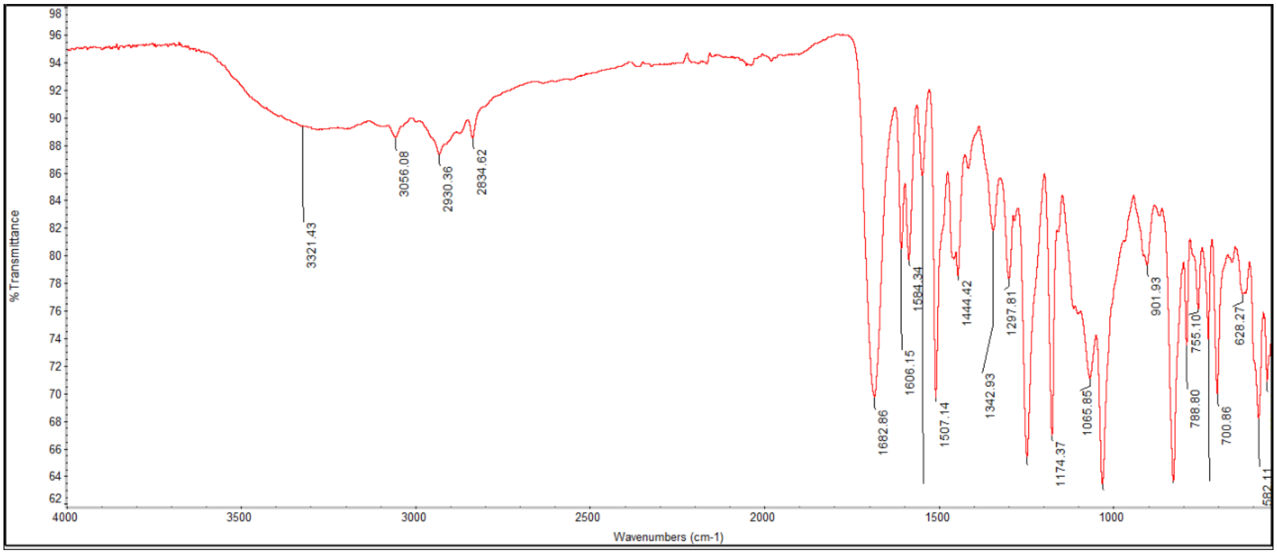

FTIR spectrum of S6

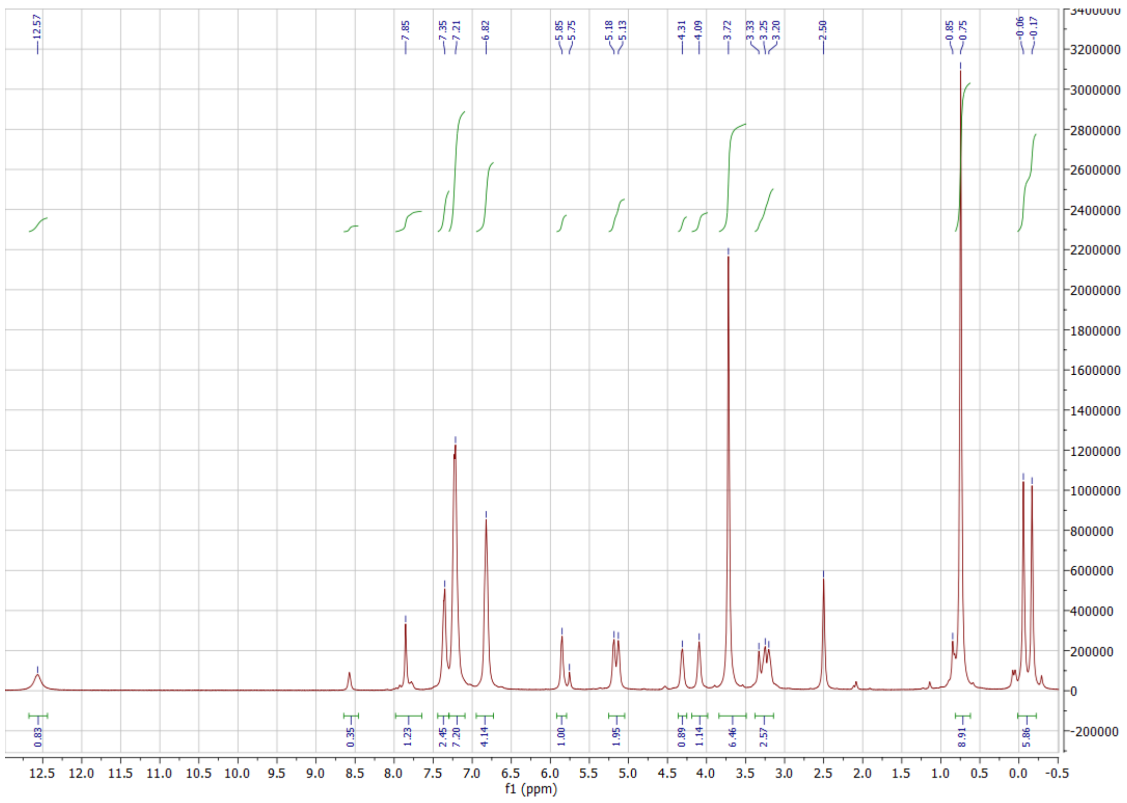

^{1}HNMR spectrum of S7

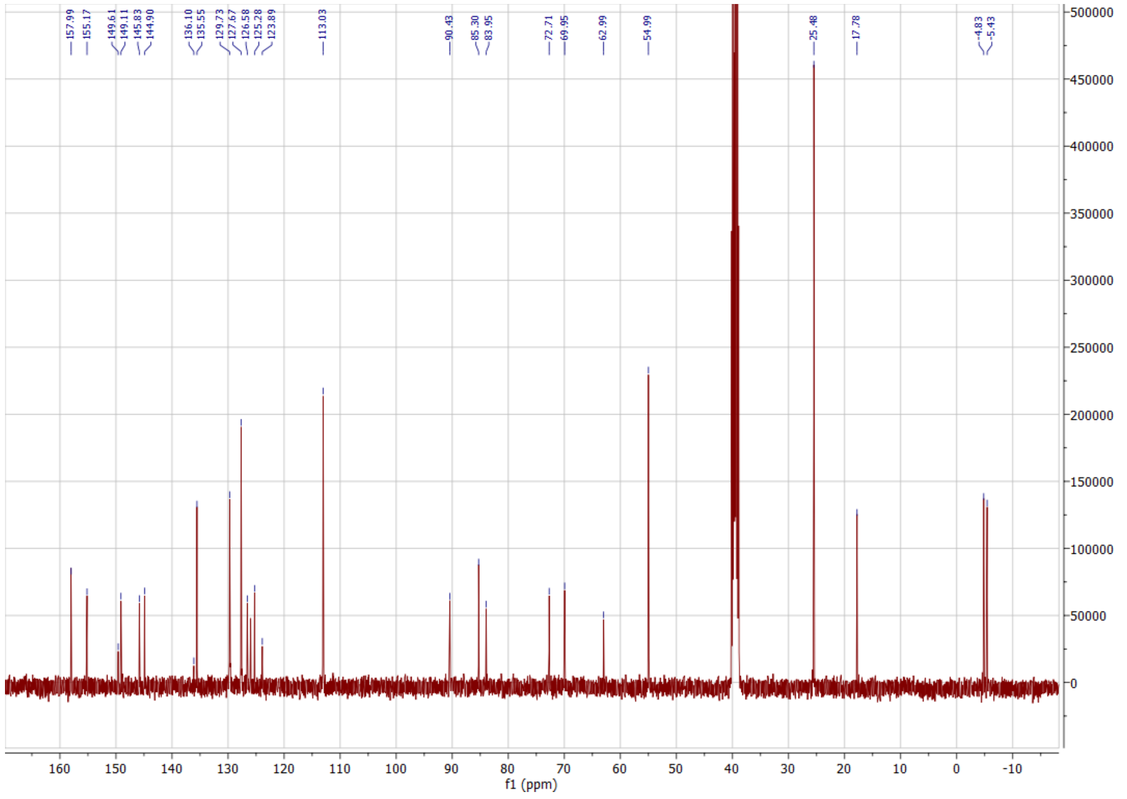

^{13}CNMR spectrum of S7

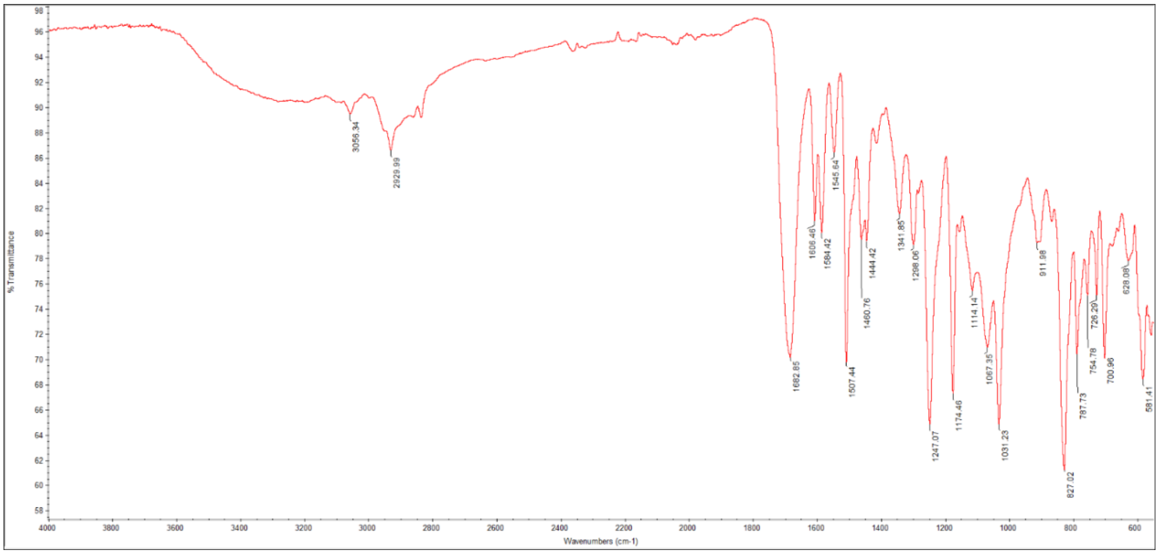

FTIR spectrum of S7

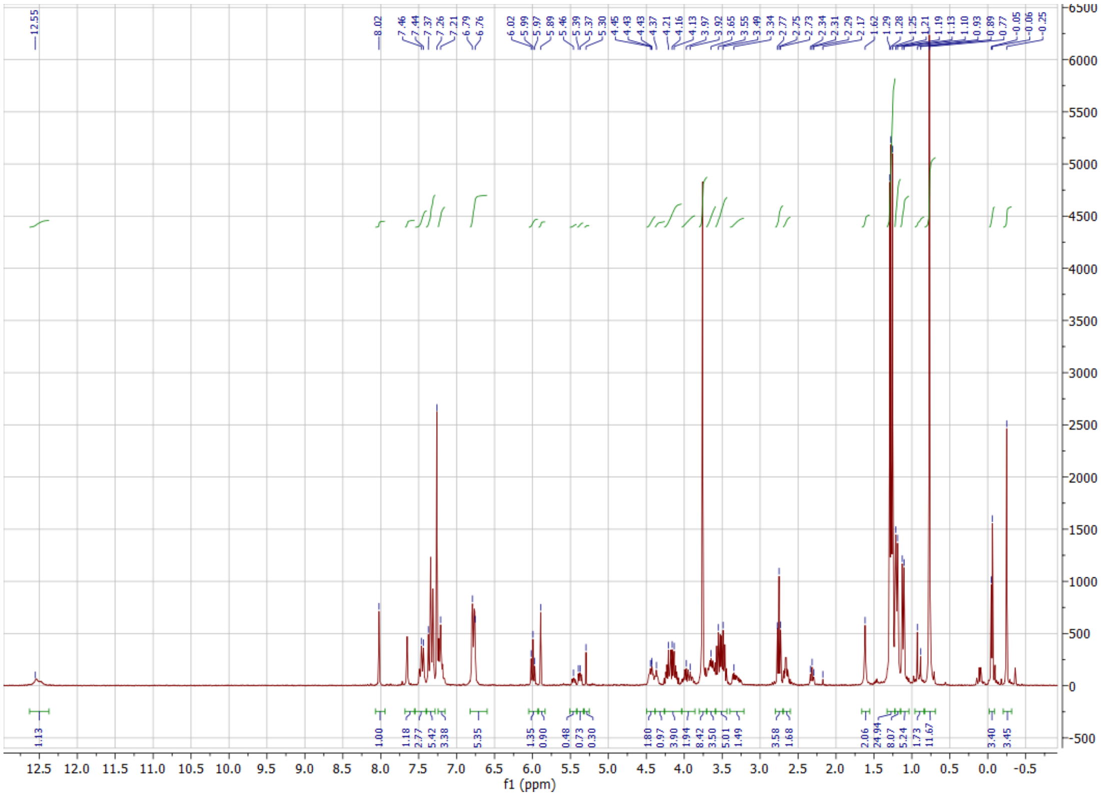

^{1}HNMR spectrum of S8

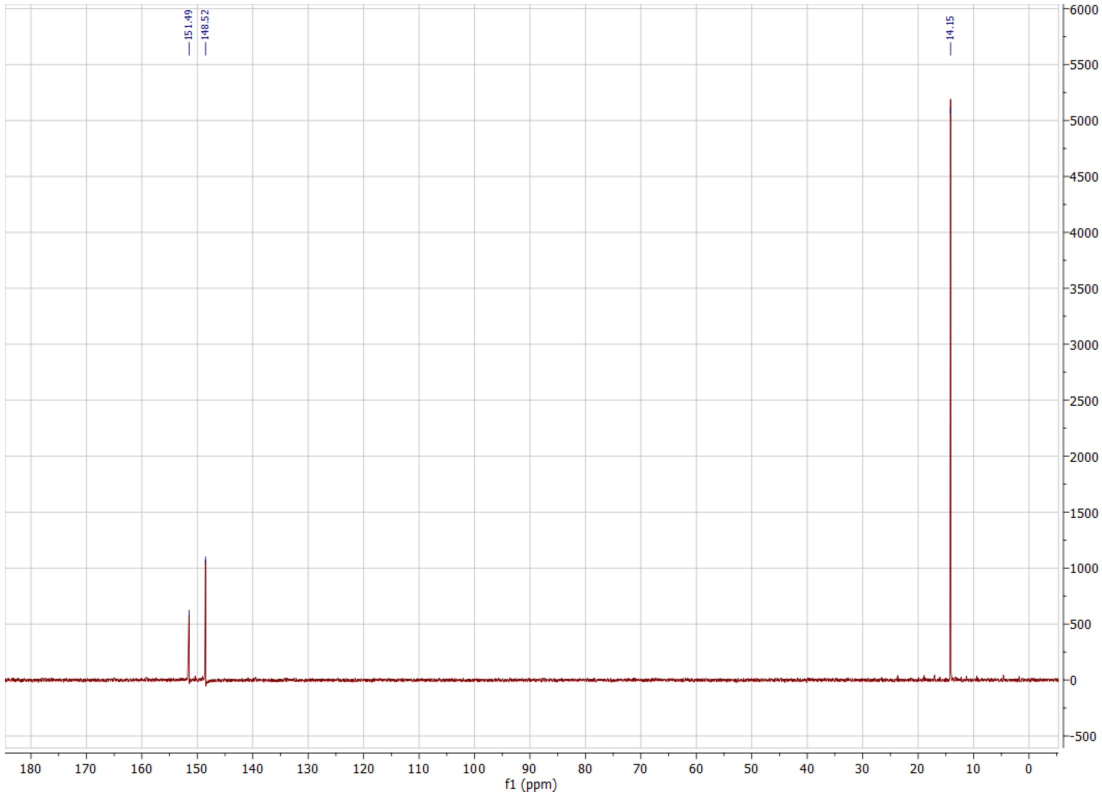

^{31}PNMR spectrum of S8

References

(1) Green, R.; Szostak, J. W.; Benner, S. A.; Rich, A.; Usman, N. Synthesis of RNA containing inosine: analysis of the sequence requirements for the 5’ splice site of the *Tetrahymena* group I intron. *Nucleic Acids Res.* **1991**, 19, 4161-4166.

(2) Matulic-Adamic, J.; Beigelman, L. An improved synthesis of inosine 3’-phosphoramidite. *Syn. Commun.* **2000**, 30, 3963-3969.

(3) Lena, S.; Cremonini, M. A.; Federiconi, F.; Gottarelli, G.; Graziano, C.; Laghi, L.; Mariani, P.; Masiero, S.; Pieraccini, S.; Spada, G. P. The supramolecular helical architecture of 8-oxoinosine and 8-oxoguanosine derivatives. *Chem. Eur. J.* **2007**, 13, 3441-3449.

(4) Lin, T-S.; Cheng, J-C.; Ishiguro, K.; Sartorelli, A. C. Purine and 8-substituted purine arabinofuranosyl and ribofuranosyl nucleoside derivatives
