# Supplementary material for: Translesion synthesis by AMV, HIV, and MMLVreverse transcriptases using RNA templates containing inosine, guanosine, and their 8-oxo-7,8-dihydropurine derivatives
Source: PLoS One. 2020 Aug 28;15(8):e0235102. doi: 10.1371/journal.pone.0235102 (PMC7455023; doi:10.1371/journal.pone.0235102)
Supplement: S3 File — Details and mass spectra of oligonucleotides 1–4 and 9. (PDF) [file pone.0235102.s003.pdf]

To obtain the spectra for oligonucleotide **9**, the sample was sent to the facilities at Colorado State University and the following was obtained, which justifies a difference of ~ 6 Da.

In addition, the additional peaks observed for samples **3** and **4** can be rationalized from decomposition upon shipping and/or handling. This is consistent with the appearance of one peak corresponding to the oligonucleotide of interest on samples taken elsewhere, which were spotted in our laboratory.

Sample Preparation:

The provided matrix components were combined in a 1:1 ratio to create 25mM 2,4,6-trihydroxyacetophenone monohydrate, 10mM ammonium citrate, 300mM ammonium fluoride, 50% ACN. 20µl of 20µM (e.g. 20pmol/µl) sample were provided. A total of 200pmol was used for zip tip clean up following instructions provided by MR: µC18 Zip Tips (Millipore) were activated using 50% ACN (10µl x2) followed by equilibration in 0.1% TFA (10µl x2). 200pmol (e.g. 10µl) of sample were then applied to the tip and loaded/aspirated for 10 cycles. The tip was then washed with 0.1% TFA (10µl x2) and water (10µl x2) followed by elution into 10µl of matrix (10 cycles).

Plate Spotting and Data Acquisition:

1 µl of desalted eluate was spotted on the MALDI target and allowed to air dry. 1 µl of calibrant was spotted, allowed to dry and then overlaid with 1 µl of α-Cyano-4-hydroxycinnamic acid (HCCA, 10 mg/ml in 50% ACN, 2.5% TFA). Molecular weight measurement was performed on a Microflex-TOF mass spectrometer (Bruker Daltonics, Billerica, MA) in positive ion, linear mode using an ion source voltage of 20 kV. External calibration was performed using a protein calibration mixture (Protein Standard I, Bruker Daltonics) on a spot adjacent to the sample. The raw data was then processed in the FlexAnalysis software (version 3.4, Bruker Daltonics).

Utilizing
high laser power combined with mis-matched matrix and molecule type appears to have introduced a systematic mass error of ~6 Da.

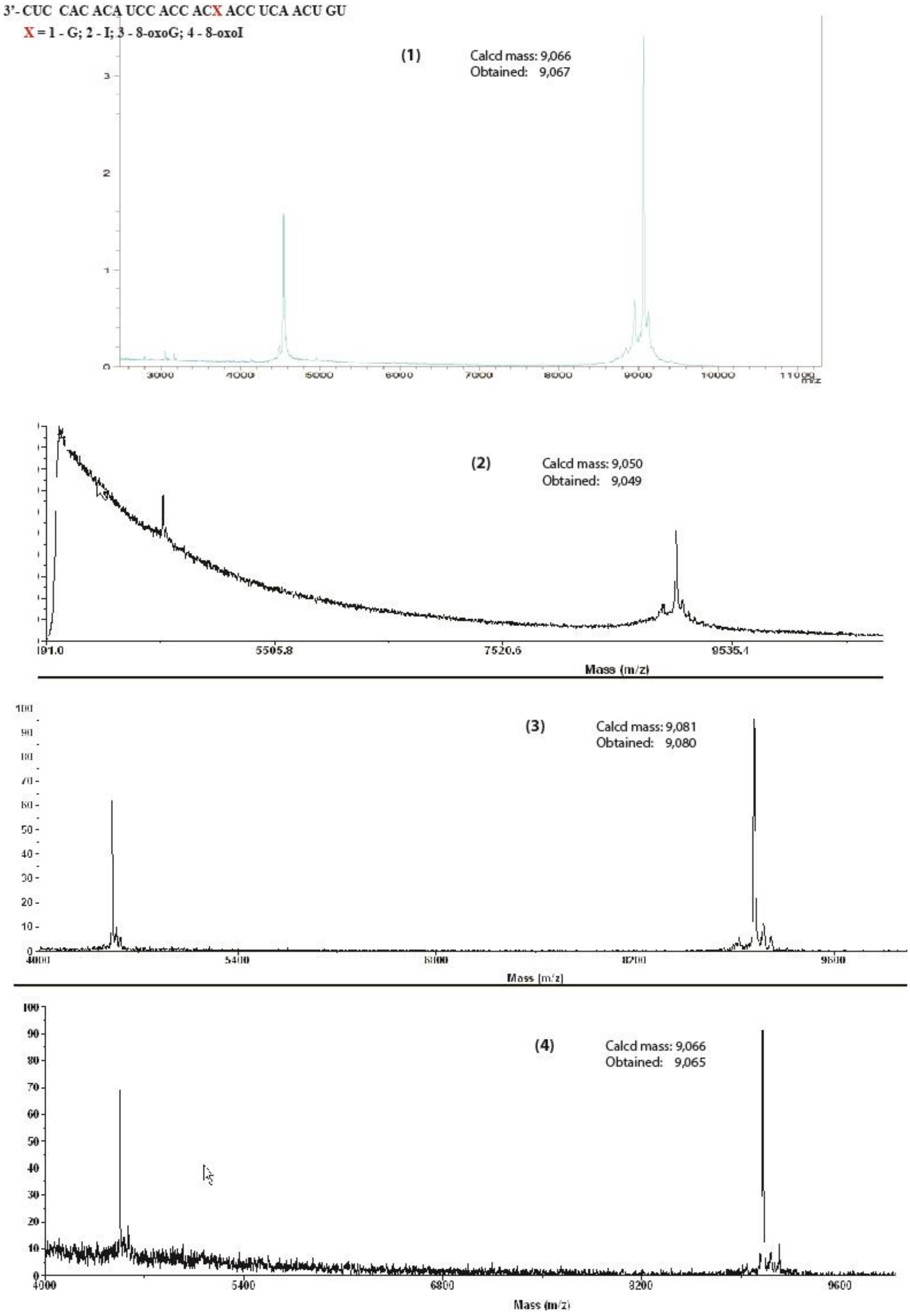

MALDI-TOF of oligonucleotides **1 - 4**

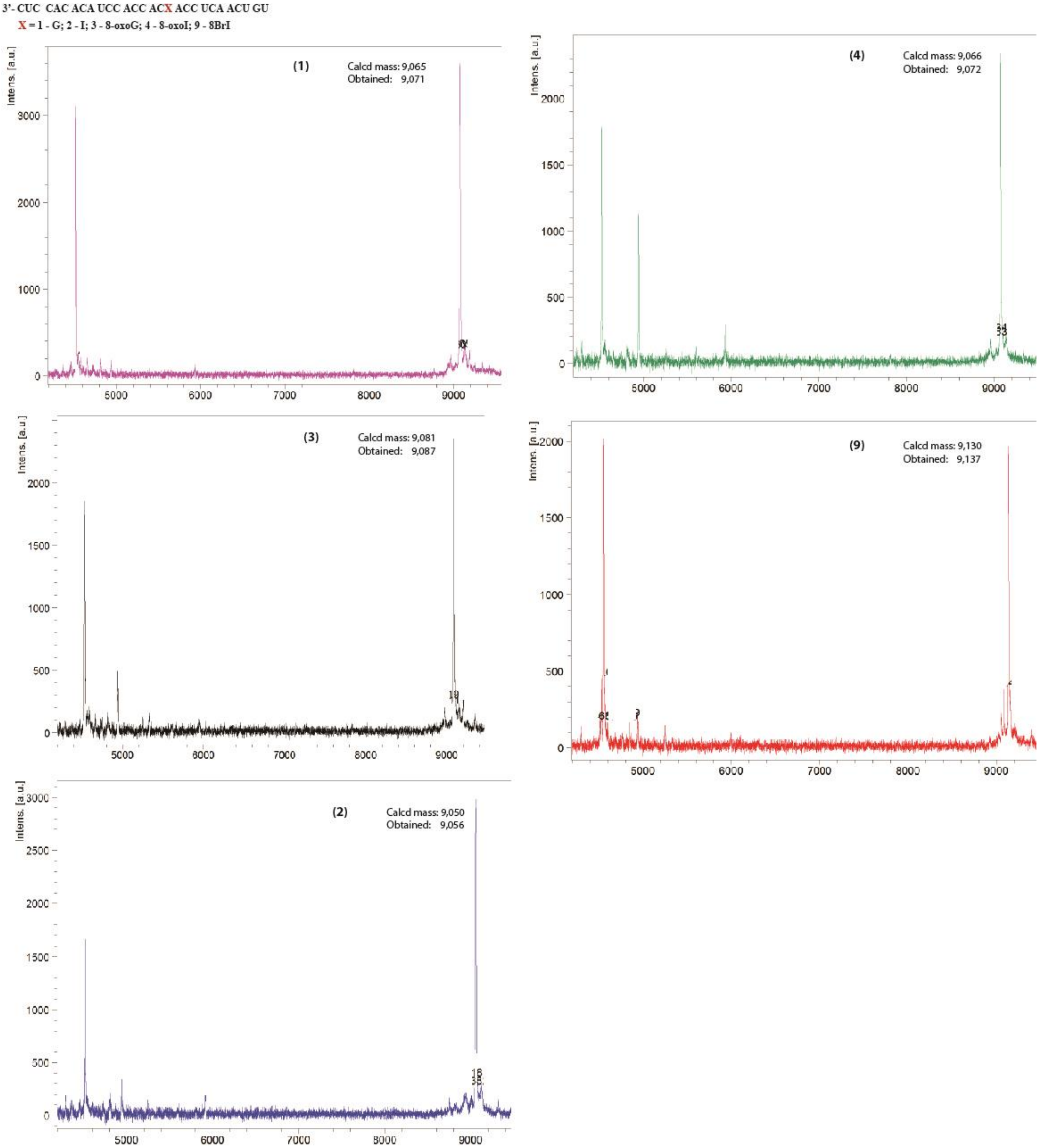

MALDI-TOF of oligonucleotides **1** – **4** **&** **9**
