# Supplementary material for: Translesion synthesis by AMV, HIV, and MMLVreverse transcriptases using RNA templates containing inosine, guanosine, and their 8-oxo-7,8-dihydropurine derivatives
Source: PLoS One. 2020 Aug 28;15(8):e0235102. doi: 10.1371/journal.pone.0235102 (PMC7455023; doi:10.1371/journal.pone.0235102)
Supplement: S4 File — (PDF) [file pone.0235102.s004.pdf]

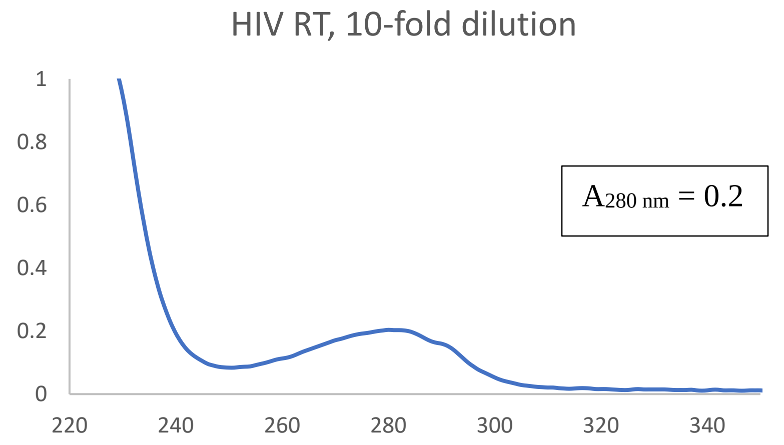

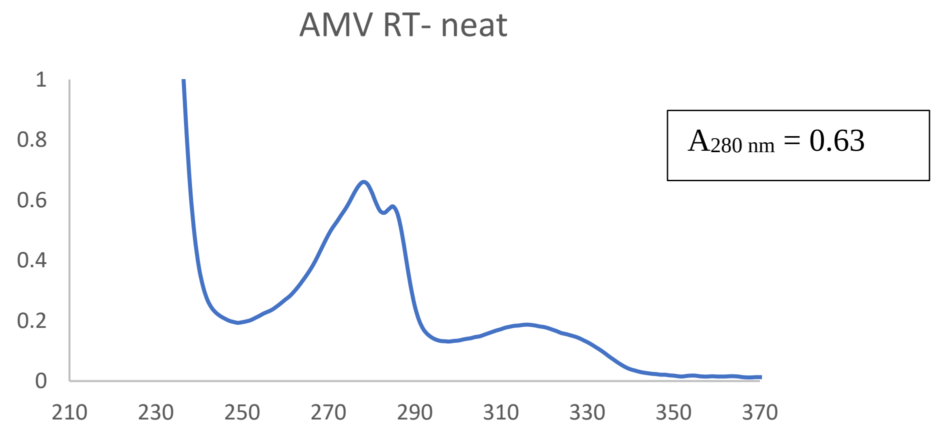

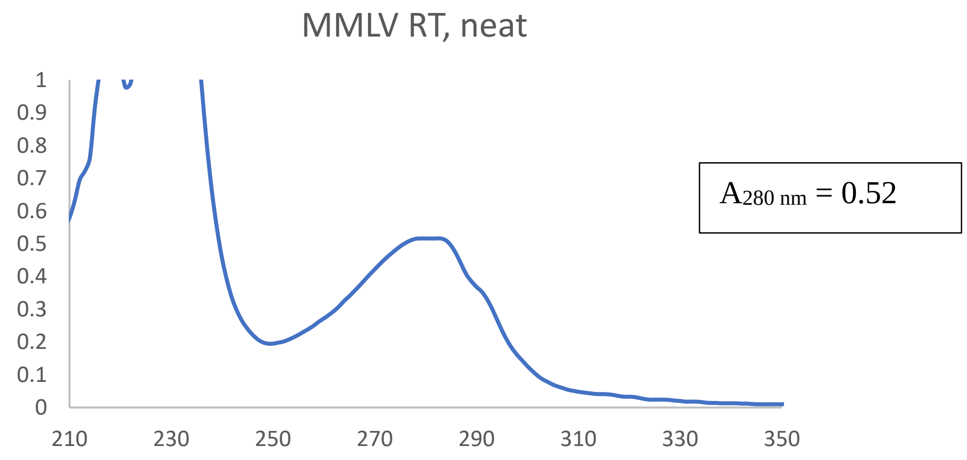

S4 File. UV-vis spectra of HIV, MMLV, and AMV RTs.
Conditions: HIV RT - 0.5 mM Tris-acetate, 0.75 mM Potassium Acetate, 0.08 mM Magnesium Acetate, 0.01 mM DTT (pH 8.3); AMV RT - 5 mM Tris-acetate, 7.5 mM Potassium Acetate, 0.8 mM Magnesium Acetate, 1 mM DTT (pH 8.3)*; MMLV RT - 5 mM Tris-HCl, 7.5 mM KCl, 0.3 mM $MgCl_2$, 1 mM DTT (pH 8.3).
* Absorbance at 320 nm can be attributed to scattering.[5]

(5) McGaughey, R. W.; Murray, F. A. Properties of blastokinin: Amino acid composition, evidence for subunits, and estimation of isoelectric point.
