# Supplementary material for: Translesion synthesis by AMV, HIV, and MMLVreverse transcriptases using RNA templates containing inosine, guanosine, and their 8-oxo-7,8-dihydropurine derivatives
Source: PLoS One. 2020 Aug 28;15(8):e0235102. doi: 10.1371/journal.pone.0235102 (PMC7455023; doi:10.1371/journal.pone.0235102)
Supplement: S5 File — Typical CD of RNA/DNA duplex at 20°C and 85° C; CD and Tm measurement for duplex 1:5; CD and Tm measurement for duplexes 1:5–4:5; 1:6–4:6; and 9:5, 9:6; CD and Tm measurement for duplexes 1:7–4:7; 1:10–4:10; and 9:7, 9:10; CD and Tm measurement for duplexes 1:11–4:11; 9:11; 1:8–4:8; and 9:8; Tm measurements and ANOVA for duplexes 1:5, 1:6. 1:7, 1:8, 1:10, 1:11; Tm measurements and ANOVA for duplexes 2:5, 2:6. 2:7, 2:8, 2:10, 2:11; Tm measurements and ANOVA for duplexes 3:5, 3:6. 3:7, 3:8, 3:10, 3:11; Tm measurements and ANOVA for duplexes 4:5, 4:6. 4:7, 4:8, 4:10, 4:11; and Tm measurements and ANOVA for duplexes 9:5, 9:6. 9:7, 9:8, 9:10, 9:11. (PDF) [file pone.0235102.s005.pdf]

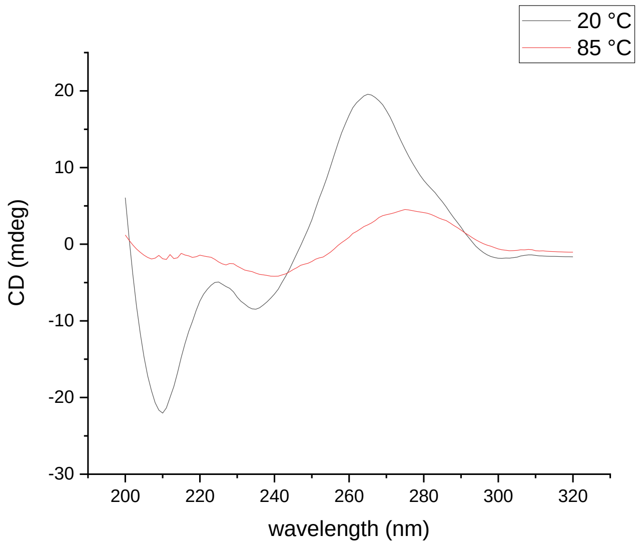

Typical CD spectrum of annealed duplex at 20 °C and denatured duplex at 85 °C

Experimental conditions: 1.5 µM RNA and 2.0 µM complement DNA prepared in PBS (10 mM NaCl, 5 mM $MgCl_2$, 1 mM Na_2HPO_4 pH 7.2)

1 - 4 & 9   3'- CUC CAC ACA UCC ACC ACX ACC UCA ACU GU   X = 1 - G; 2 - I; 3 - 8-oxoG; 4 - 8-oxoI; 9 - 8-BrI

5   5' - HOP*(O)3 - GAG GTG TGT AGG TGG TG

3'- CUC CAC ACA UCC ACC ACX ACC UCA ACU GU

5' - HOP*(O)3 - GAG GTG TGT AGG TGG TGY   Y = 6 - dA; 7 - dC; 8 - dT

3'- CUC CAC ACA UCC ACC ACX ACC UCA ACU GU

5' - HOP*(O)3 - GAG GTG TGT AGG TGG TGY U   Y = 10 - dA; 11 - dC

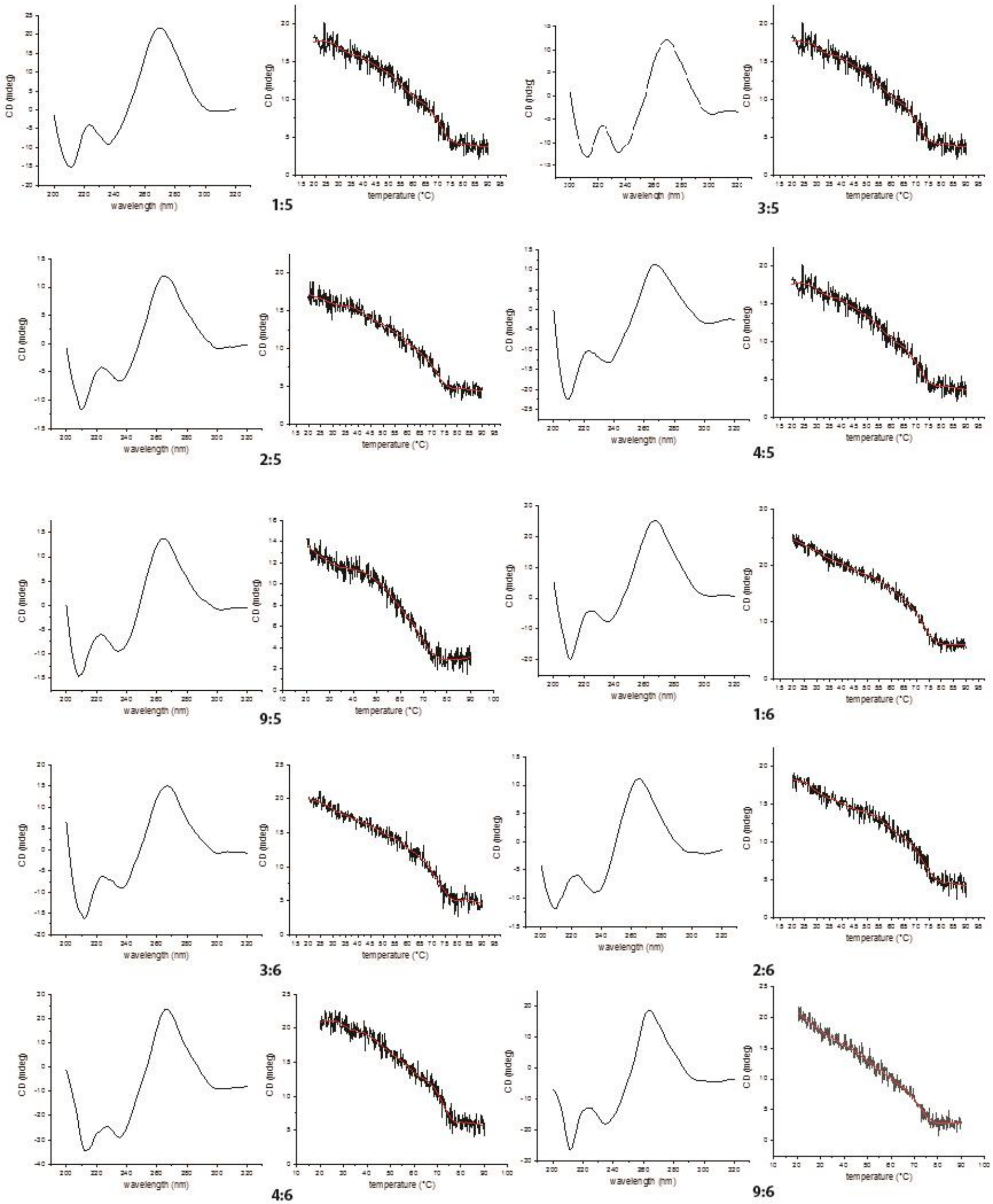

CD and T_m measurement for duplexes **1**:**5 - 4**:**5; 1**:**6-4:6;** and **9:5, 9:6**.

1 - 4 & 9 3'- CUC CAC ACA UCC ACC ACX ACC UCA ACU GU **X = 1 - G; 2 - I; 3 - 8-oxoG; 4 - 8-oxoI; 9 - 8-BrI**

5 5' - HOP*(O)3 - GAG GTG TGT AGG TGG TG

3'- CUC CAC ACA UCC ACC ACX ACC UCA ACU GU

5' - HOP*(O)3 - GAG GTG TGT AGG TGG TGY **Y = 6 - dA; 7 - dC; 8 - dT**

3'- CUC CAC ACA UCC ACC ACX ACC UCA ACU GU

5' - HOP*(O)3 - GAG GTG TGT AGG TGG TGY U **Y = 10 - dA; 11 - dC**

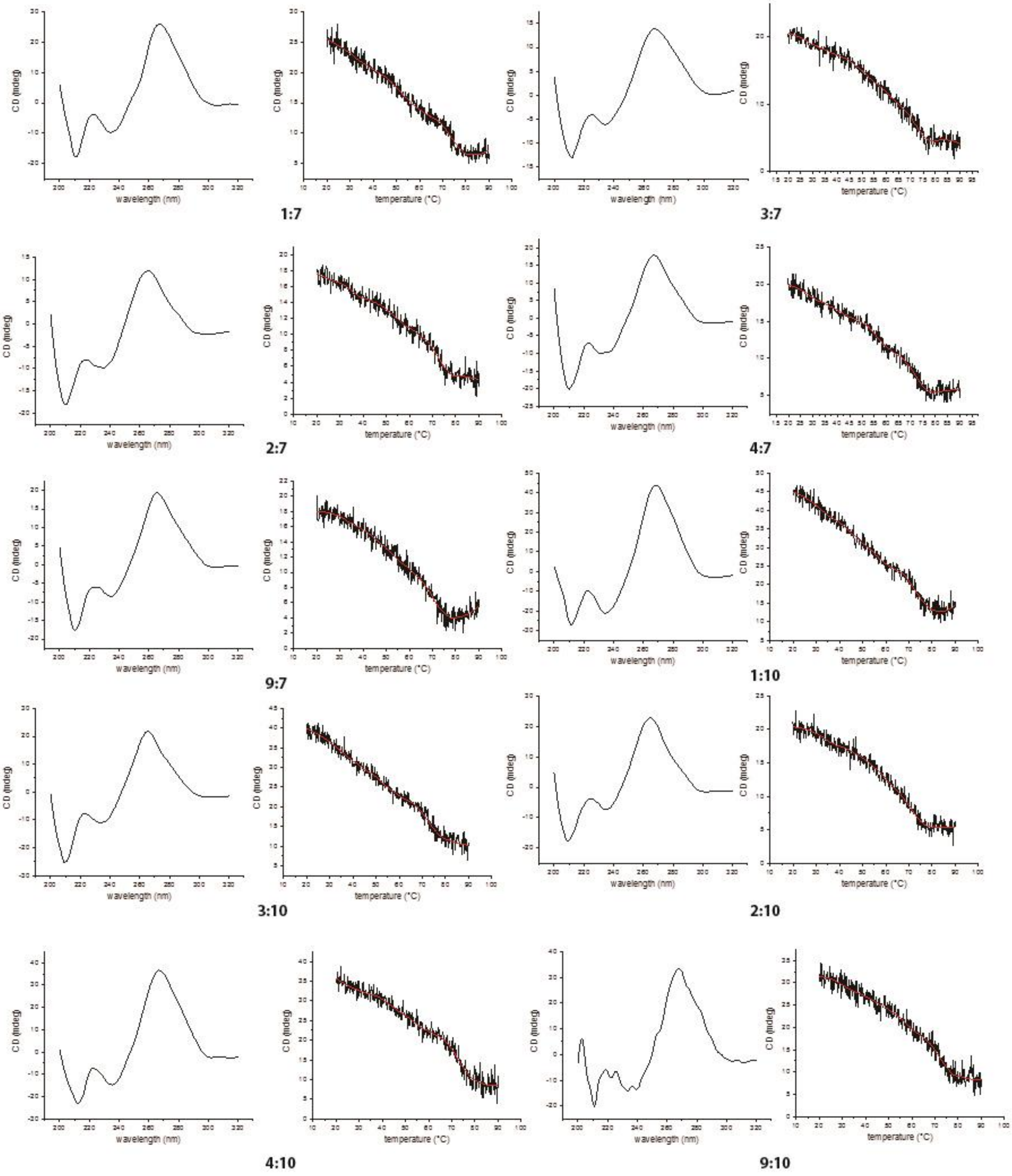

CD and T_m measurement for duplexes **1**:**7** - **4**:**7**; **1**:**10**-**4**:**10**; and **9**:**7**, **9**:**10**.

1 - 4 & 9 3'- CUC CAC ACA UCC ACC ACX ACC UCA ACU GU **X = 1 - G; 2 - I; 3 - 8-oxoG; 4 - 8-oxoI; 9 - 8-BrI**
5 5' - HOP*(O)3 - GAG GTG TGT AGG TGG TG

3'- CUC CAC ACA UCC ACC ACX ACC UCA ACU GU
5' - HOP*(O)3 - GAG GTG TGT AGG TGG TGY **Y = 6 - dA; 7 - dC; 8 - dT**

3'- CUC CAC ACA UCC ACC ACX ACC UCA ACU GU
5' - HOP*(O)3 - GAG GTG TGT AGG TGG TGY U **Y = 10 - dA; 11 - dC**

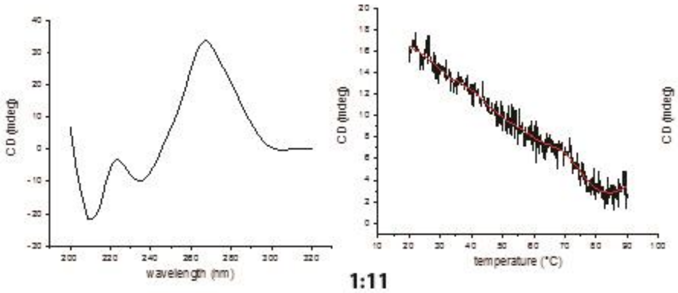

1:11

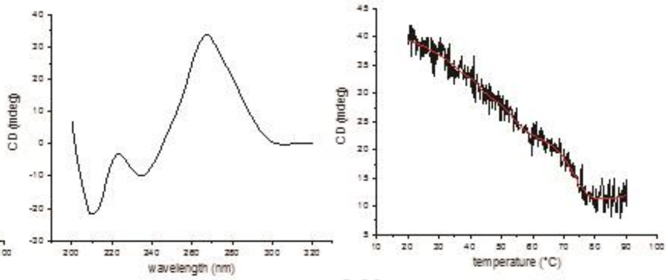

3:11

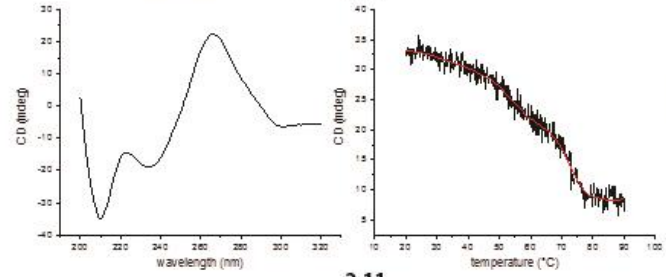

2:11

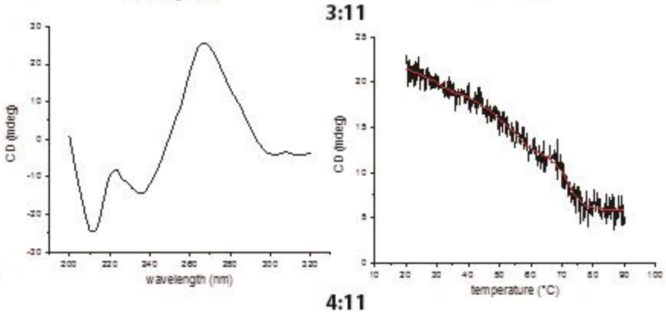

4:11

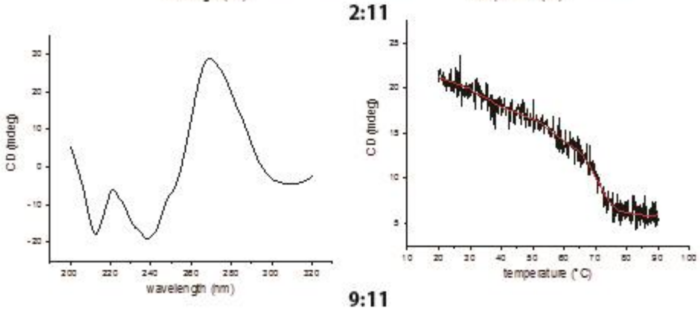

9:11

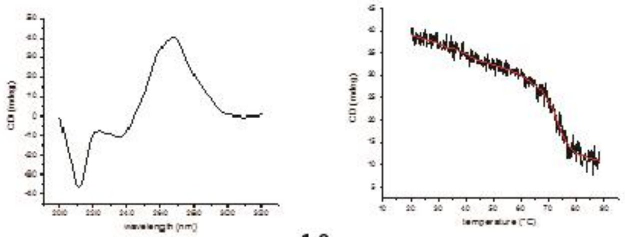
1:8

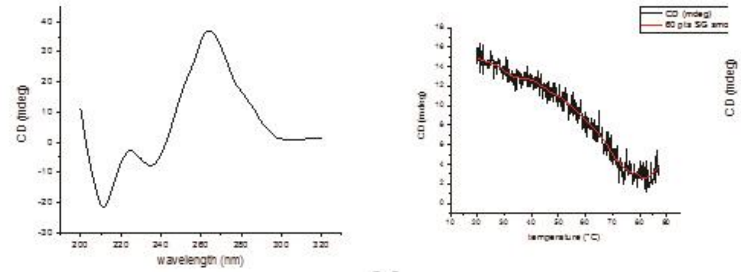

2:8

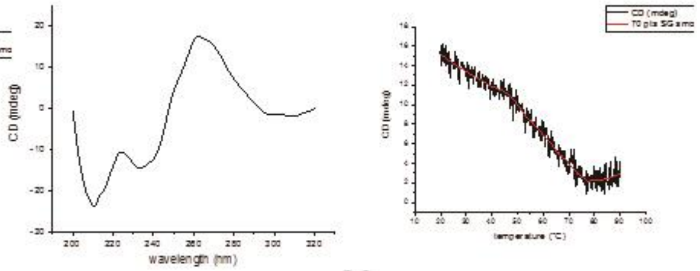

3:8

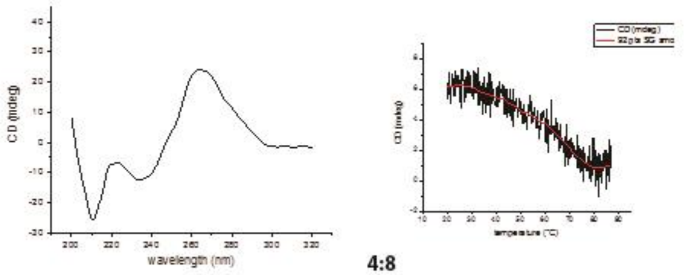

4:8

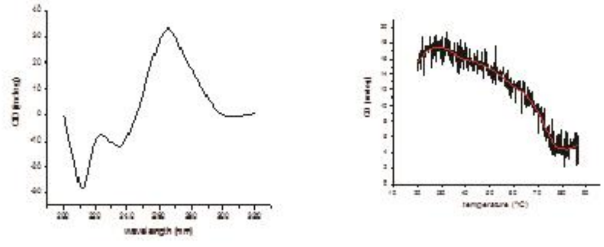
9:8

CD and T_m measurement for duplexes **1:11 - 4:11; 9:11**; **1:8 - 4:8;** and **9:8**

5'-CUC-CAC-ACA-UCC-ACC-AC**G**-ACC-UCA-ACU-CU-3'							**Duplex**
	1	2	3	avg	std dev	RSD	
primer1	69.6	70.4	70.0	70.0	0.4	0.6%	1:5
extra A	71.4	72.2	72.0	71.9	0.4	0.6%	1:6
extra C	74.2	72.5	73.4	73.4	0.8	1.1%	1:7
extra T	72.5	71.8	72.6	72.3	0.4	0.6%	1:8
extra AU	72.8	73.4	73.6	73.3	0.4	0.6%	1:10
extra CU	74.4	75.2	74.4	74.7	0.5	0.6%	1:11

Anova: Single Factor

SUMMARY

Groups	*Count*	*Sum*	*Average*	*Variance*	**Duplex**
primer1	4	280	70	0.106667	1:5
extra A	4	287.4	71.85	0.111667	1:6
extra C	4	293.4	73.35	0.455	1:7
extra T	4	289.1333	72.28333	0.117222	1:8
extra AU	4	293.0667	73.26667	0.115556	1:10
extra CU	4	298.6667	74.66667	0.142222	1:11

ANOVA

Source of Variation	*SS*	*df*	*MS*	*F*	*P-value*	*F crit*
Between Groups	50.78093	5	10.15619	58.12761	1.78E-10	2.772853
Within Groups	3.145	18	0.174722			
Total	53.92593	23				

Tables containing the values corresponding to T_m measurements carried out in triplicate. t1, t2, t3 stand for trials 1, 2, and 3; along with analysis of variance (ANOVA) for duplexes **1:5, 1:6. 1:7, 1:8, 1:10, 1:11.**

5'-CUC-CAC-ACA-UCC-ACC-AC**I**-ACC-UCA-ACU-CU-3'							**Duplex**
	1	2	3	avg	std dev	RSD	
primer1	70.0	71.0	70.4	70.5	0.5	0.7%	2:5
extra A	72.2	72.0	71.8	72.0	0.2	0.3%	2:6
extra C	71.2	72.0	71.8	71.7	0.4	0.6%	2:7
extra T	72.8	72.0	71.8	72.2	0.5	0.7%	2:8
extra AU	71.6	72.0	72.3	72.0	0.4	0.5%	2:10
extra CU	72.6	73.2	73.2	73.0	0.3	0.5%	2:11

Anova: Single Factor

SUMMARY					**Duplex**
Groups	*Count*	*Sum*	*Average*	*Variance*	
primer1	4	281.8667	70.46667	0.168889	2:5
extra A	4	288	72	0.026667	2:6
extra C	4	286.6667	71.66667	0.115556	2:7
extra T	4	288.8	72.2	0.186667	2:8
extra AU	4	287.8667	71.96667	0.082222	2:10
extra CU	4	292	73	0.08	2:11

ANOVA

Source of Variation	*SS*	*df*	*MS*	*F*	*P-value*	*F crit*
Between Groups	13.68667	5	2.737333	24.88485	1.69E-07	2.772853
Within Groups	1.98	18	0.11			
Total	15.66667	23				

Tables containing the values corresponding to T_m measurements carried out in triplicate. t1, t2, t3 stand for trials 1, 2, and 3; along with analysis of variance (ANOVA) for duplexes **2:5, 2:6. 2:7, 2:8, 2:10, 2:11.**

5'-CUC-CAC-ACA-UCC-ACC-AC**G**^{oxo}-ACC-UCA-ACU-CU-3'							**Duplex**
	1	2	3	avg	std dev	RSD	
primer1	70.6	70.6	70.0	70.4	0.3	0.5%	3:5
extra A	72.4	72.8	71.6	72.3	0.6	0.8%	3:6
extra C	71.1	70.0	70.6	70.6	0.6	0.8%	3:7
extra T	71.8	71.0	72.2	71.7	0.6	0.9%	3:8
extra AU	71.8	71.4	71.3	71.5	0.3	0.4%	3:10
extra CU	71.8	72.4	71.8	72.0	0.3	0.5%	3:11

Anova: Single Factor

SUMMARY

Groups	*Count*	*Sum*	*Average*	*Variance*	**Duplex**
primer1	4	281.6	70.4	0.08	3:5
extra A	4	289.0667	72.26667	0.248889	3:6
extra C	4	282.2667	70.56667	0.202222	3:7
extra T	4	286.6667	71.66667	0.248889	3:8
extra AU	4	286	71.5	0.046667	3:10
extra CU	4	288	72	0.08	3:11

ANOVA

Source of Variation	*SS*	*df*	*MS*	*F*	*P-value*	*F crit*
Between Groups	11.54667	5	2.309333	15.28235	6.19E-06	2.772853
Within Groups	2.72	18	0.151111			
Total	14.26667	23				

Tables containing the values corresponding to T_m measurements carried out in triplicate. t1, t2, t3 stand for trials 1, 2, and 3; along with analysis of variance (ANOVA) for duplexes **3:5, 3:6. 3:7, 3:8, 3:10, 3:11.**

5'-CUC-CAC-ACA-UCC-ACC-ACI^{oxo}-ACC-UCA-ACU-CU-3'							Duplex
	1	2	3	avg	std dev	RSD	
primer1	70.6	70.2	69.8	70.2	0.4	0.6%	4:5
extra A	71.0	70.8	71.6	71.1	0.4	0.6%	4:6
extra C	71.2	70.6	70.6	70.8	0.3	0.5%	4:7
extra T	70.8	71.8	72.0	71.5	0.6	0.9%	4:8
extra AU	71.8	72.8	71.7	72.1	0.6	0.8%	4:10
extra CU	71.8	71.0	72.4	71.7	0.7	1.0%	4:11

Anova: Single Factor

SUMMARY

Groups	*Count*	*Sum*	*Average*	*Variance*	**Duplex**
primer1	4	280.8	70.2	0.1066667	4:5
extra A	4	284.5333	71.13333	0.1155556	4:6
extra C	4	283.2	70.8	0.08	4:7
extra T	4	286.1333	71.53333	0.2755556	4:8
extra AU	4	288.4	72.1	0.2466667	4:10
extra CU	4	286.9333	71.73333	0.3288889	4:11

ANOVA

Source of Variation	*SS*	*df*	*MS*	*F*	*P-value*	*F crit*
Between Groups	9.42	5	1.884	9.8011561	0.000119	2.772853
Within Groups	3.46	18	0.192222			
Total	12.88	23				

Tables containing the values corresponding to T_m measurements carried out in triplicate. t1, t2, t3 stand for trials 1, 2, and 3; along with analysis of variance (ANOVA) for duplexes **4:5, 4:6. 4:7, 4:8, 4:10, 4:11.**

5'-CUC-CAC-ACA-UCC-ACC-ACI^{Br}-ACC-UCA-ACU-CU-3'							Duplex
	1	2	3	avg	std dev	RSD	
primer1	67.8	68.8	67.8	68.1	0.6	0.8%	9:5
extra A	71.6	71.2	70.4	71.1	0.6	0.9%	9:6
extra C	70.8	70.4	70.4	70.5	0.2	0.3%	9:7
extra T	72.0	72.2	72.2	72.1	0.1	0.2%	9:8
extra AU	71.2	71.8	72.2	71.7	0.5	0.7%	9:10
extra CU	70.4	70.6	70.2	70.4	0.2	0.3%	9:11

Anova: Single Factor

SUMMARY

Groups	*Count*	*Sum*	*Average*	*Variance*	**Duplex**
primer1	4	272.5333	68.13333	0.222222	9:5
extra A	4	284.2667	71.06667	0.248889	9:6
extra C	4	282.1333	70.53333	0.035556	9:7
extra T	4	288.5333	72.13333	0.008889	9:8
extra AU	4	286.9333	71.73333	0.168889	9:10
extra CU	4	281.6	70.4	0.026667	9:11

ANOVA

Source of Variation	*SS*	*df*	*MS*	*F*	*P-value*	*F crit*
Between Groups	39.82222	5	7.964444	67.2	5.224E-11	2.772853
Within Groups	2.133333	18	0.118519			
Total	41.95556	23				

Tables containing the values corresponding to T_m measurements carried out in triplicate. t1, t2, t3 stand for trials 1, 2, and 3; along with analysis of variance (ANOVA) for duplexes **9:5, 9:6. 9:7, 9:8, 9:10, 9:11.**
