# Supplementary material for: Translesion synthesis by AMV, HIV, and MMLVreverse transcriptases using RNA templates containing inosine, guanosine, and their 8-oxo-7,8-dihydropurine derivatives
Source: PLoS One. 2020 Aug 28;15(8):e0235102. doi: 10.1371/journal.pone.0235102 (PMC7455023; doi:10.1371/journal.pone.0235102)
Supplement: S6 File — (PDF) [file pone.0235102.s006.pdf]

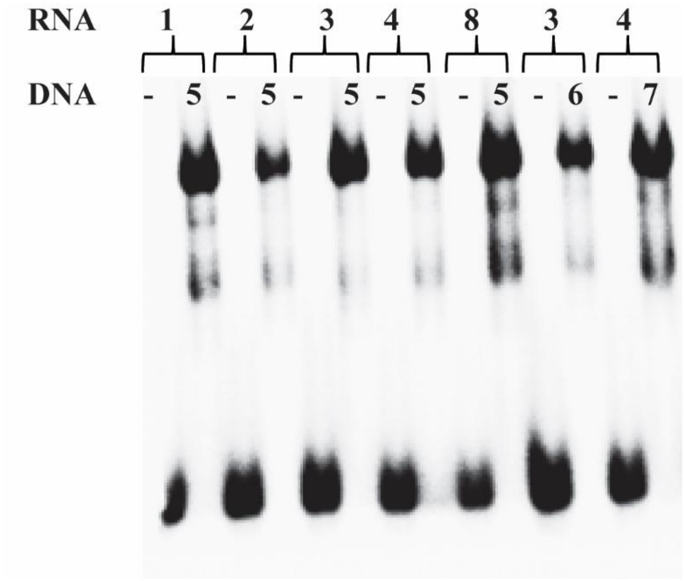

S6 File: Native PAGE (20 %) of RNA w/wo DNA, showing duplex formation in the MMLV buffer (as described in the experimental section).
