# Supplementary material for: Translesion synthesis by AMV, HIV, and MMLVreverse transcriptases using RNA templates containing inosine, guanosine, and their 8-oxo-7,8-dihydropurine derivatives
Source: PLoS One. 2020 Aug 28;15(8):e0235102. doi: 10.1371/journal.pone.0235102 (PMC7455023; doi:10.1371/journal.pone.0235102)
Supplement: S7 File — Reactions carried out at rt. (PDF) [file pone.0235102.s007.pdf]

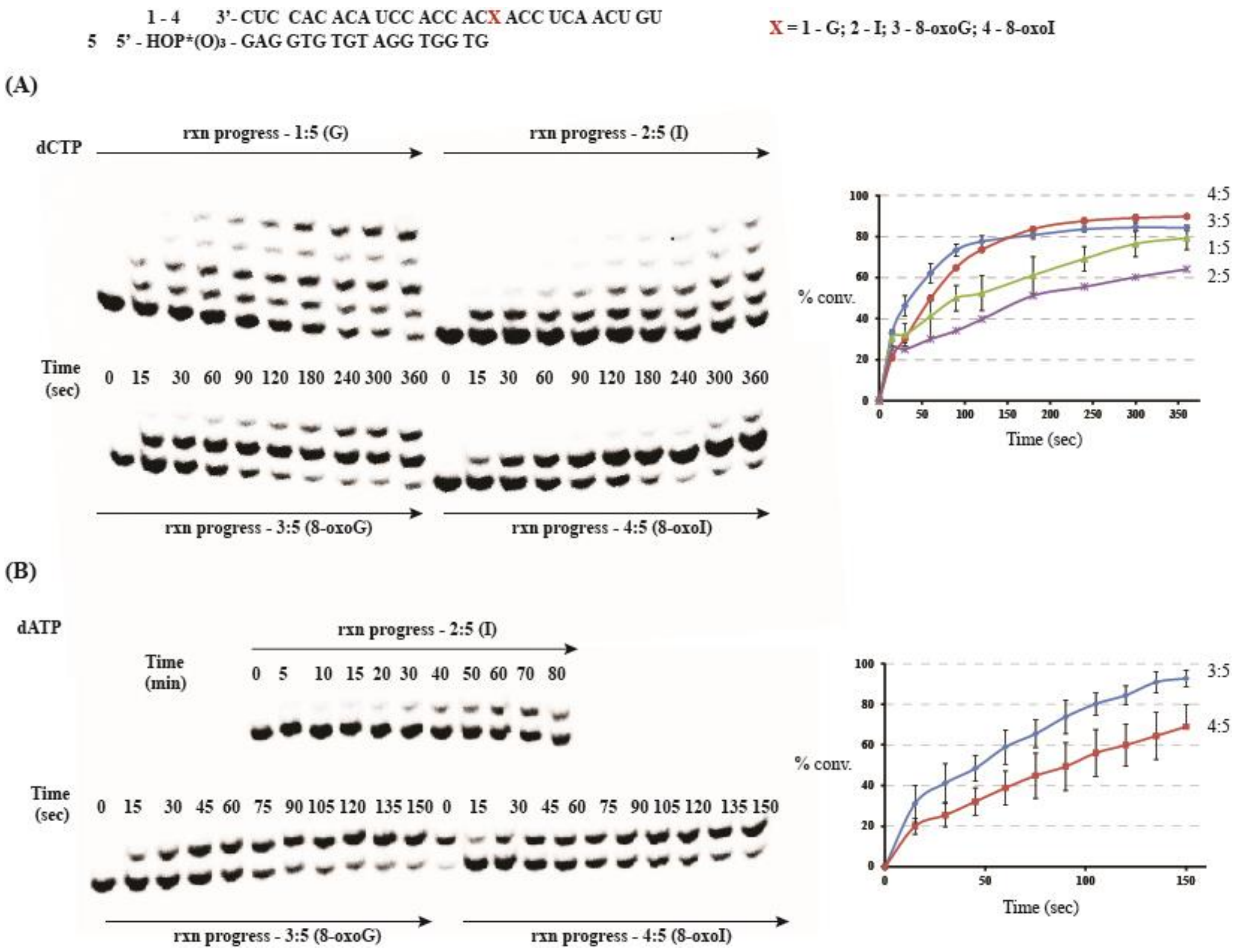

S7 File: Relative rates for **1**:**5** – **4**:**5** with dCTP and **2**:**5** – **4**:**5** with dATP at constant [dNTP] and [AMV-RT] as a function of time. Reactions carried out at rt.
