# Supplementary figures and images for: Translesion synthesis by AMV, HIV, and MMLVreverse transcriptases using RNA templates containing inosine, guanosine, and their 8-oxo-7,8-dihydropurine derivatives
Source: PLoS One. 2020 Aug 28;15(8):e0235102. doi: 10.1371/journal.pone.0235102 (PMC7455023; doi:10.1371/journal.pone.0235102)

**Y = 6 - A; 7 - C**

**Y =**

|                                                                                   | A |   |   |   |   |   | C |   |   |   |   |   |
|-----------------------------------------------------------------------------------|---|---|---|---|---|---|---|---|---|---|---|---|
| dNTP                                                                              | - | G | C | T | A | M | - | G | C | T | A | M |
| 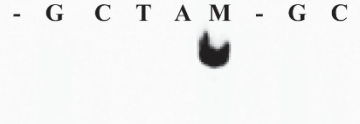 |   |   |   |   |   |   |   |   |   |   |   |   |

**S8 File.** Duplexes **9:6** and **9:7** in the presence of AMV-RT.

Supplement: S8 File — (PDF) [file pone.0235102.s008.pdf]

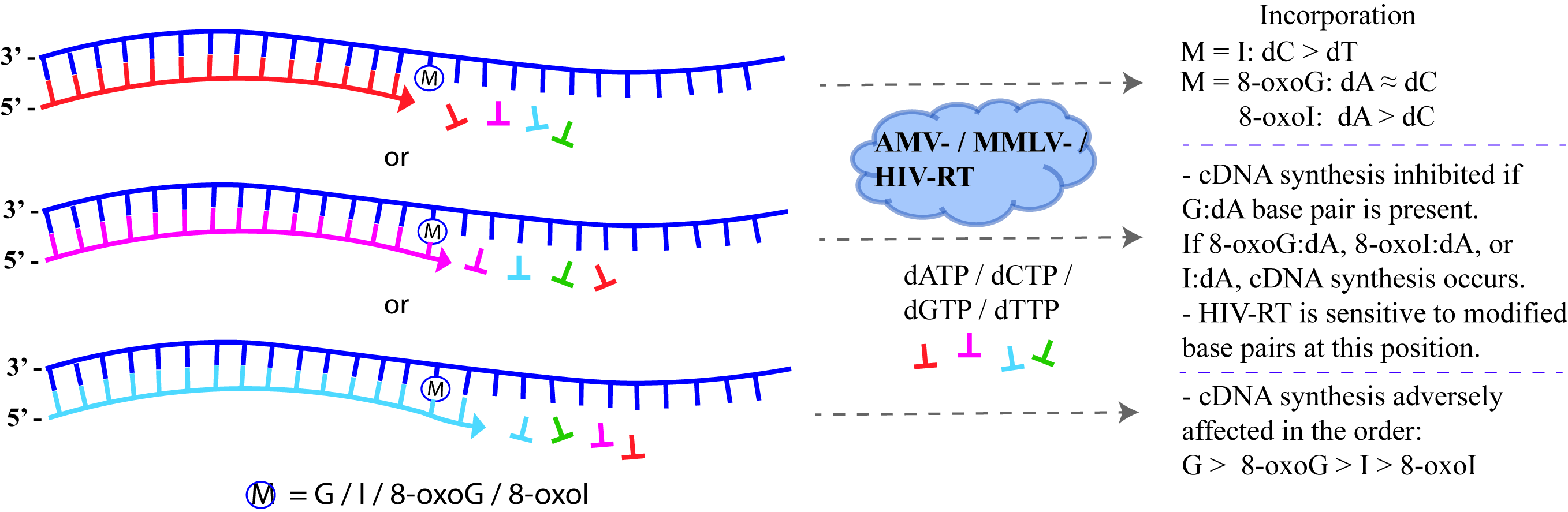

Supplement: S1 Fig — (TIF) [file pone.0235102.s018.tif]
