# Supplementary material for: Translesion synthesis by AMV, HIV, and MMLVreverse transcriptases using RNA templates containing inosine, guanosine, and their 8-oxo-7,8-dihydropurine derivatives
Source: PLoS One. 2020 Aug 28;15(8):e0235102. doi: 10.1371/journal.pone.0235102 (PMC7455023; doi:10.1371/journal.pone.0235102)
Supplement: S10 File — Top three experiments represent incubation times of 5 min and the bottom experiment represents incubation time of 40 min. (PDF) [file pone.0235102.s010.pdf]

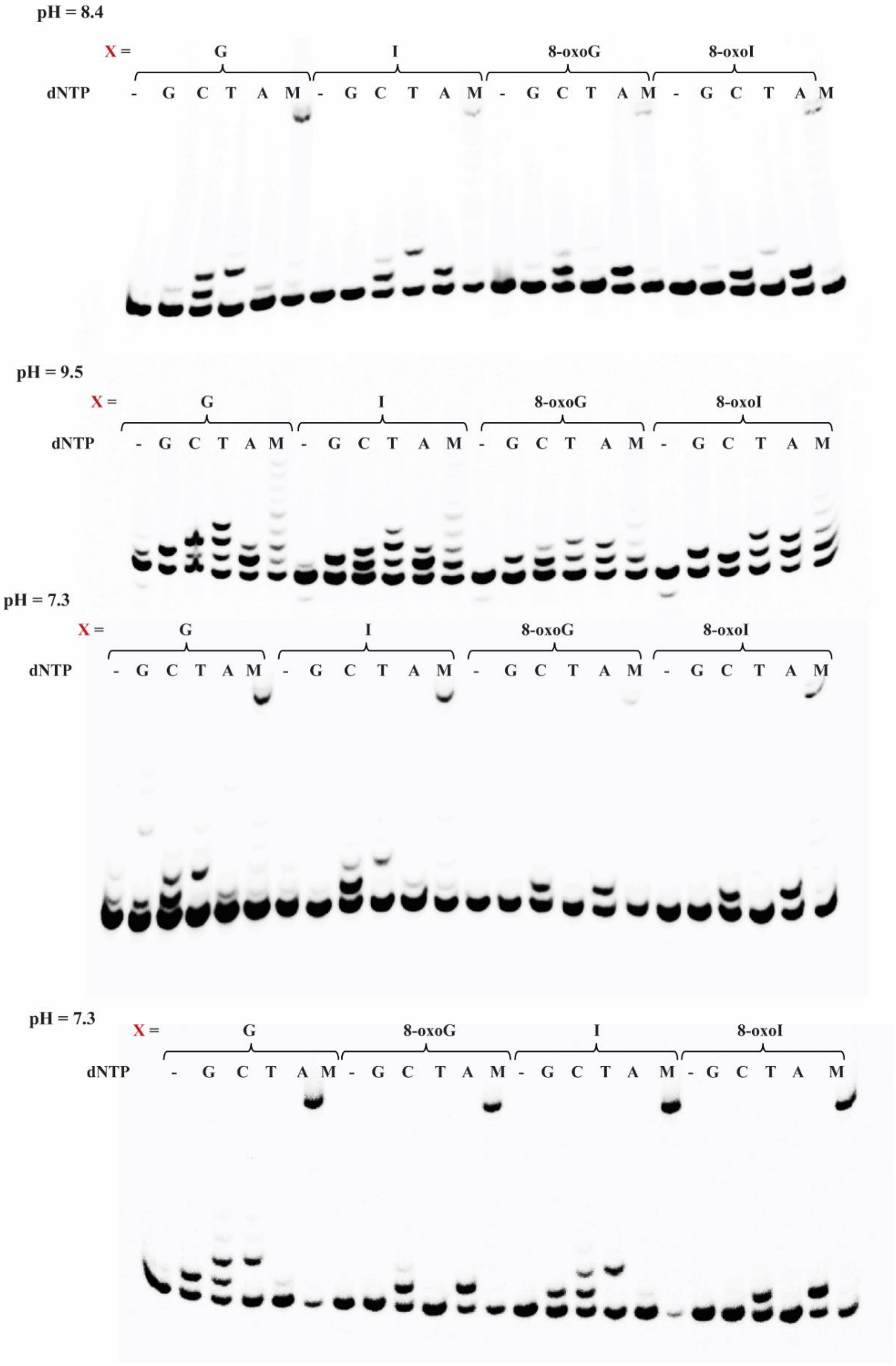

S10 File. Duplexes **1**:**5** - **4**:**5** in the presence of AMV-RT at various pH values. Top three experiments represent incubation times of 5 min and the bottom experiment represents incubation time of 40 min
