# Supplementary material for: Translesion synthesis by AMV, HIV, and MMLVreverse transcriptases using RNA templates containing inosine, guanosine, and their 8-oxo-7,8-dihydropurine derivatives
Source: PLoS One. 2020 Aug 28;15(8):e0235102. doi: 10.1371/journal.pone.0235102 (PMC7455023; doi:10.1371/journal.pone.0235102)
Supplement: S11 File — RNA:DNA 1:5–4:5 at higher (top) and lower (bottom) [MMLV-RT], see table in experimental section for description; and RNA:DNA 1:6–4:6 & 1:7–4:7 at lower [MMLV]. (PDF) [file pone.0235102.s011.pdf]

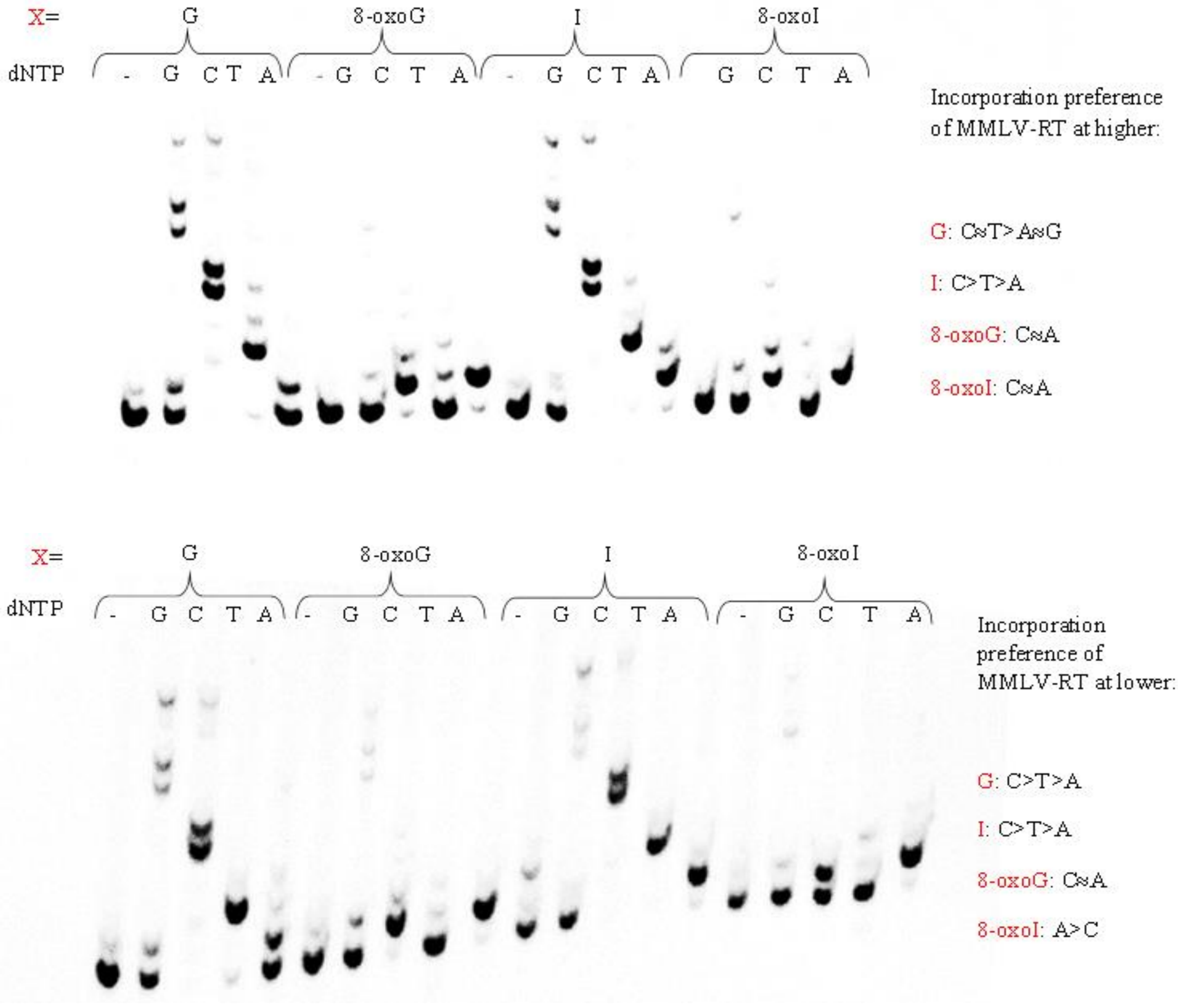

S11 File. RNA:DNA **1**:**5**-**4**:**5** at higher (top) and lower (bottom) [MMLV-RT], see table in experimental section for description.

1-4 3’-CUC CAC ACA UCC ACC ACX ACC UCA ACU GU

5’-HOP*$(O)_3$- GAG GTG TGT AGG TGG TGY

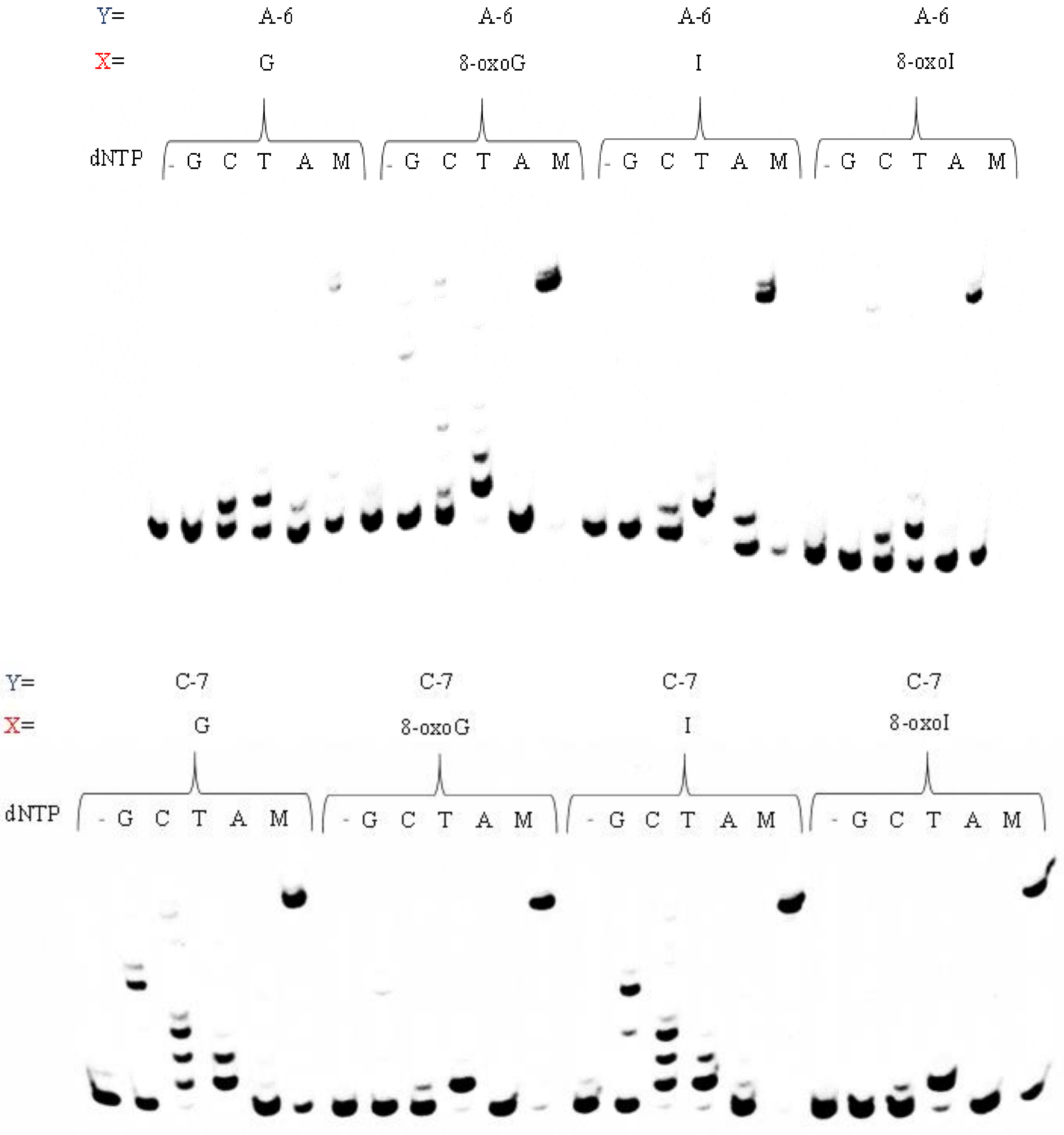

S11 File. RNA:DNA **1**:**6**-**4**:**6** & **1**:**7**-**4**:**7** at lower [MMLV].
