# Supplementary material for: Translesion synthesis by AMV, HIV, and MMLVreverse transcriptases using RNA templates containing inosine, guanosine, and their 8-oxo-7,8-dihydropurine derivatives
Source: PLoS One. 2020 Aug 28;15(8):e0235102. doi: 10.1371/journal.pone.0235102 (PMC7455023; doi:10.1371/journal.pone.0235102)
Supplement: S12 File — (PDF) [file pone.0235102.s012.pdf]

1-4 3’-CUC CAC ACA UCC ACC ACX ACC UCA ACU GU

5 5’-HOP*(O)$_3$- GAG GTG TGT AGG TGG TG

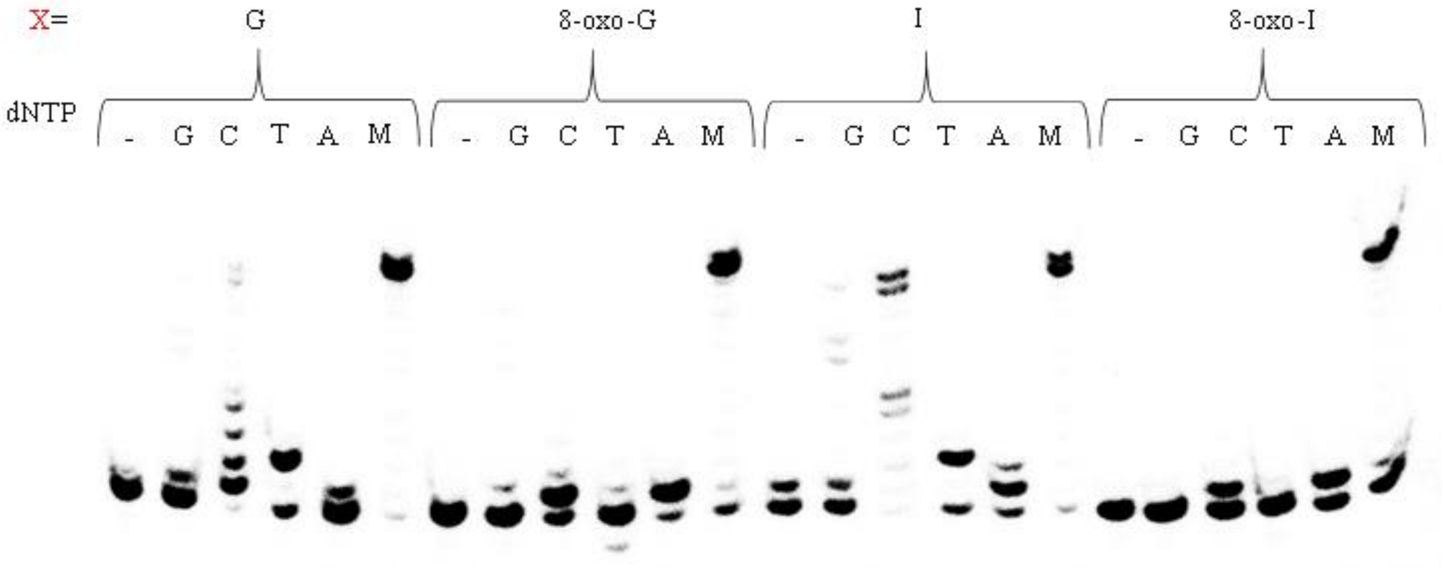

S12 File. RNA:DNA **1**:**5**-**4**:**5** using SSII (Superscript II).
