# Supplementary material for: Translesion synthesis by AMV, HIV, and MMLVreverse transcriptases using RNA templates containing inosine, guanosine, and their 8-oxo-7,8-dihydropurine derivatives
Source: PLoS One. 2020 Aug 28;15(8):e0235102. doi: 10.1371/journal.pone.0235102 (PMC7455023; doi:10.1371/journal.pone.0235102)
Supplement: S13 File — HIV units used were ca. 0.187 per well, 30 times more than the experiments described on the manuscript. (PDF) [file pone.0235102.s013.pdf]

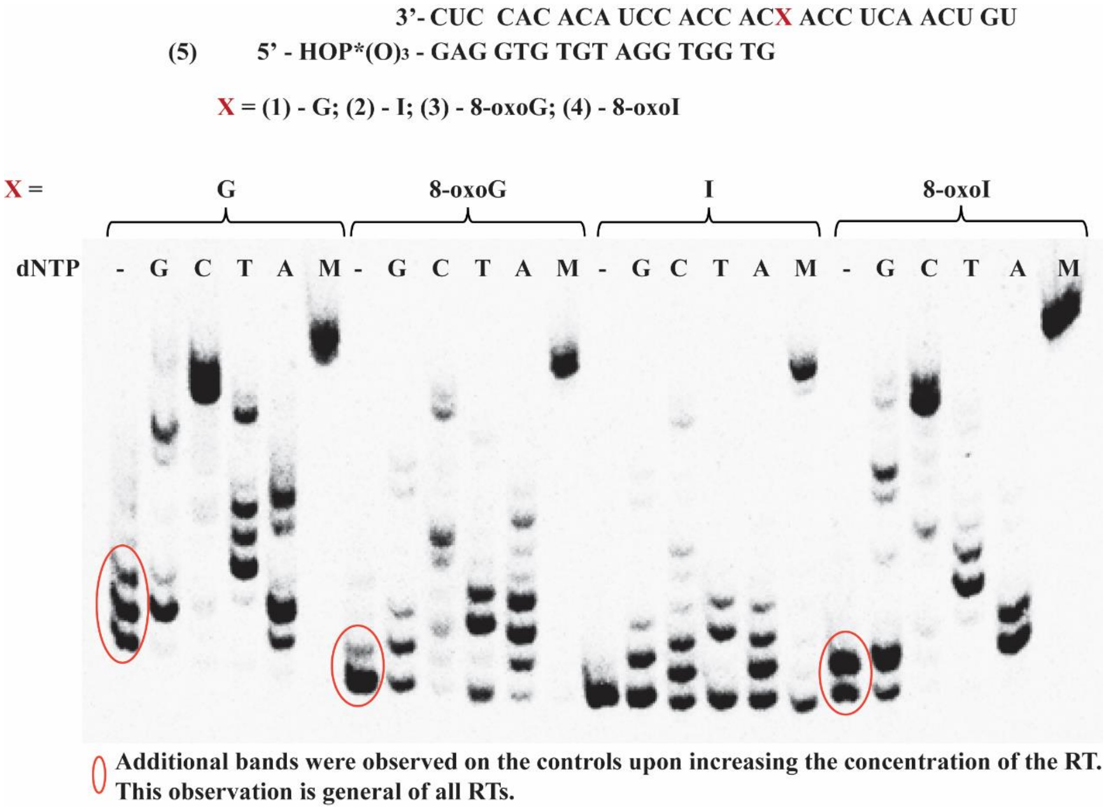

S13 File. Duplexes **1**:**5** – **4**:**5** in the presence of higher [HIV-RT]. HIV units used were ca. 0.187 per well, 30 times more than the experiments described on the manuscript.
