# Supplementary material for: Translesion synthesis by AMV, HIV, and MMLVreverse transcriptases using RNA templates containing inosine, guanosine, and their 8-oxo-7,8-dihydropurine derivatives
Source: PLoS One. 2020 Aug 28;15(8):e0235102. doi: 10.1371/journal.pone.0235102 (PMC7455023; doi:10.1371/journal.pone.0235102)
Supplement: S14 File — (PDF) [file pone.0235102.s014.pdf]

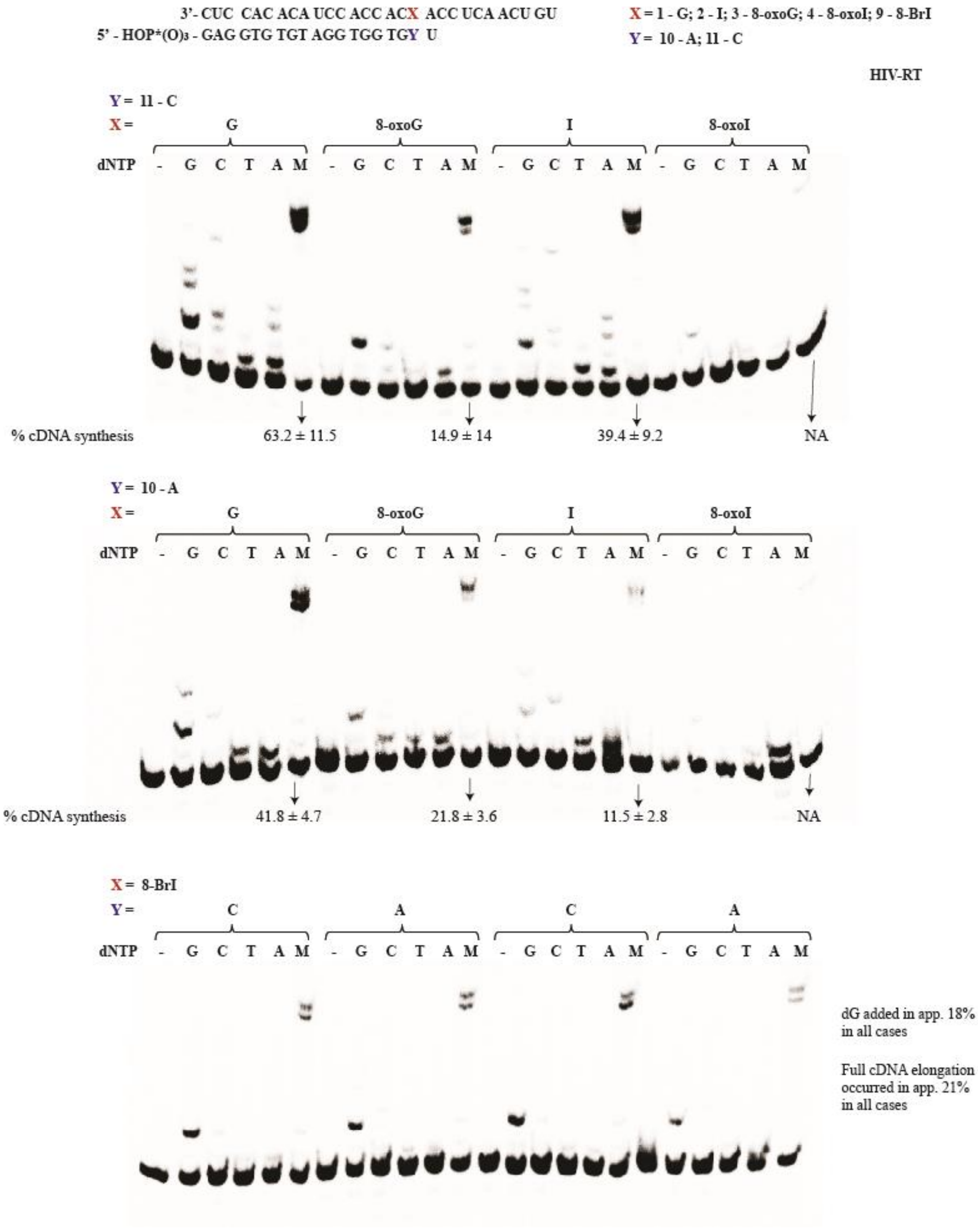

S14 File. Duplexes **1**:**10** – **4**:**10**, **1**:**11** – **4**:**11** and **9**:**10** / **9**:**11** in the presence of HIV-RT
