# Supplementary material for: Translesion synthesis by AMV, HIV, and MMLVreverse transcriptases using RNA templates containing inosine, guanosine, and their 8-oxo-7,8-dihydropurine derivatives
Source: PLoS One. 2020 Aug 28;15(8):e0235102. doi: 10.1371/journal.pone.0235102 (PMC7455023; doi:10.1371/journal.pone.0235102)
Supplement: S15 File — Reactions carried out at rt. (PDF) [file pone.0235102.s015.pdf]

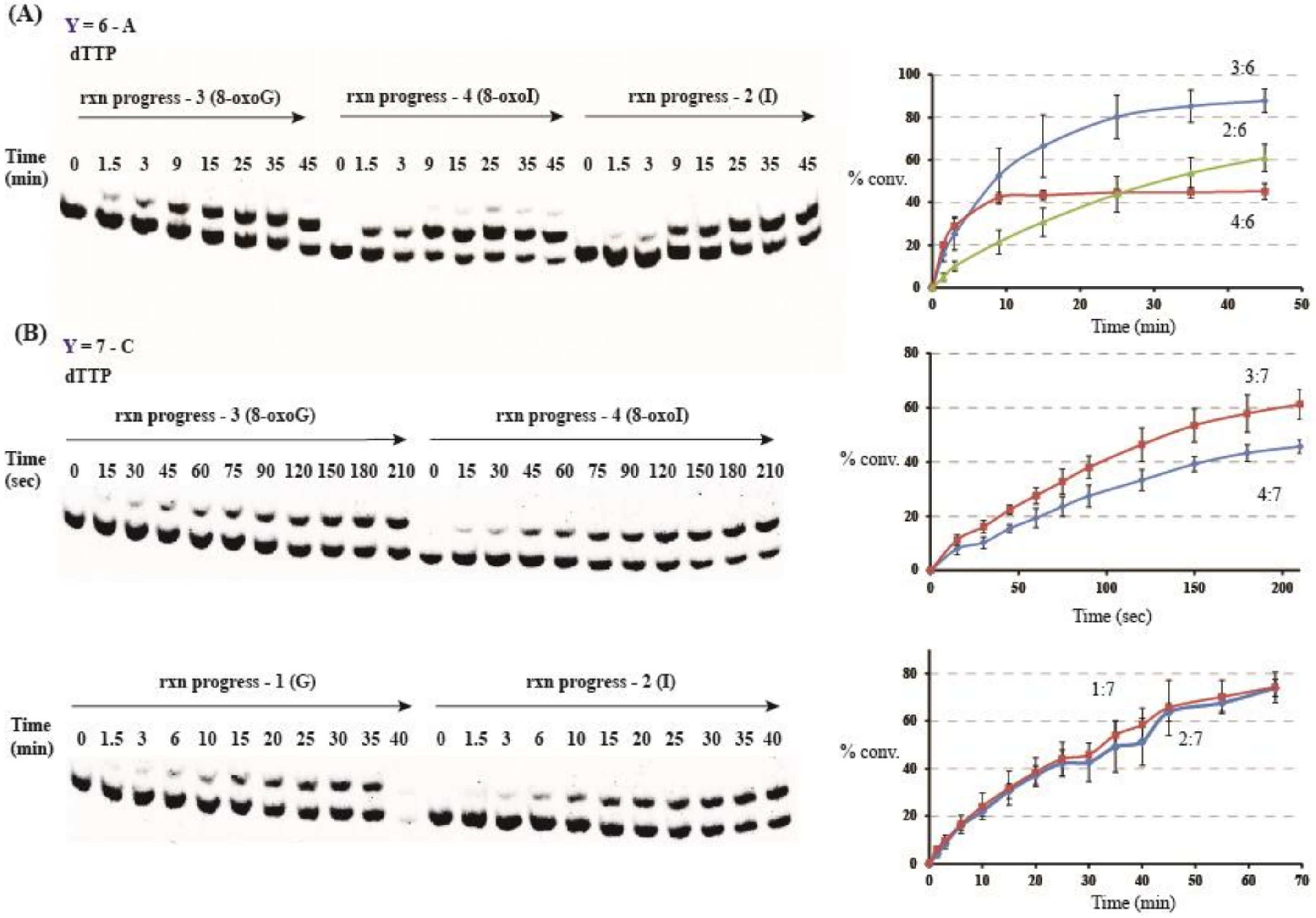

S 15 File. Relative rates for **2**:**6** – **4**:**6** with dTTP and **1**:**7** – **4**:**7** with dTTP at constant [dNTP] and [AMV-RT] as a function of time. Reactions carried out at rt.
