# Supplementary material for: Translesion synthesis by AMV, HIV, and MMLVreverse transcriptases using RNA templates containing inosine, guanosine, and their 8-oxo-7,8-dihydropurine derivatives
Source: PLoS One. 2020 Aug 28;15(8):e0235102. doi: 10.1371/journal.pone.0235102 (PMC7455023; doi:10.1371/journal.pone.0235102)
Supplement: S16 File — (DOCX) [file pone.0235102.s016.docx]

**Translesion Synthesis by MmLV-, AMV-, and HIV-Reverse Transcriptases Using RNA Templates Containing Inosine, Guanosine, and Their 8-oxo-7,8-Dihydropurine Derivatives.**

Madeline Glennon,^†^ Austin Skinner, ^†^ Mara Krutsinger and Marino J. E. Resendiz*

Department of Chemistry, University of Colorado Denver, Science Building 1151 Arapahoe St, Denver, CO 80204, USA

* To whom correspondence should be addressed. Tel: 303-315-7658 ; Email: [marino.resendiz@ucdenver.edu](mailto:marino.resendiz@ucdenver.edu)

^†^ These authors contributed in the same amount to this work.

**Supporting Information Index:**

**Page**  **Contents:**

S3-10...................Experimental details and figures pertaining to the synthesis of 8-oxoI phosphoramidite. Figures S1-S4: H^1^, C^13^, P^31^-NMR, and IR spectra

S11-18.................Experimental details and figures pertaining to the synthesis of 8-bromoI phosphoramidite

Figures S5-S8: H^1^, C^13^, P^31^-NMR, and IR spectra

S19......................Figure S9: MALDI TOF of oligonucleotides **1** - **4**.

S20-21………….Figure S9a: Procedure for MALDI TOF of oligonucleotides **1** – **4** & **9** along with spectra their corresponding spectra.

S22......................Figure S10: Typical CD of RNA/DNA duplex at 20 °C and 85° C; and Figure S11: CD and T_m_ measurement for duplex **1**:**5**.

S23…..................Figure S11: CD and T_m_ measurement for duplexes **1**:**5 -** **4**:**5;** **1**:**6-4:6;** and **9:5, 9:6**.

S24…..................Figure S12: CD and T_m_ measurement for duplexes **1**:**7 -** **4**:**7;** **1**:**10-4:10;** and **9:7, 9:10**.

S25…..................Figure S13: CD and T_m_ measurement for duplexes **1**:**11 -** **4**:**11;** **9:11**; **1**:**8 -** **4**:**8;** and **9:8**

S26…..................Figure S14: T_m_ measurements and ANOVA for duplexes **1:5, 1:6. 1:7, 1:8, 1:10, 1:11.**

S27…..................Figure S15: T_m_ measurements and ANOVA for duplexes **2:5, 2:6. 2:7, 2:8, 2:10, 2:11.**

S28…..................Figure S16: T_m_ measurements and ANOVA for duplexes **3:5, 3:6. 3:7, 3:8, 3:10, 3:11.**

S29…..................Figure S17: T_m_ measurements and ANOVA for duplexes **4:5, 4:6. 4:7, 4:8, 4:10, 4:11.**.

S30…..................Figure S18: T_m_ measurements and ANOVA for duplexes **9:5, 9:6. 9:7, 9:8, 9:10, 9:11.**

S31......................Figure S19: Native PAGE (20 %) of RNA w/wo DNA displaying duplex formation.

S32......................Figure S20: Relative rates for **1**:**5** – **4**:**5** with dCTP and **2**:**5** – **4**:**5** with dATP at constant [dNTP] and

[AMV-RT] as a function of time.

S33…..................Figure S21: Sample single experiments for steady state kinetics Hanes-Woolf plots.

S34…...................Figure S22: Duplexes **9**:**6** and **9**:**7** in the presence of AMV-RT

S34…...................Figure S23: Duplexes **1**:**11** - **4**:**11** in the presence of AMV-RT.

S35……………….Figure S24: Duplexes **1**:**5** - **4**:**5** in the presence of AMV-RT at various pH values.

S36.......................Figure S25: RNA:DNA **1**:**5**-**4**:**5** at higher and lower [MMLV-RT]

S37.......................Figure S26: RNA:DNA **1**:**6**-**4**:**6** & **1**:**7**-**4**:**7** at lower [MMLV].

S38.......................Figure S27: RNA:DNA **1**:**5**-**4**:**5** using SSII (Superscript II).

S39.......................Figure S28: Duplexes **1**:**5** – **4**:**5** in the presence of higher [HIV-RT].

S40.......................Figure S29: Duplexes **1**:**10** – **4**:**10**, **1**:**11** – **4**:**11** and **9**:**10** / **9**:**11** in the presence of HIV-RT

S41.......................Figure S30: Relative rates for **2**:**6** – **4**:**6** with dTTP and **1**:**7** – **4**:**7** with dTTP at constant [dNTP] and

[AMV-RT] as a function of time. Reactions carried out at rt.

S42.......................Figure S31: Steady state kinetics sample gel for **1**:**8** – **4**:**8** with dTTP at decreasing [dNTP] and

constant [AMV-RT]

S43......................References

**Experimental Details for the Synthesis of the Phosphoramidites Used in this Work.**

**General Information.** ^1^H NMR and ^13^C NMR spectra were recorded at 300, and 75 MHz, respectively. IR spectra were recorded on a diamond ATR sampler using powders of pure materials, or of photoreactions at various time intervals. Methylene chloride was distilled over calcium hydride. Tetrahydrofuran was distilled over sodium and benzophenone. All other reagents were used as purchased without further purification. All intermediates and compounds analyzed for HRMS were carried out via ESI/APCI. UV-vis spectroscopy was carried out on a Perkin Elmer λ-650 UV/vis spectrometer. The unusual solubility of inosine and its derivatives was pointed out in a previous study, where the synthesis for the phosphoramidite of I is reported,^1,2^ and is something that was experienced for the 8-oxoI and 8-BrI derivatives.


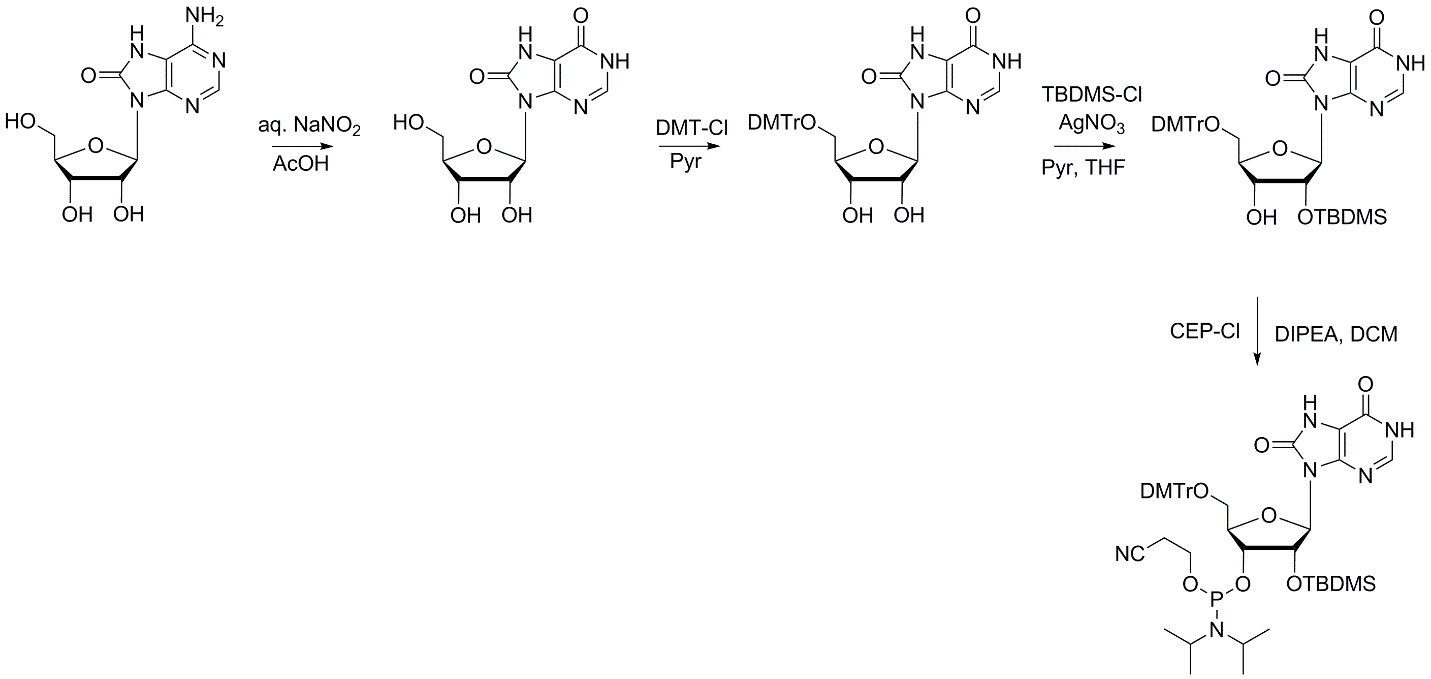


8-oxoA S1 S2 S3

S4

**8-oxo-7,8-dihydroinosine** (S1) ^3^:

8-oxo-adenosine (6.6 g, 23.3 mmol) was added to a flask charged with a stirring bar, and dissolved in glacial acetic acid (120 mL). In a separate flask NaNO_2_ (9.7 g, 140.6 mmol) was dissolved in water (45 mL) and the resultant solution was slowly added to the nucleoside solution while venting evolved gas over the first two hours. Reaction was left stirring at room temperature overnight, followed by bubbling with a stream of air for 3 hours. The residual solvent was concentrated under reduced pressure and the resultant solid was precipitated in 250 mL of 1:1 ethanol/water. The obtained powder was isolated by vacuum filtration to yield **8-oxoI** in the form of a yellowish solid 5 g (17.6 mmol, 65-90%). ^1^HNMR (DMSO d-6): δ 7.94 (s, 1H), 5.67 (d, 1H), 4.83 (t, 1H), 4.12 (t, 1H), 3.86 (q, 1H), 3.60 (dd, 1H), 3.46 (dd, 1H). ^13^CNMR (DMSO d-6): δ 160.49, 152.75, 150.82, 145.44, 108.60, 86.25, 71.84, 71.23, 63.16. FTIR (cm^-1^): 3224.14, 2713.66, 1640, 1624.90, 1578.48. HRMS (m/z): 284.0768. All obtained spectra was in agreement with previously reported data.

**5’-O-(4,4’-dimethoxytrityl)-8-oxo-7,8-dihydroinosine** (S2):

8-oxo-inosine (1.5523 g, 5.5 mmol) was azeotropically dried over anhydrous pyridine (9 mL). Anhydrous pyridine (60 mL) was added to dry solid and cooled to 0 °C. 4,4′-dimethoxytrityl chloride (2.003 g, 5.9 mmol) was added in two portions under an atmosphere of argon. Reaction was stirred overnight and quenched with deionized water (100 mL), followed by extraction with dichloromethane (3 × 100 mL). Organic layers were combined and washed with brine (3 × 100 mL). Organic layers were then concentrated under reduced pressure to yield an oil. Purification via column chromatography was carried out using a gradient from 100% dichloromethane to 20% methanol in dichloromethane. Fractions were analyzed by TLC with an eluent of 20 % methanol in dichloromethane. Fractions were combined and concentrated under reduced pressure to yield S2 in the form of a white foam (0.500 g, 0.852 mmol, 10%) ^1^HNMR (DMSO d-6): δ 11.41 (s, 1H), 7.84 (s, 1H), 7.37 (d, 2H), 7.24 (m, 7H), 6.82 (m, 4H), 5.68 (d, 1H), 5.28 (d, 1H) 5.00 (d, 1H), 4.77 (t, 1H), 4.31 (t, 1H), 3.94 (t, 1H), 3.15 (m, 2H). ^13^CNMR (DMSO d-6): δ 158.45, 152.13, 151.33, 145.50, 144.65, 136.19, 130.14, 128.23, 127.00, 113.48, 109.10, 86.86, 85.75, 82.84, 71.21,70.80, 64.52, 55.48, 46.21. FTIR (cm^-1^): 3037.14, 2930.33, 1711.11, 1673.69, 1606.33, 1556.93, 1507.17, 1442.00. HRMS (m/z): 586.2070

**2’-(t-butyldimethylsilyl)-5’-O-(4,4’-dimethoxytrityl)-8-oxo-7,8-dihydroinosine** (S3):

S2 (1.5615 g, 2.7 mmol) and AgNO_~~3~~_ (0.545 g, 3.2 mmol) were added to a foil covered flame dried flask charged with a stirring bar and the combined contents were placed under reduced pressure for 30 minutes. Anhydrous tetrahydrofuran (36 mL) and pyridine (5.45 mL) were added under an atmosphere of argon. Tert-butyldimethylchlorosilane (0.201 g, 3.3 mmol) was added and stirred over five hours. A second portion of TBDMS-Cl (0.4016 g, 1.3 mmol) and AgNO_3_ (0.226 g, 1.3 mmol) were added and left stirring overnight. The resulting suspension was filtered and the filtrite was recollected and partitioned with ethyl acetate (50 mL) and 20% NaHCO_3_ (20 mL). The organic layer was washed over deionized water (2 × 25 mL), and brine (1 × 25 mL). Organic layers were concentrated to a crude oil and purified by column chromatography using a gradient to 3% methanol in ethyl acetate. The desired product was the first spot to elute off the column. Fractions containing the desired regioisomer were combined and concentrated to yield a white foam corresponding to S3 (0.5 g, 0.713 mmol, 27%). ^1^HNMR (DMSO d-6): δ 11.43 (s, 1H), 8.58 (s, 1H), 7.80 (m, 2H), 7.38 (m, 4H), 7.25 (m, 6H), 6.84 (m, 4H), 5.69 (m, 1H) 4.94 (m, 1H), 4.85 (m, 1H), 4.24 (m, 1H), 3.95 (t, 1H), 3.18 (m, 1H), 3.11 (m, 1H), 0.77 (s, 9H), -0.02 (s, 3H),-0.08 (s, 3H). ^13^CNMR (DMSO d-6): 157.95, 151.54, 150.77, 146.57, 144.98, 144.42, 144.08, 136.06, 135.63, 129.70, 127.62, 126.50, 123.84, 113.00, 108.60, 85.96, 85.24, 82.56, 72.20, 70.17, 63.56, 54.95, 25.57, 12.87, -4.80, -5.23. FTIR (cm^-1^): 3035.2, 2929.14, 2856.21, 1711.11, 1678.15, 1606.68, 1556.16, 1507.63, 1441.02. HRMS (m/z): 700.2964

**2'-O-(t-butyldimethylsilyl)-3’-O-[(2-ethylcyano-N,N-diisopropylphosphoramidite)-5'-O-(4,4'-dimethoxytrityl)-8-oxo-7,8-dihydroinosine** (S4):

S3 (0.3 g, 0.42 mmol) was added to a flame dried flask charged with a stirring bar and dried under vacuum for an hour. Dichloromethane (0.85 mL) and diisopropylethylamine (0.45 mL) were added under an atmosphere of argon. 2-Cyanoethyl N,N-diisopropylchlorophosphoramidite (CEP-Cl) (0.145 mL) was added to the solution and stirred at room temperature for one hour. After one hour additional CEP-Cl (0.15 mL) was added and the reaction was stirred for additional 30 minutes. The reaction mixture was then quenched over 20 % NaHCO_3_ (20 mL) and extracted with dichloromethane (3 × 25 mL). The combined organic extracts were then washed with deionized water (2 × 25 mL) and brine (1 × 25 mL). The resultant organic layer was concentrated under reduced pressure and purified via column chromatography using a gradient from 40% to 60% acetone in dichloromethane. Fractions containing product were concentrated under reduced pressure to yield phosphoramidite S4 in the form of a white powder (0.15 g, 0.17 mmol, 39%). ^1^HNMR (CDCl_3_): δ 8.81 (d, 1H) 7.53 (d, 1H) 7.39 (m, 2H) 7.29 (m, 4H), 7.16 (m, 6H), 6.84 (m, 4H) 6.77 (m, 2H), 5.98 (d, 1H), 5.93 (d, 1H), 5.49 (m, 1H) 5.00 (m, 1H), 4.39 (m, 1H), 4.32 (m,1H), 4.15 (m, 1H), 3.81 (d, 6H), 3.74 (d, 6H), 3.50 (m, 1H) 3.21 (m, 1H), 0.88 (s, 9H), 0.83 (s, 9H), 0.10 (s, 3H), 0.00 (s, 3H), -0.03 (s, 3H), -0.15 (s, 3H).^31^PNMR (CDCl_3_): δ 150.72, 148.46. HRMS (m/z): 899.4036.


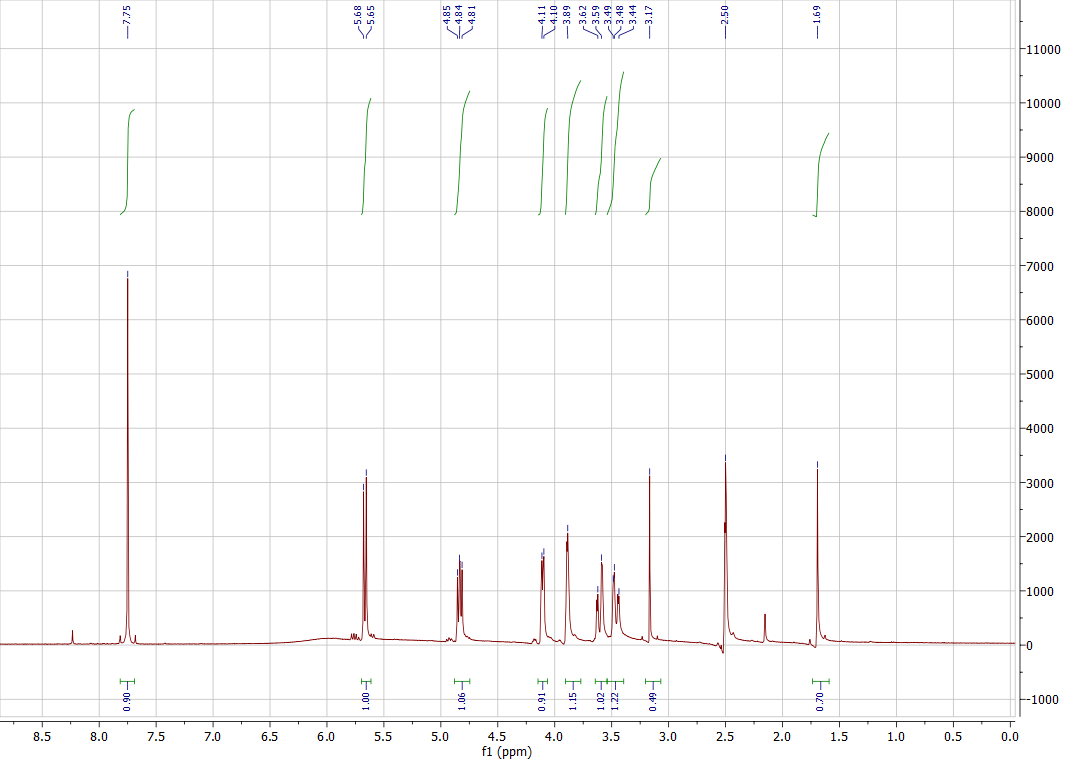


**Figure S1A:** ^1^HNMR spectrum of S1


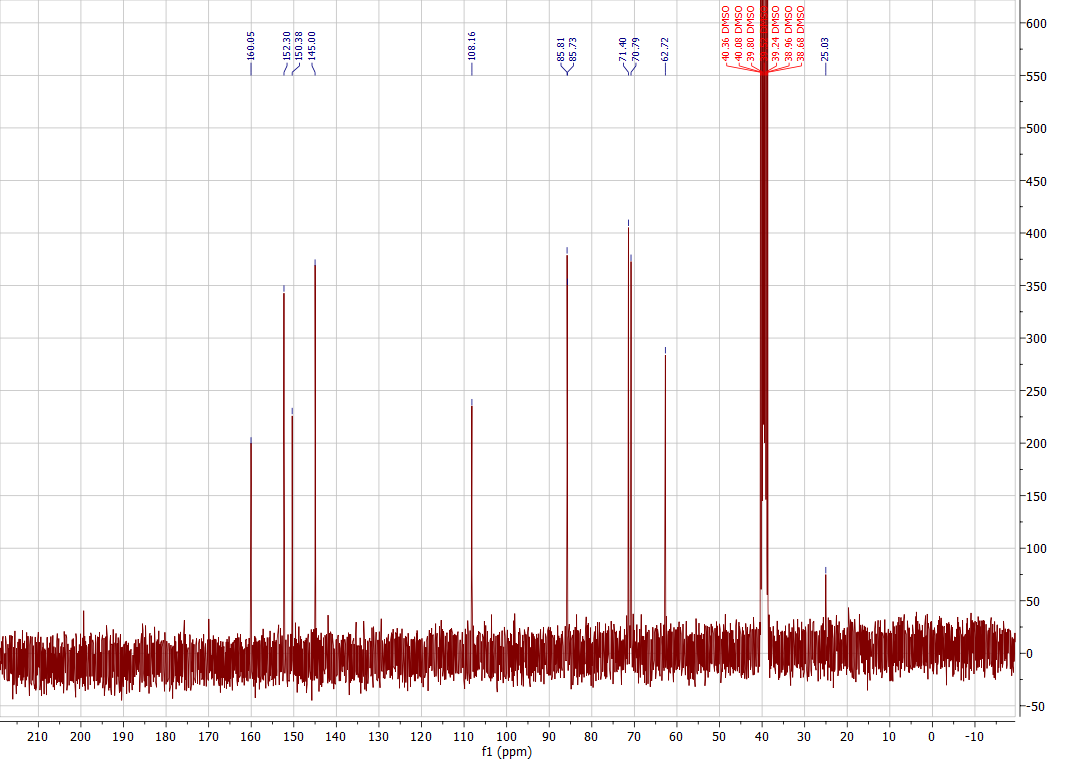


**Figure S1B:** ^13^CNMR spectrum of S1


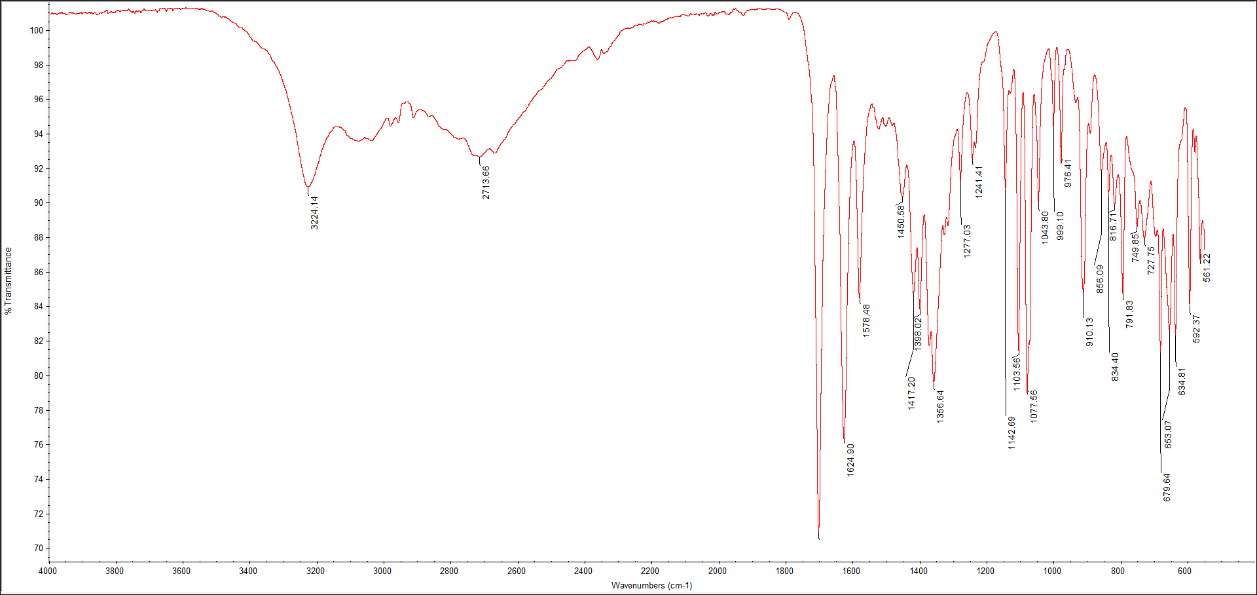


**Figure S1C:** FTIR spectrum of S1


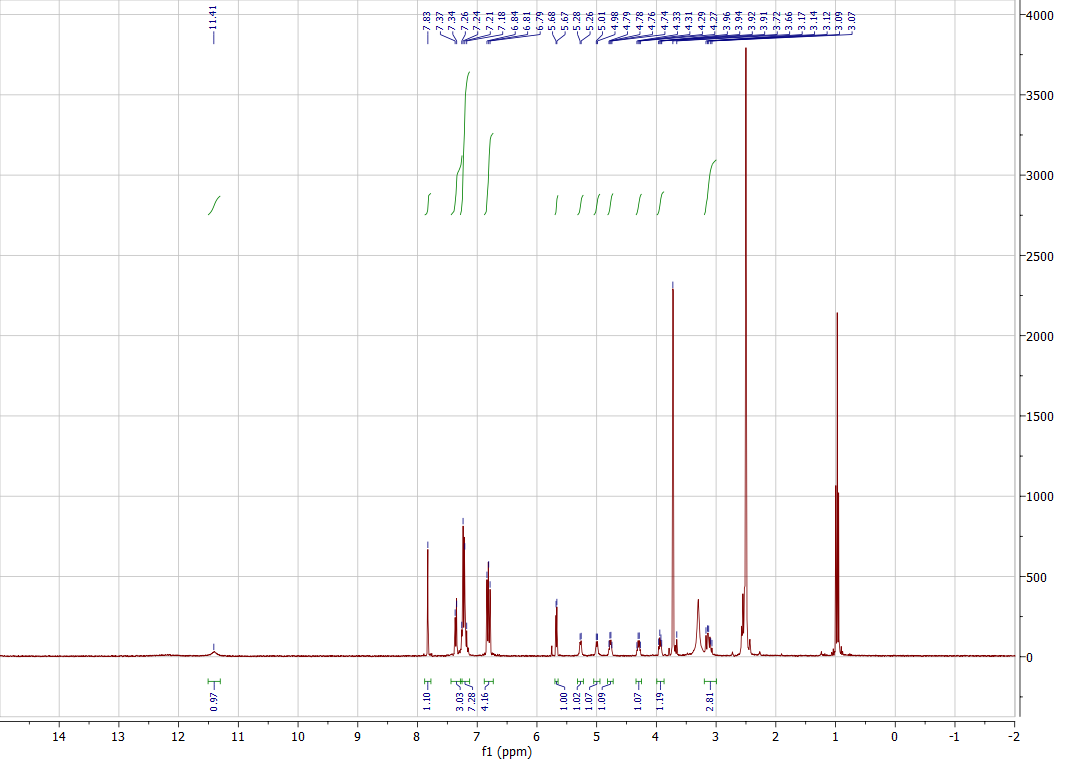


**Figure S2A:** ^1^HNMR spectrum of S2


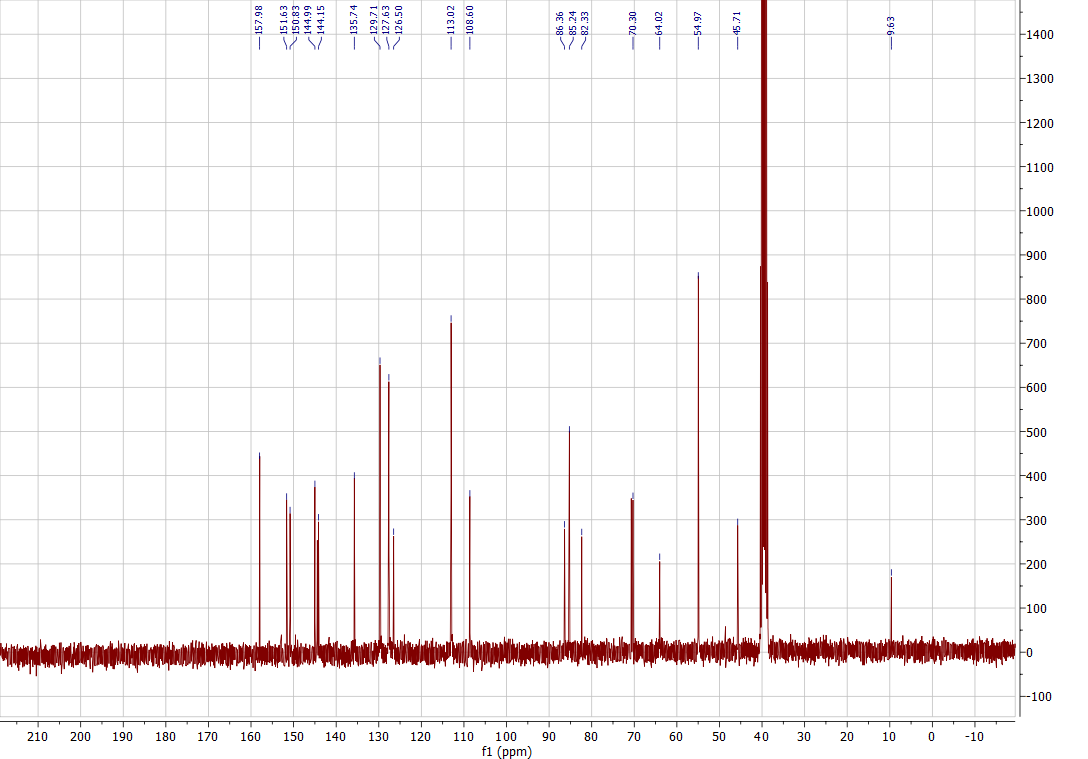


**Figure S2B:** ^13^CNMR spectrum of S2


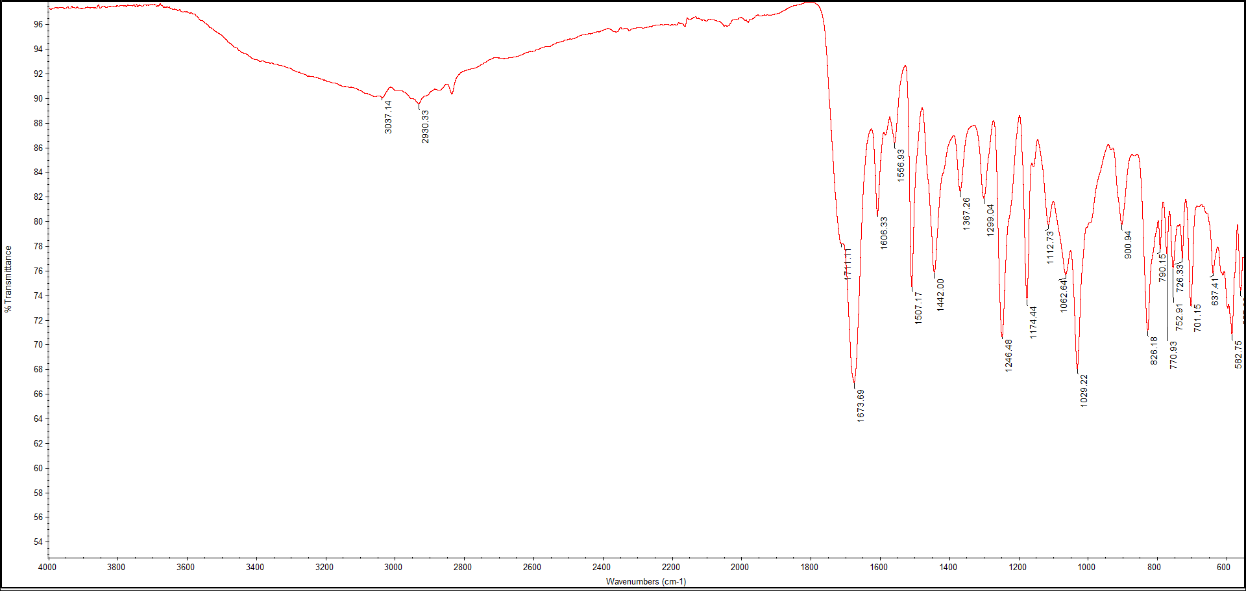


**Figure S2C:** FTIR spectrum of S2


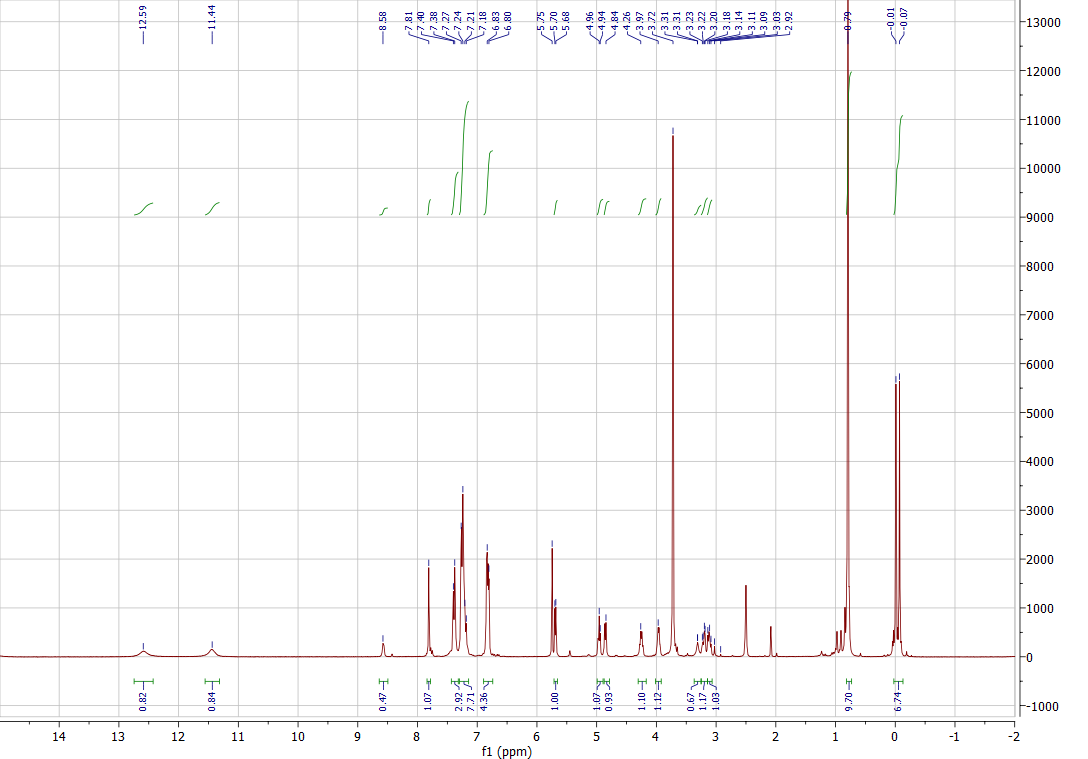


**Figure S3A:** ^1^HNMR spectrum of S3


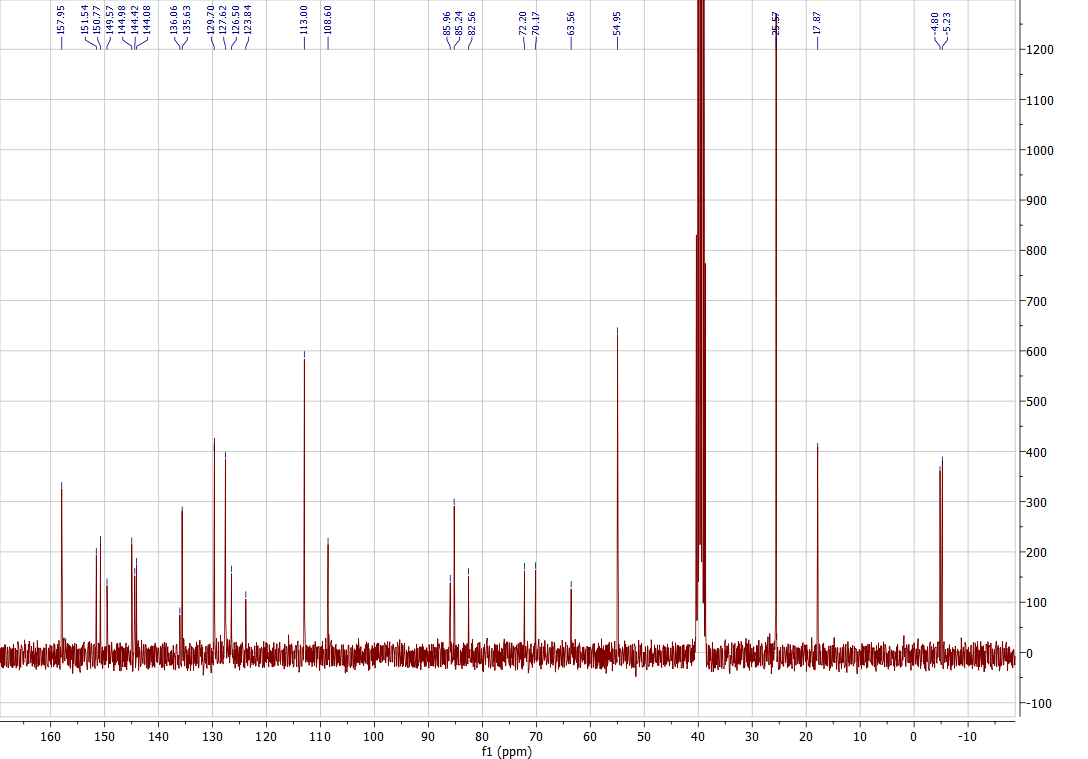


**Figure S3B:** ^13^CNMR spectrum of S3


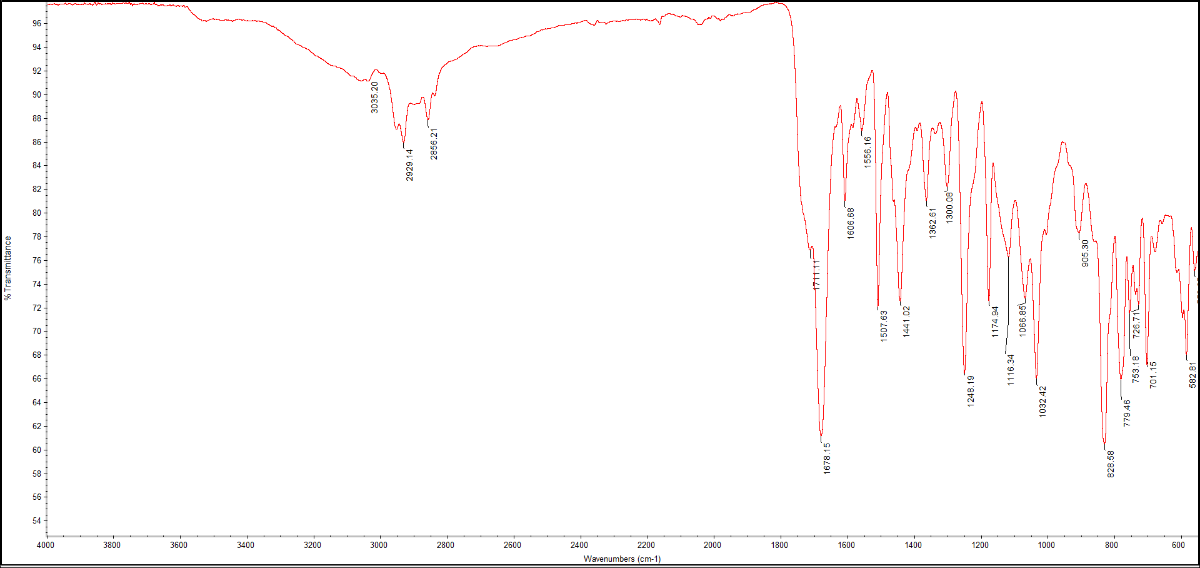


**Figure S3C:** FTIR spectrum of S3


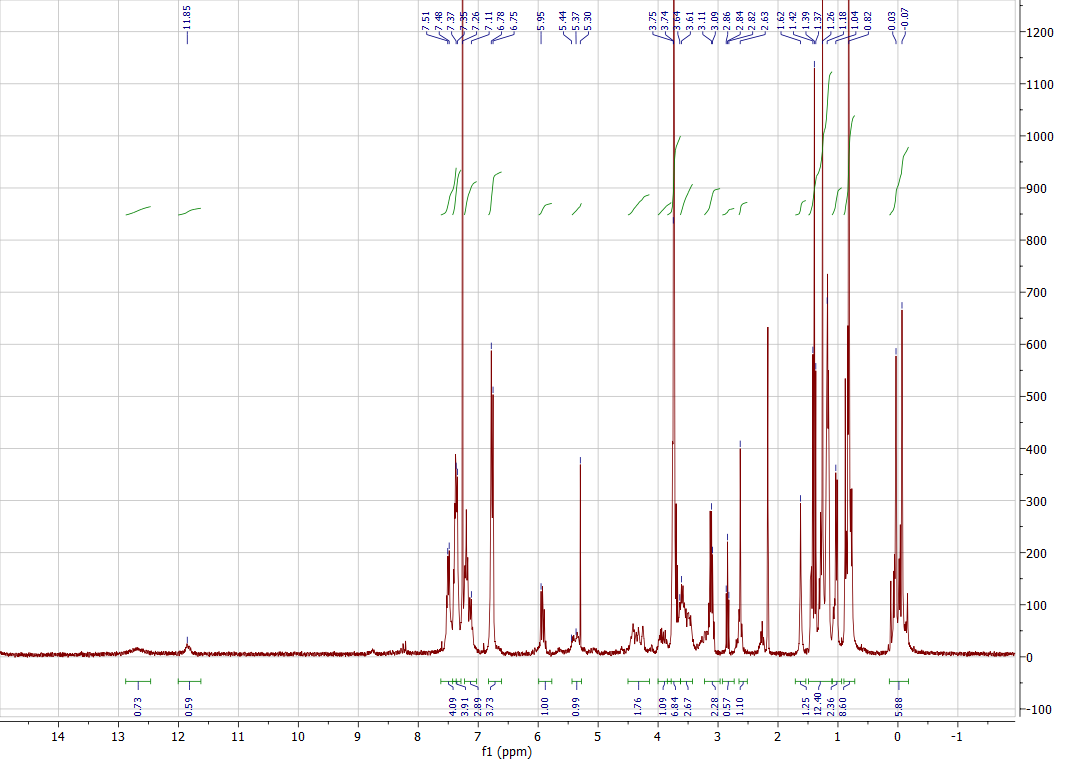


**Figure S4A:** ^1^HNMR spectrum of S4


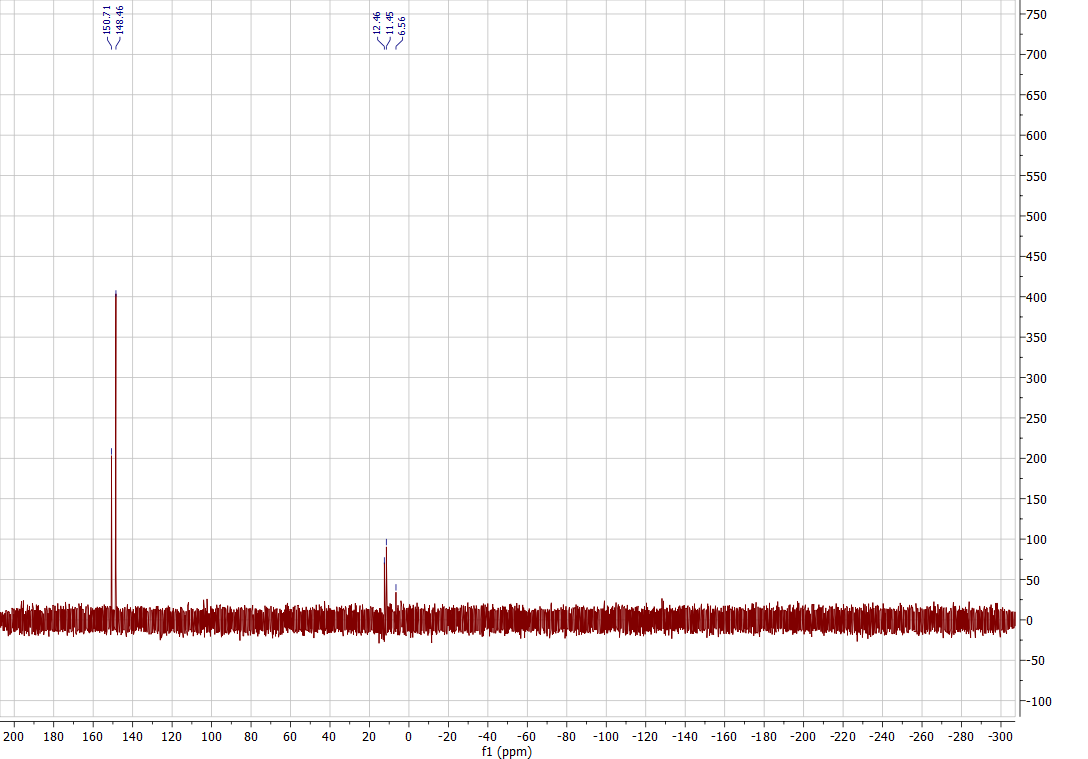


**Figure S4B:** ^31^PNMR spectrum of S4


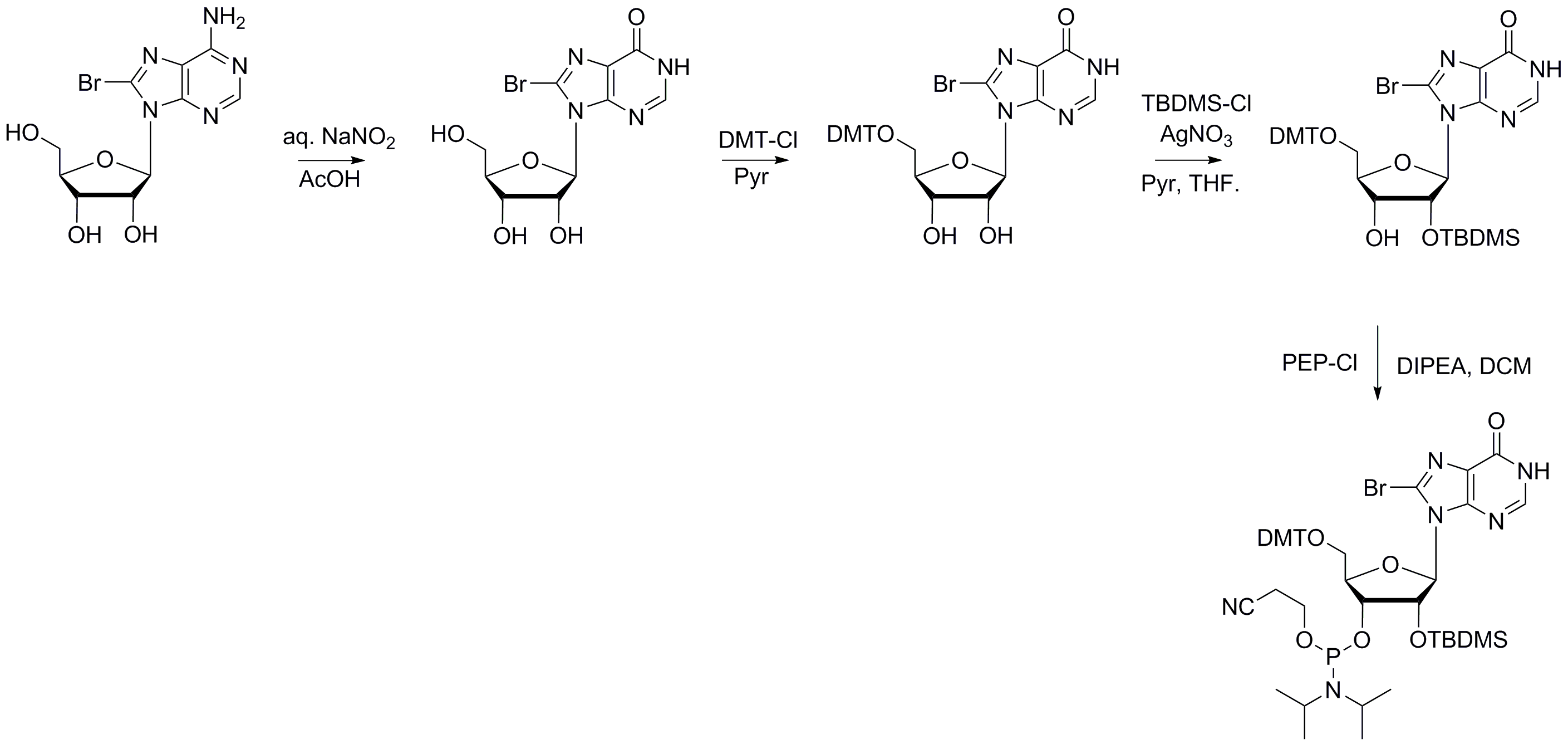


8-BrA S5 S6 S7

S8

**8-Bromoinosine** (S5)^4^:

8-bromo-adenosine (8.5709 g, 24.6 mmol) was added to flask charged with a stirring bar and dissolved in glacial acetic acid (150 mL). In a separate flask NaNO_2_ (10.1902 g, 147.7 mmol) was dissolved in 50 mL deionized water and the resultant solution was slowly added to the nucleoside solution, while venting the evolved gas over ca. 2 h. The reaction mixture was stirred at room temperature overnight, and bubbled with a flow of air over 3 h. The residual solvent was concentrated under reduced pressure and a solid precipitated following the addition of a 100 mL of 4:1 ethanol/water solution. An orange solid was then obtained upon filtration to yield 8-bromoinosine, 5 g (18.0 mmol, 50-85%) ^1^HNMR (DMSO d-6): δ 7.94 (s, 1H), 5.67 (d, 1H), 4.83 (t, 1H), 4.12 (t, 1H), 3.86 (q, 1H), 3.60 (dd, 1H), 3.46 (dd, 1H). ^13^CNMR (DMSO d-6): δ 155.58, 149.21, 146.53, 125.98, 125.37, 90.49, 86.30, 71.21, 70.50, 61.89. FTIR (cm^-1^): 3492.73, 3347.71, 3180.44, 2924.03, 1676.00, 1588.02, 826.40. HRMS (m/z): 284.0768.

**5’-O-(4,4’-dimethoxytrityl)-8-bromo-inosine** (S6):

8-bromoinosine (6.086 g, 17.5 mmol) was azeotropically dried over anhydrous pyridine (35 mL). Anhydrous pyridine (90 mL) was added to dry solid and cooled to 0 °C. 4,4′-dimethoxytrityl chloride (6.43 g, 19.0 mmol) was added under an atmosphere of argon. The resulting solution was stirred overnight and quenched over 20% NaHCO_3_ (100 mL) followed by consecutive washes with ethyl acetate (3 × 100 mL). The combined organic residues were combined and washed with deionized water (3 × 100 mL) and brine (1 × 100 mL). The organic extracts were then concentrated under reduced pressure to an oil. Purification was then achieved via column chromatography using a gradient from 100% DCM to 20% methanol in dichloromethane. Fractions were analyzed by TLC with an eluent of 20 % methanol in dichloromethane. The fractions of interest were combined and concentrated under reduced pressure to yield compound S6 in the form of a white foam (8.5455 g, 13.5 mmol, 75%) ^1^HNMR (DMSO d-6): δ 11.41 (s, 1H), 7.84 (s, 1H), 7.37 (d, 2H), 7.24 (m, 7H), 6.82 (m, 4H), 5.68 (d, 1H), 5.28 (d, 1H) 5.00 (d, 1H), 4.77 (t, 1H), 4.31 (t, 1H), 3.94 (t, 1H), 3.15 (m, 2H). ^13^CNMR (DMSO d-6): δ 157.96, 155.26, 149.06, 145.83, 144.88, 135.68, 135.57, 129.70, 129.58, 127.66, 126.55, 126.27, 125.20, 112.98, 85.23, 83.39, 71.08, 70.03, 63.30, 54.99, 48.60, 45.71. FTIR (cm^-1^): 3056.08, 2930.36, 2834.62, 1682.86, 1606.15, 1584.34, 1507.14, 826.40. HRMS (m/z): 586.2070

**2’-(t-butyldimethylsilyl)-5’-O-(4,4’-dimethoxytrityl)-8-bromo-inosine** (S7):

S6 (8.5 g, 13.1 mmol) and AgNO_~~3~~_ (2.89 g, 17 mmol) were added to a foil covered flame dried flask charged with a stirring bar and dried under reduced pressure for 30 minutes. Anhydrous tetrahydrofuran (36 mL) and pyridine (8 mL) were added under an atmosphere of argon. Tert-butyldimethylchlorosilane (2.367 g, 15.7 mmol) was added quickly under an atmosphere of argon and left to react for four hours. Additional TBDMS-Cl (1.112 g, 7.4 mmol) and AgNO_3_ (0.986 g, 5.8 mmol) were added and the obtained suspension was stirred overnight. The milky suspension was then filtered, and the collected filtrite was concentrated under reduced pressure to an oily solid. Ethyl acetate (100 mL) and 20% NaHCO_3_ (100 mL) were added to partition the crude product. Aqueous layer was extracted over ethyl acetate (5 × 100 mL). Organic layer was washed with deionized water (2 × 100 mL) and brine (1 × 100 mL). Organic layer was concentrated and the residues were purified via column chromatography using a gradient of 100% dichloromethane to 15% acetone in dichloromethane. Fractions containing the desired regioisomer were concentrated under reduced pressure to yield nucleoside S7 in the form of a white foam (1.1 g, 1.4 mmol, 11.1%) ^1^HNMR (DMSO d-6): δ 11.43 (s, 1H), 8.58 (s, 1H), 7.80 (m, 2H), 7.38 (m, 4H), 7.25 (m, 6H), 6.84 (m, 4H), 5.69 (m, 1H) 4.94 (m, 1H), 4.85 (m, 1H), 4.24 (m, 1H), 3.95 (t, 1H), 3.18 (m, 1H), 3.11 (m, 1H), 0.77 (s, 9H), -0.02 (s, 3H),-0.08 (s, 3H). ^13^CNMR (DMSO d-6): 157.99, 155.17, 149.61, 149.11, 145.83, 144.90, 136.1, 135.55, 129.73, 127.67, 126.58, 125.28, 123.89, 113.03, 90.43, 85.30, 83.95, 72.71, 69.95, 62.99, 54.99, 25.48, 17.78, -4.83, -5.43. FTIR (cm^-1^): 3056.34, 2929.99, 1682.85, 1606.46, 1584.42, 1507.44, 827.02. HRMS (m/z): 700.2964

**2'-O-(t-butyldimethylsilyl)-3’-O-[(2-ethylcyano-N,N-diisopropylphosphoramidite)-5'-O-(4,4'-dimethoxytrityl)-8-bromo-inosine** (S8):

Nucleoside S7 (1.0128 g, 1.3 mmol) was added to a flame dried flask charged with a stirring bar and dried under reduced pressure for an hour. Dichloromethane (2.15 mL) and diisopropylethylamine (1.38 mL) were added under an atmosphere of argon. 2-Cyanoethyl N,N-diisopropylchlorophosphoramidite (0.45 mL) was added to the clear solution and stirred at room temperature for one hour. Additional 2-Cyanoethyl N,N-diisopropylchlorophosphoramidite (0.15 mL) was added and the reaction was stirred for another 30 minutes. The reaction was then quenched over 20 % NaHCO_3_ (20 mL) and extracted with dichloromethane (3 × 25 mL). The organic layer was combined and washed with deionized water (2 × 25 mL) and brine (1 × 25 mL). The resultant solution was concentrated under reduced pressure followed by purification via column chromatography using a gradient from 0% to 10% acetone in dichloromethane. The fractions of interest were concentrated under reduced pressure to yield phosphoramidite S8 in the form of a white foam (0.96 g, 1.0 mmol, 75.6%). ^1^HNMR (CDCl_3_): δ 12.55 (s, 1H) 8.02 (d, 1H) 7.45 (d, 2H) 7.37 (m, 5H) 7.21 (m, 2H), 6.77 (m, 4H), 5.99 (m, 1H) 5.38 (m, 1H), 5.42 (m, 1H), 4.44 (m, 2H), 4.37 (m, 2H) 4.21 (m, 2H), 4.15 (m, 2H), 3.65 (s, 6H), 3.55 (m, 3H), 3.81 (d, 6H), 3.74 (d, 6H), 3.50 (m, 1H) 3.21 (m, 1H), 0.77 (s, 9H), -0.04 (s, 3H), -0.25 (s, 3H).^31^PNMR (CDCl_3_): 151.49, 148.52. HRMS (m/z): ^79^Br 961.3349, ^81^Br 963.3349


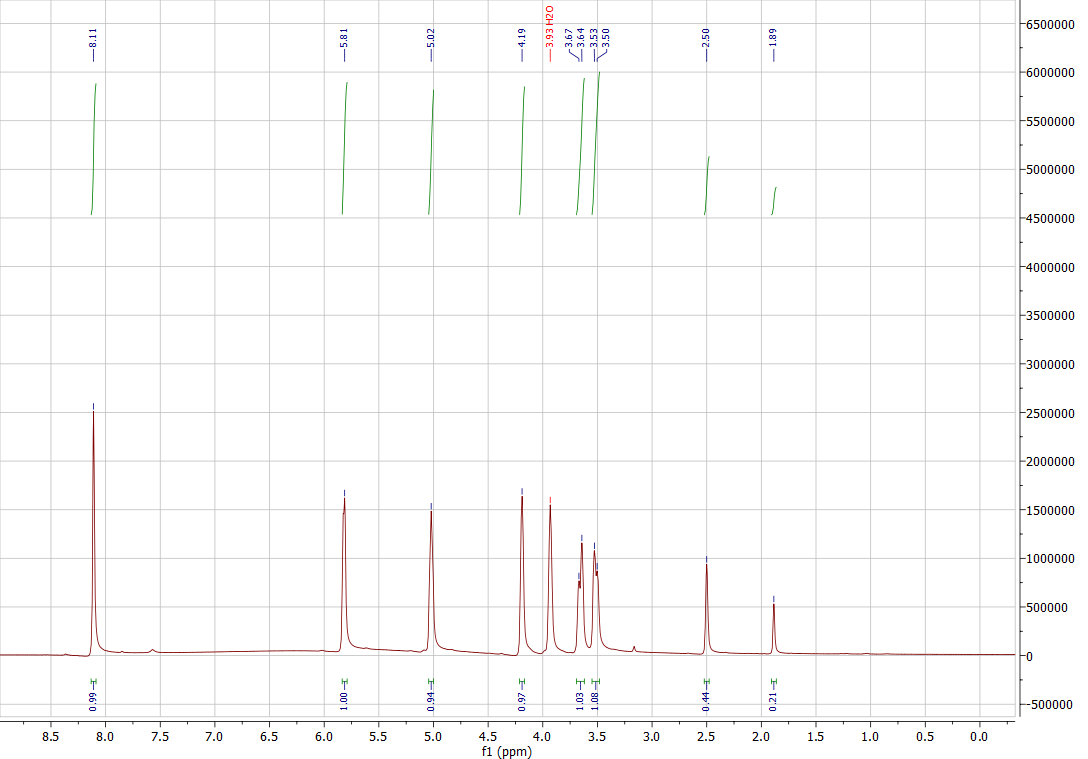


**Figure S5A:** ^1^HNMR spectrum of S5


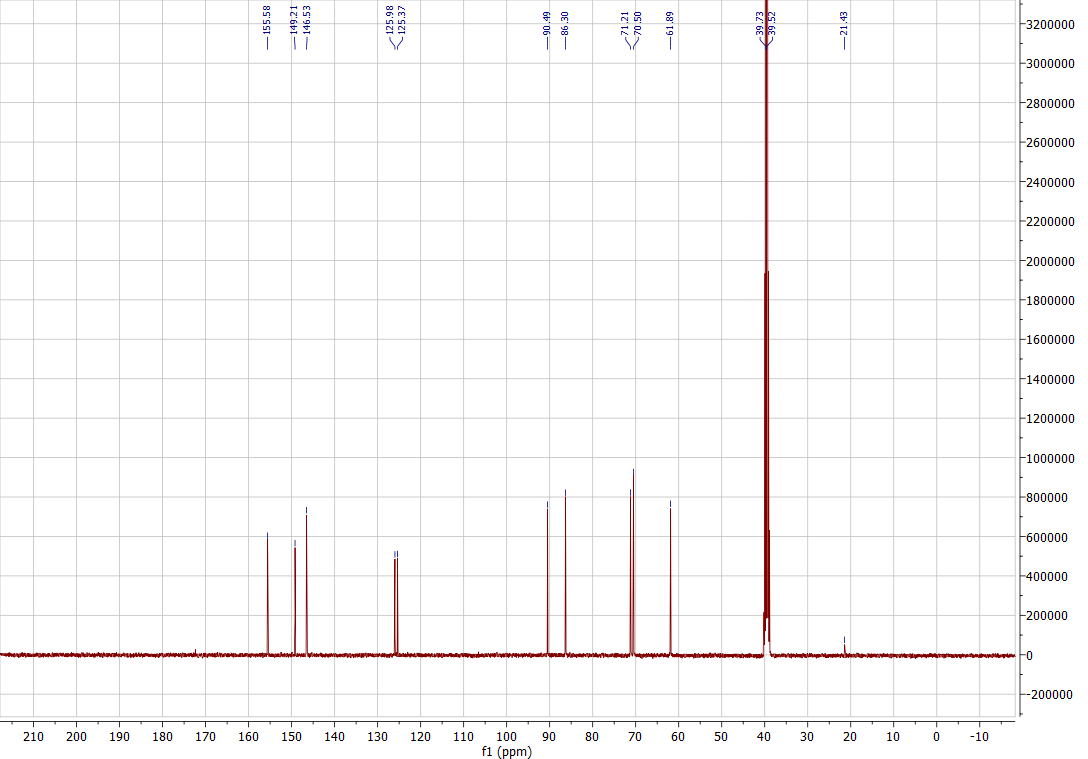


**Figure S5B:** ^13^CNMR spectrum of S5


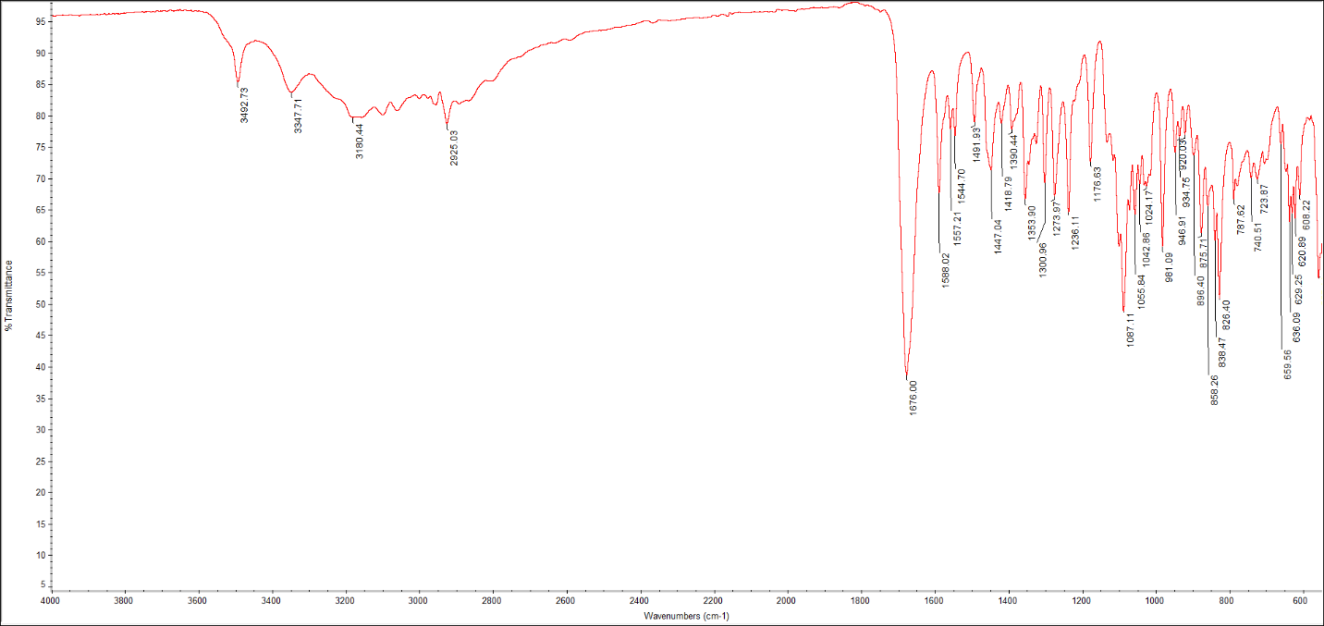


**Figure S5C:** FTIR spectrum of S5


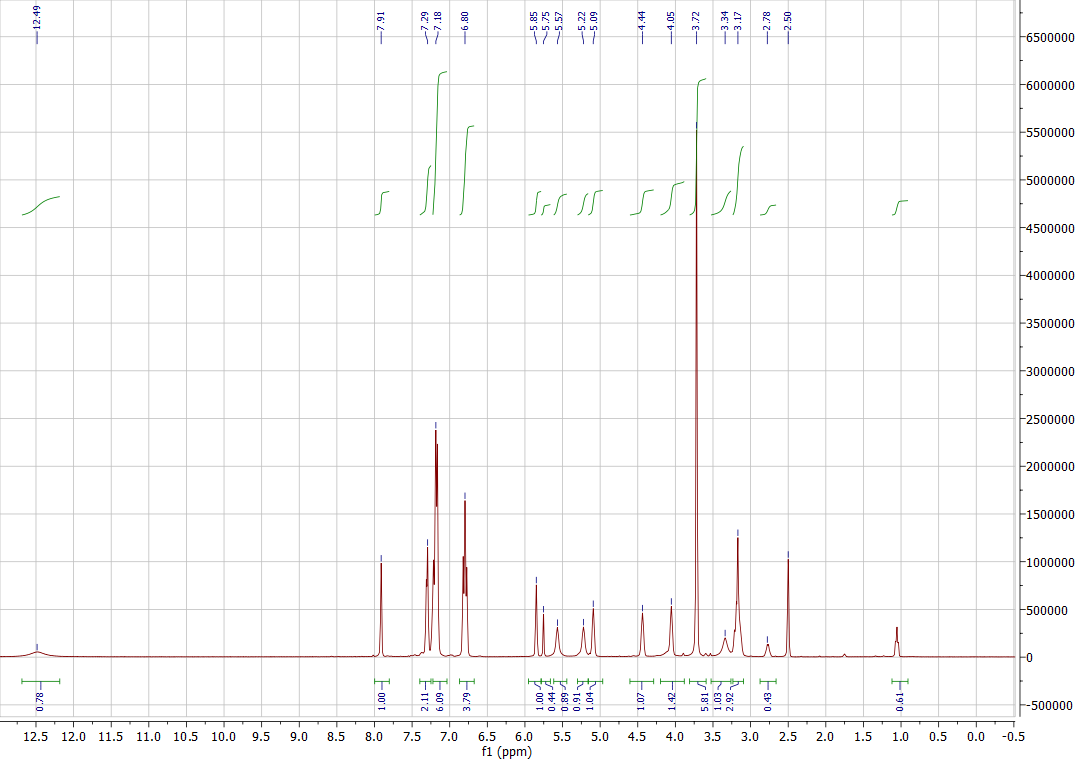


**Figure S6A:** ^1^HNMR spectrum of S6


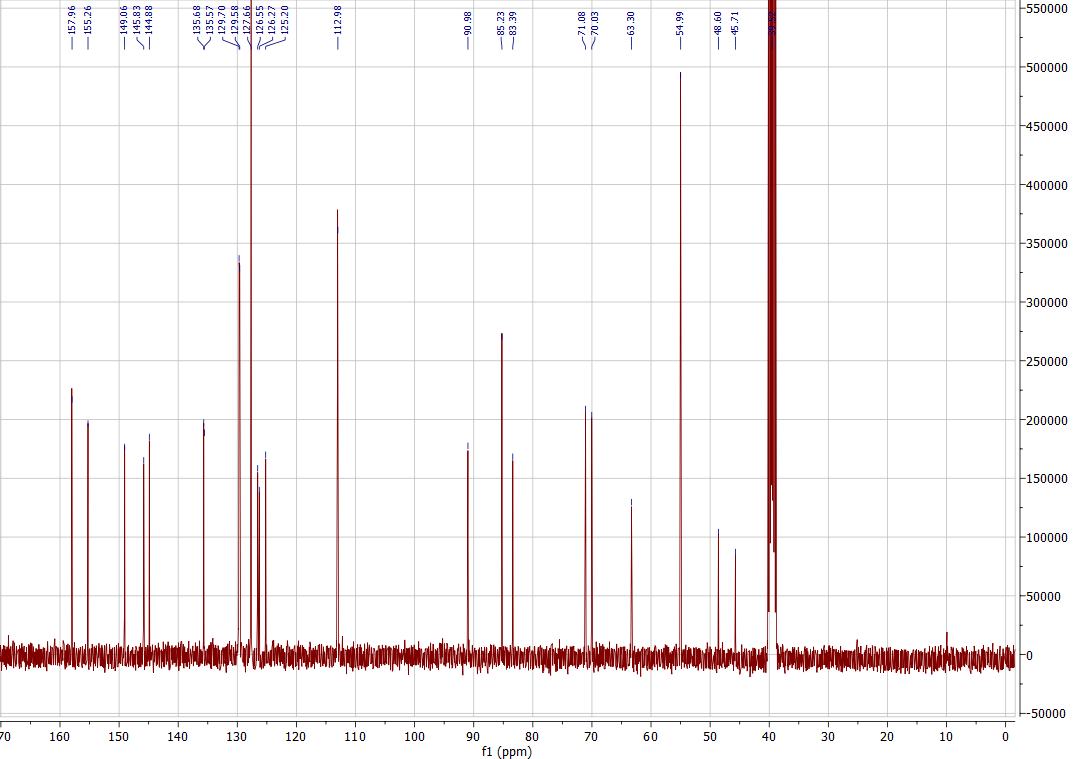


**Figure S6B:** ^13^CNMR spectrum of S6


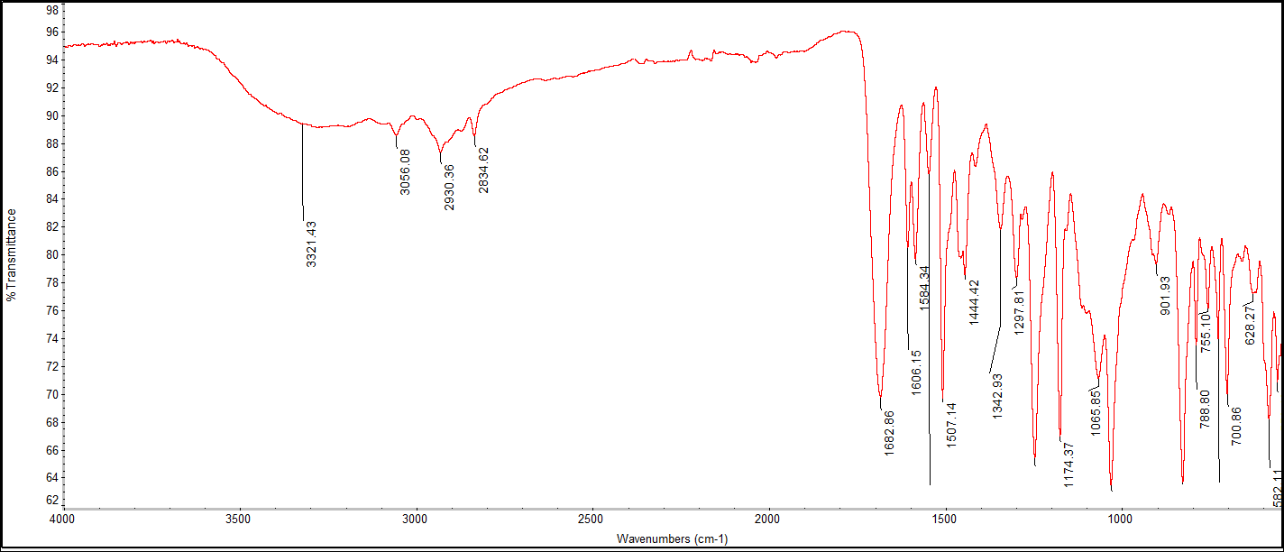


**Figure S6C:** FTIR spectrum of S6


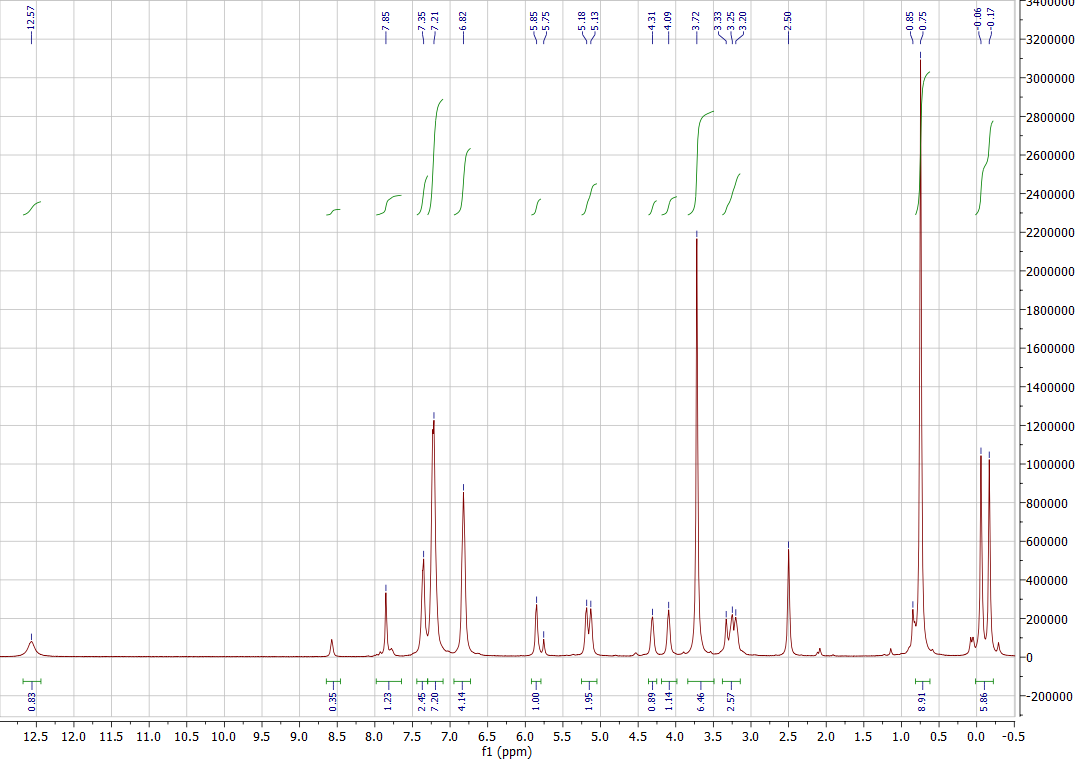


**Figure S7A:** ^1^HNMR spectrum of S7


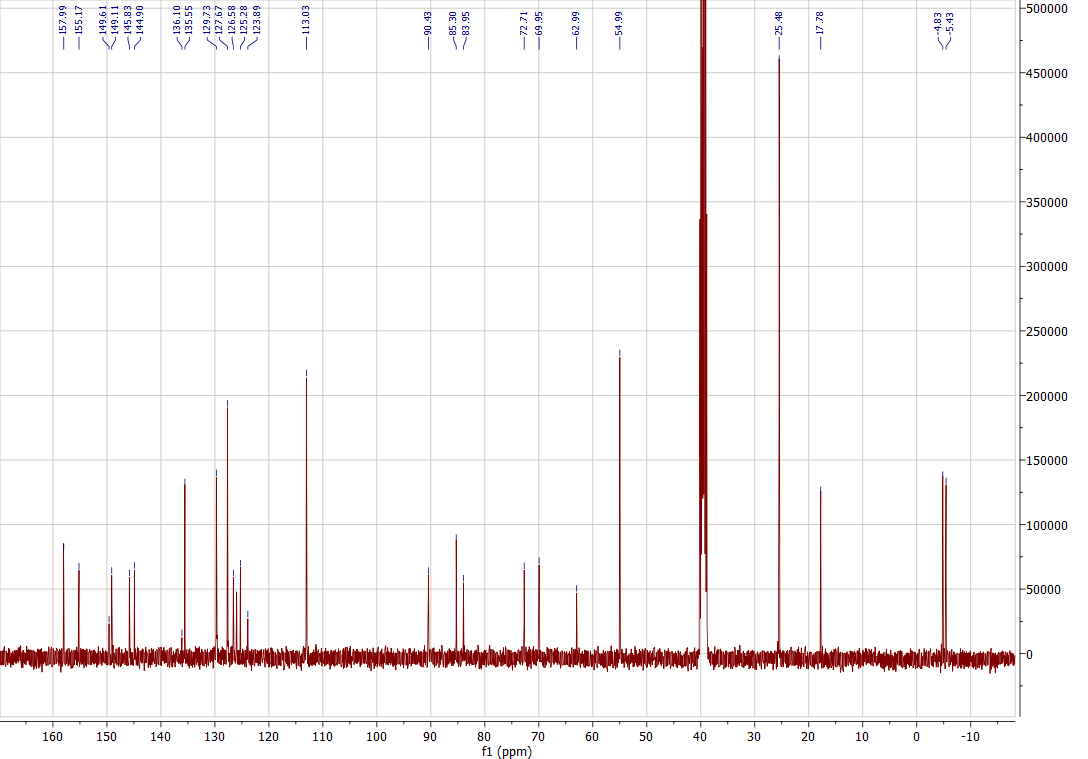


**Figure S7B:** ^13^CNMR spectrum of S7


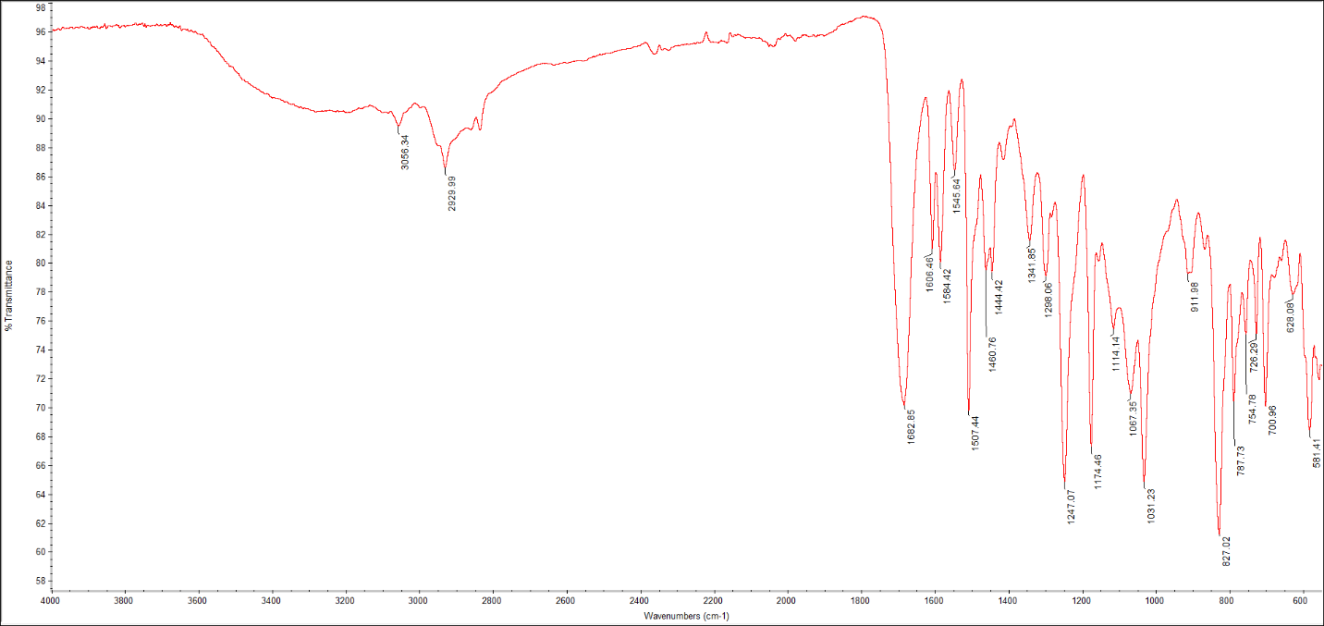


**Figure S7C:** FTIR spectrum of S7


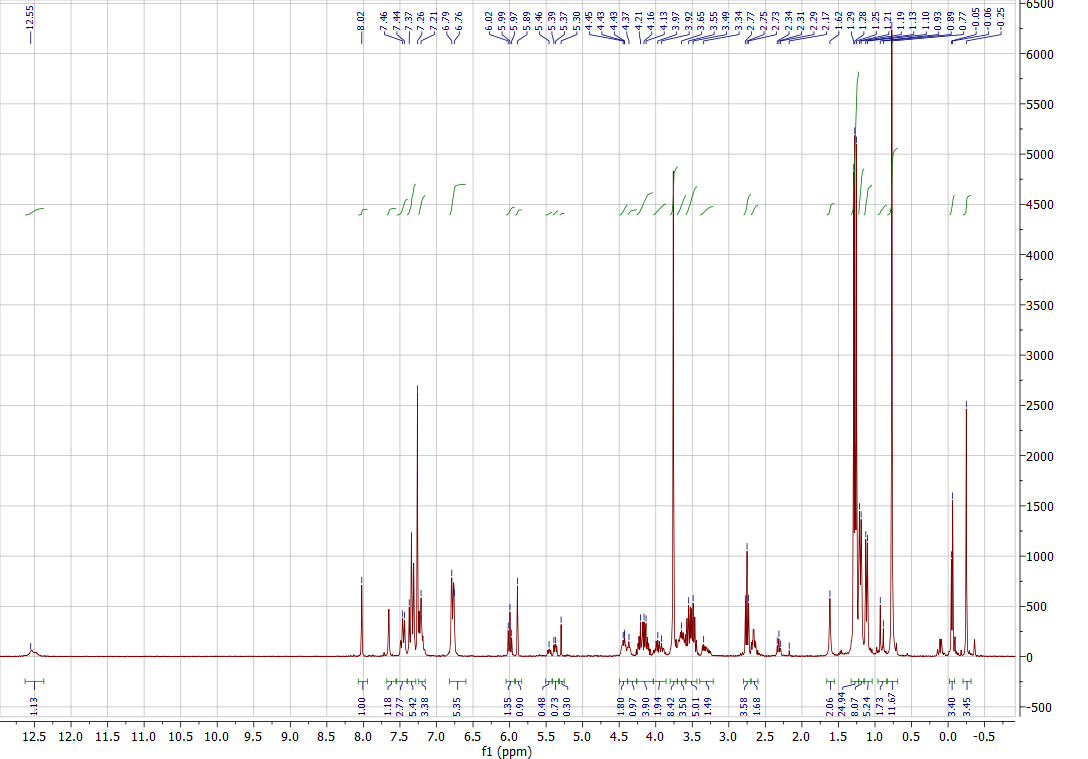


**Figure S8A:** ^1^HNMR spectrum of S8


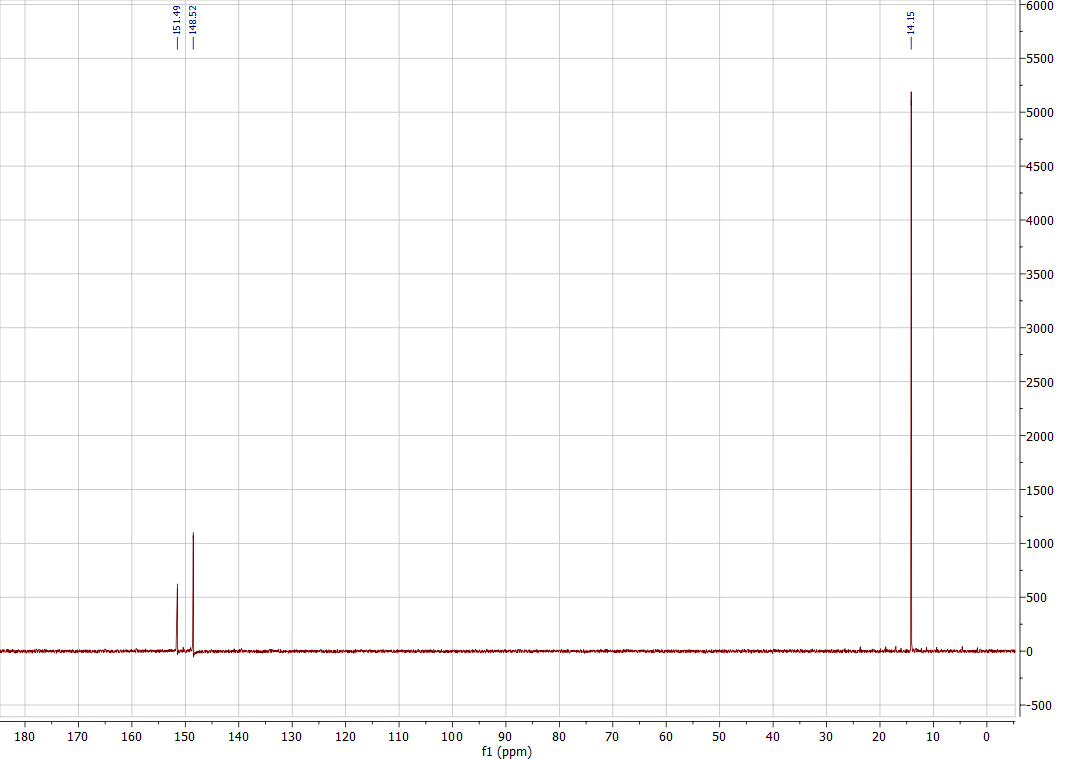


**Figure S8B:** ^31^PNMR spectrum of S8


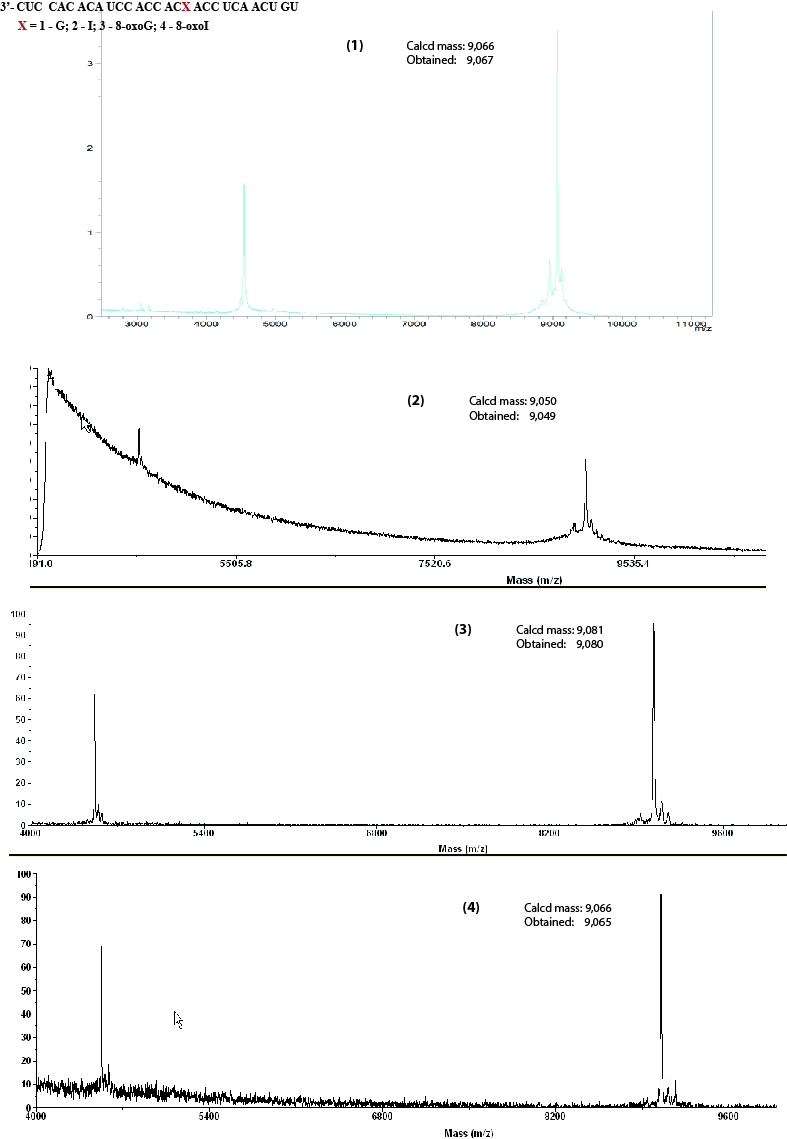


**Figure S9:** MALDI-TOF of oligonucleotides **1** - **4**

To obtain the spectra for oligonucleotide **9**, the sample was sent to the facilities at Colorado State University and the following was obtained, which justifies a difference of ~ 6 Da.

In addition, the additional peaks observed for samples **3** and **4** can be rationalized from decomposition upon shipping and/or handling. This is consistent with the appearance of one peak corresponding to the oligonucleotide of interest on samples taken elsewhere (Figure S9), which were spotted in our laboratory.

**Sample Preparation:**

The provided matrix components were combined in a 1:1 ratio to create 25mM 2,4,6-trihydroxyacetophenone

monohydrate, 10mM ammonium citrate, 300mM ammonium fluoride, 50% ACN. 20μl of 20μM (e.g. 20pmol/μl) sample

were provided. A total of 200pmol was used for zip tip clean up following instructions provided by MR: μC18 Zip Tips

(Millipore) were activated using 50% ACN (10μl x2) followed by equilibration in 0.1% TFA (10μl x2). 200pmol (e.g. 10μl)

of sample were then applied to the tip and loaded/aspirated for 10 cycles. The tip was then washed with 0.1% TFA (10μl

x2) and water (10μl x2) followed by elution into 10μl of matrix (10 cycles).

**Plate Spotting and Data Acquisition:**

1 μl of desalted eluate was spotted on the MALDI target and allowed to air dry. 1 μl of calibrant was spotted, allowed to

dry and then overlaid with 1 μl of α-Cyano-4-hydroxycinnamic acid (HCCA, 10 mg/ml in 50% ACN, 2.5% TFA). Molecular

weight measurement was performed on a Microflex-TOF mass spectrometer (Bruker Daltonics, Billerica, MA) in positive

ion, linear mode using an ion source voltage of 20 kV. External calibration was performed using a protein calibration

mixture (Protein Standard I, Bruker Daltonics) on a spot adjacent to the sample. The raw data was then processed in the

FlexAnalysis software (version 3.4, Bruker Daltonics).

Utilizing

high laser power combined with mis-matched matrix and molecule type appears to have introduced a systematic mass

error of ~6 Da.


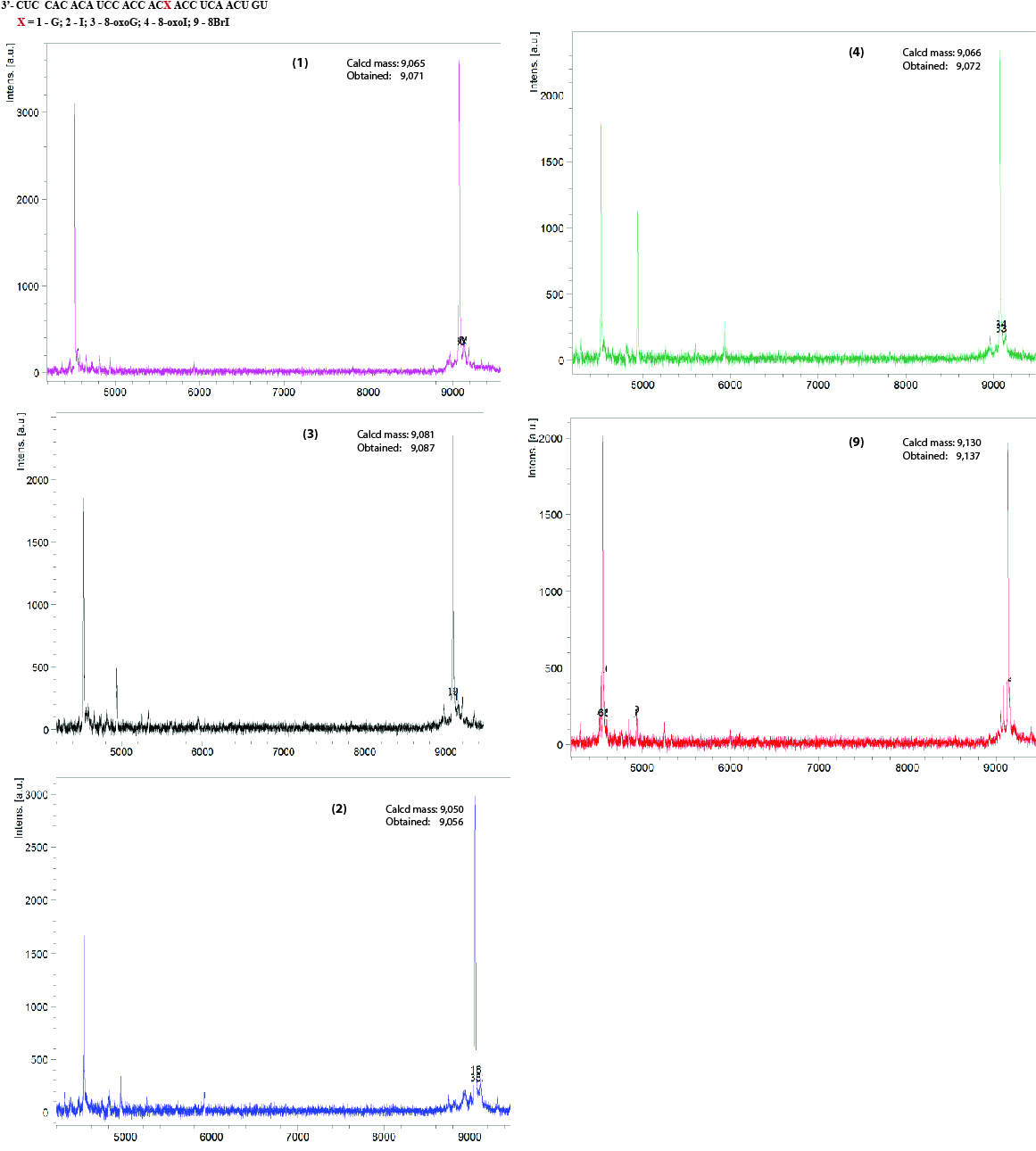


**Figure S9a:** MALDI-TOF of oligonucleotides **1** – **4 & 9**

**Figure S10:** Typical CD spectrum of annealed duplex at 20 °C and denatured duplex at 85 °C

**Experimental conditions:** 1.5 µM RNA and 2.0 µM complement DNA prepared in PBS (10 mM NaCl, 5 mM MgCl_2_, 1 mM Na_2_HPO_4_ pH 7.2)


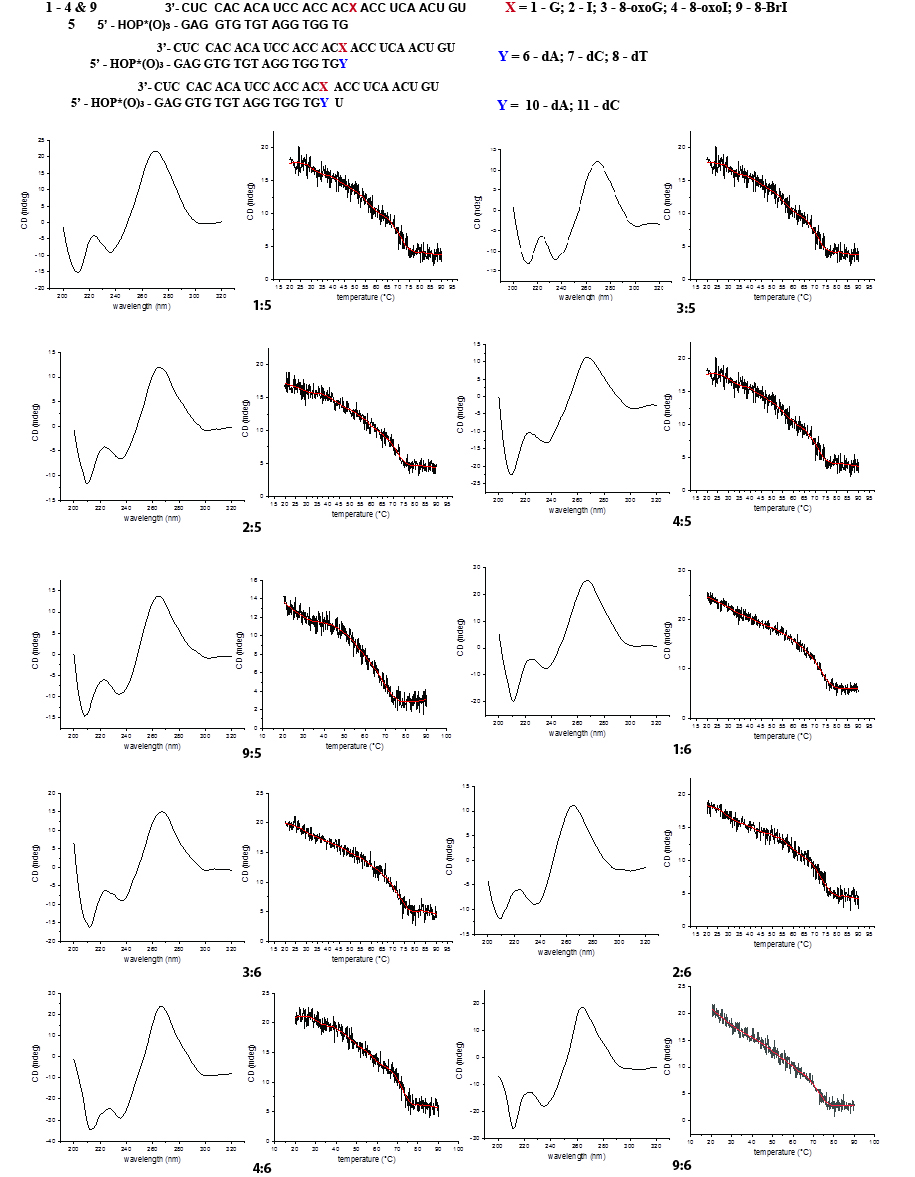
**Figure S11:** CD and T_m_ measurement for duplexes **1**:**5 -** **4**:**5;** **1**:**6-4:6;** and **9:5, 9:6**.


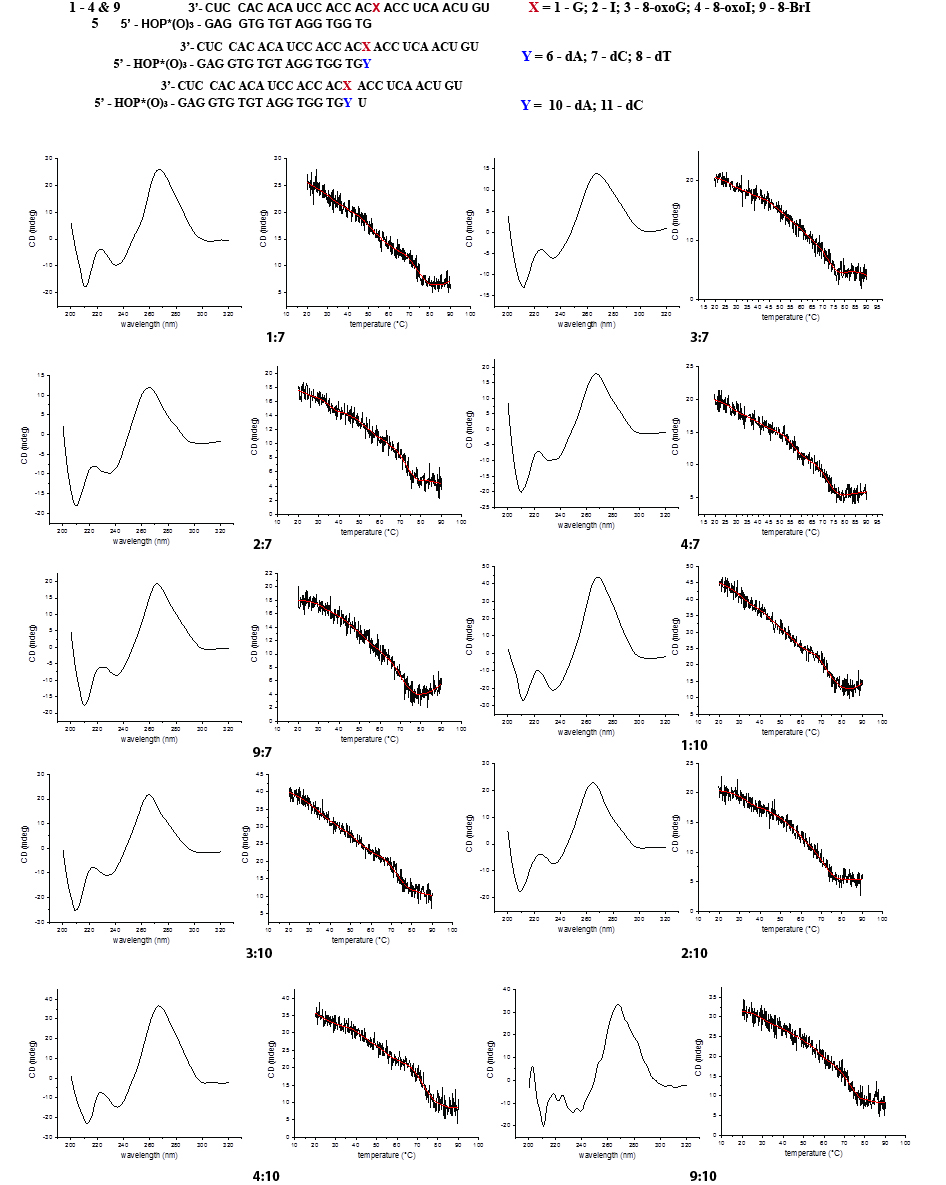


**Figure S12:** CD and T_m_ measurement for duplexes **1**:**7 -** **4**:**7;** **1**:**10-4:10;** and **9:7, 9:10**.


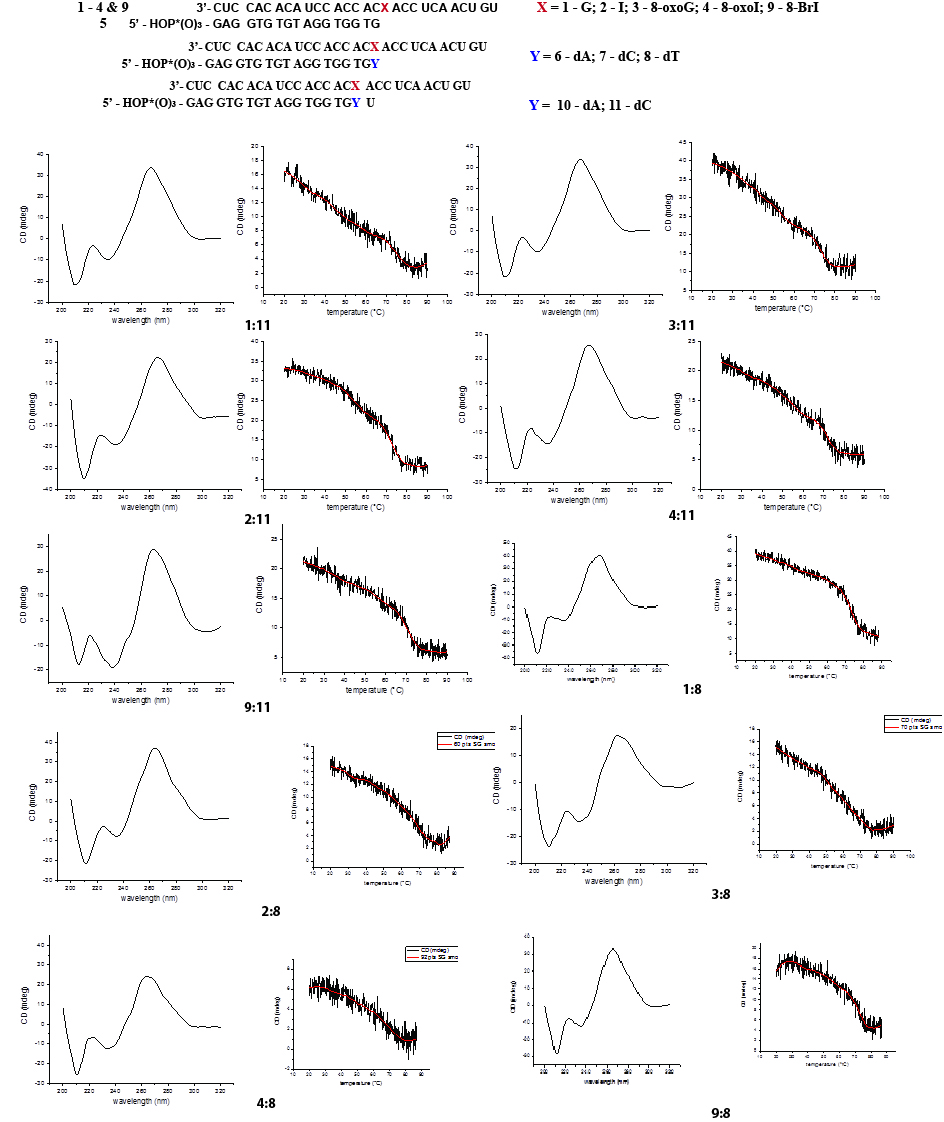


**Figure S13:** CD and T_m_ measurement for duplexes **1**:**11 -** **4**:**11;** **9:11**; **1**:**8 -** **4**:**8;** and **9:8**

**Figure S14:** Tables containing the values corresponding to T_m_ measurements carried out in triplicate. t1, t2, t3 stand for trials 1, 2, and 3; along with analysis of variance (ANOVA) for duplexes **1:5, 1:6. 1:7, 1:8, 1:10, 1:11.**

**Figure S15:** Tables containing the values corresponding to T_m_ measurements carried out in triplicate. t1, t2, t3 stand for trials 1, 2, and 3; along with analysis of variance (ANOVA) for duplexes **2:5, 2:6. 2:7, 2:8, 2:10, 2:11.**

**Figure S16:** Tables containing the values corresponding to T_m_ measurements carried out in triplicate. t1, t2, t3 stand for trials 1, 2, and 3; along with analysis of variance (ANOVA) for duplexes **3:5, 3:6. 3:7, 3:8, 3:10, 3:11.**

**Figure S17:** Tables containing the values corresponding to T_m_ measurements carried out in triplicate. t1, t2, t3 stand for trials 1, 2, and 3; along with analysis of variance (ANOVA) for duplexes **4:5, 4:6. 4:7, 4:8, 4:10, 4:11.**

**Figure S18:** Tables containing the values corresponding to T_m_ measurements carried out in triplicate. t1, t2, t3 stand for trials 1, 2, and 3; along with analysis of variance (ANOVA) for duplexes **9:5, 9:6. 9:7, 9:8, 9:10, 9:11.**


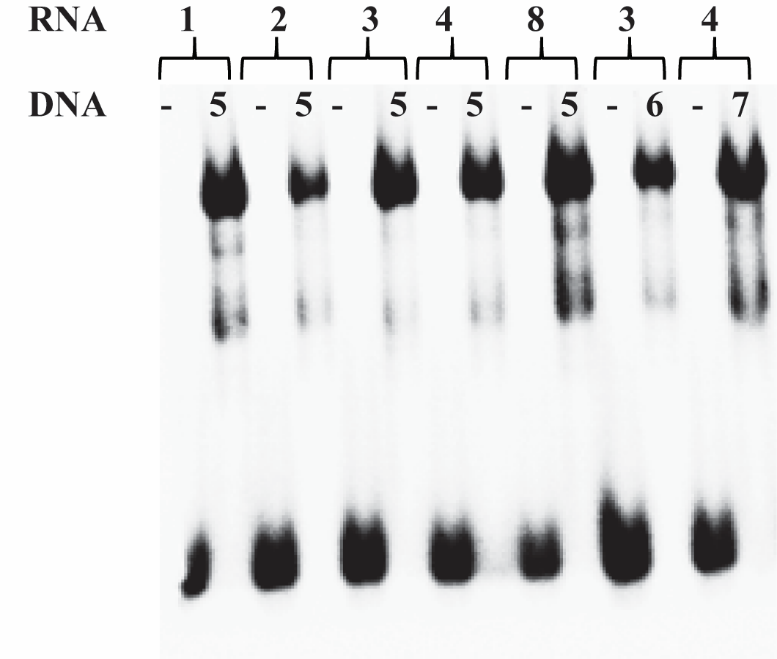


**Figure S19:** Native PAGE (20 %) of RNA w/wo DNA, showing duplex formation in the MMLV buffer (as described in the experimental section).


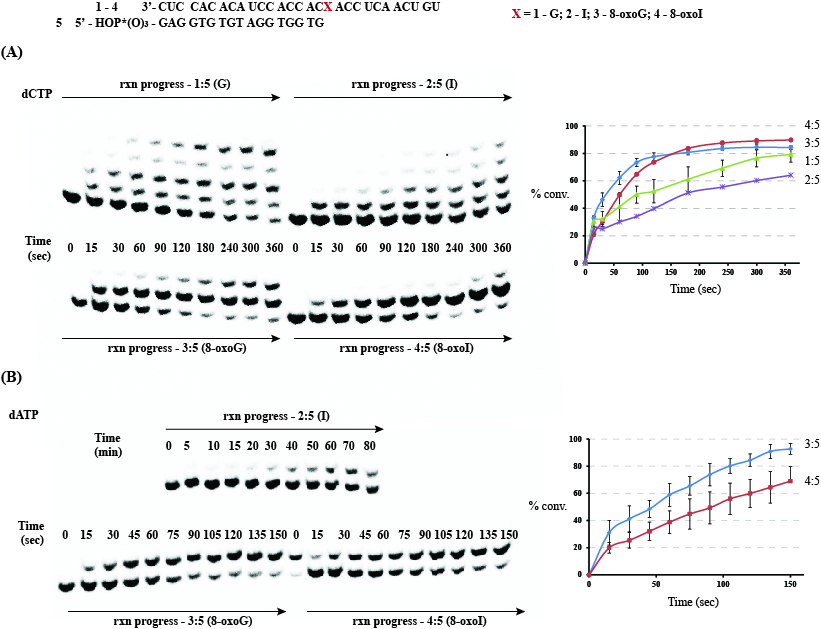


**Figure S20:** Relative rates for **1**:**5** – **4**:**5** with dCTP and **2**:**5** – **4**:**5** with dATP at constant [dNTP] and [AMV-RT] as a function of time. Reactions carried out at rt.


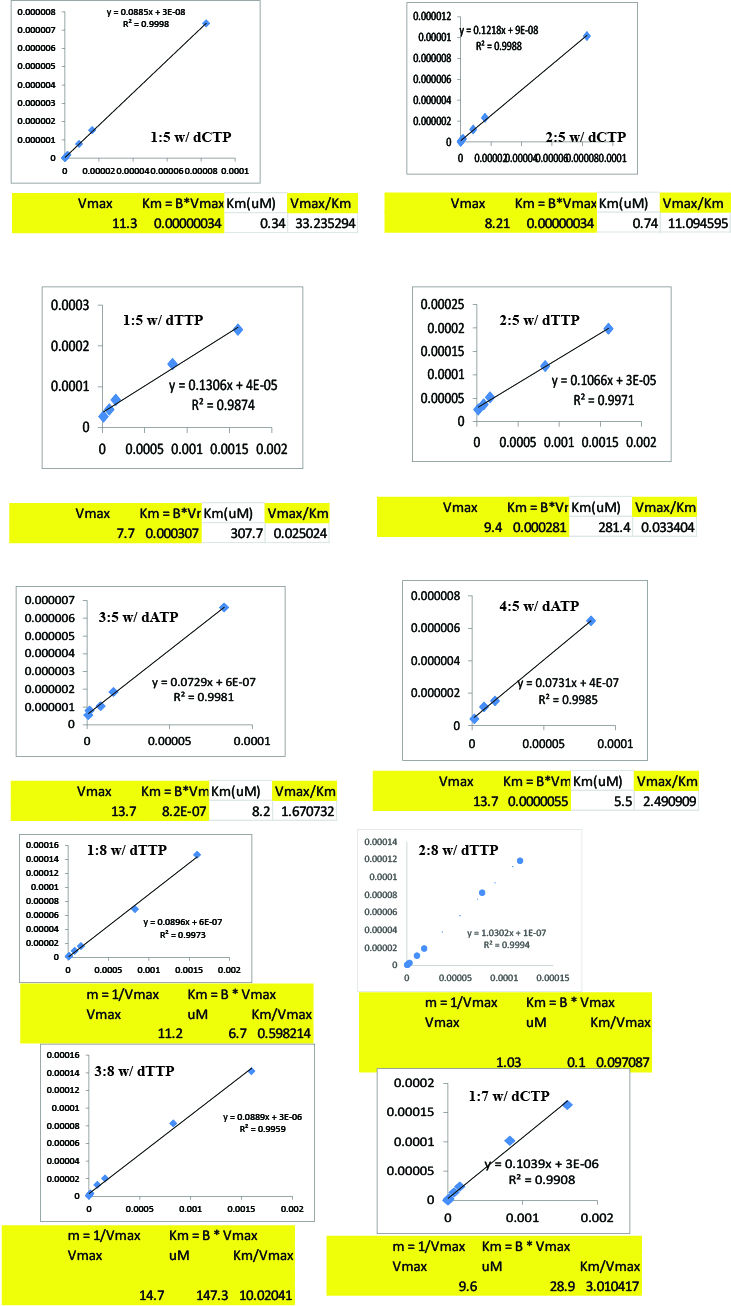


**Figure S21:** Sample single experiments for steady state kinetics Hanes-Woolf plots.


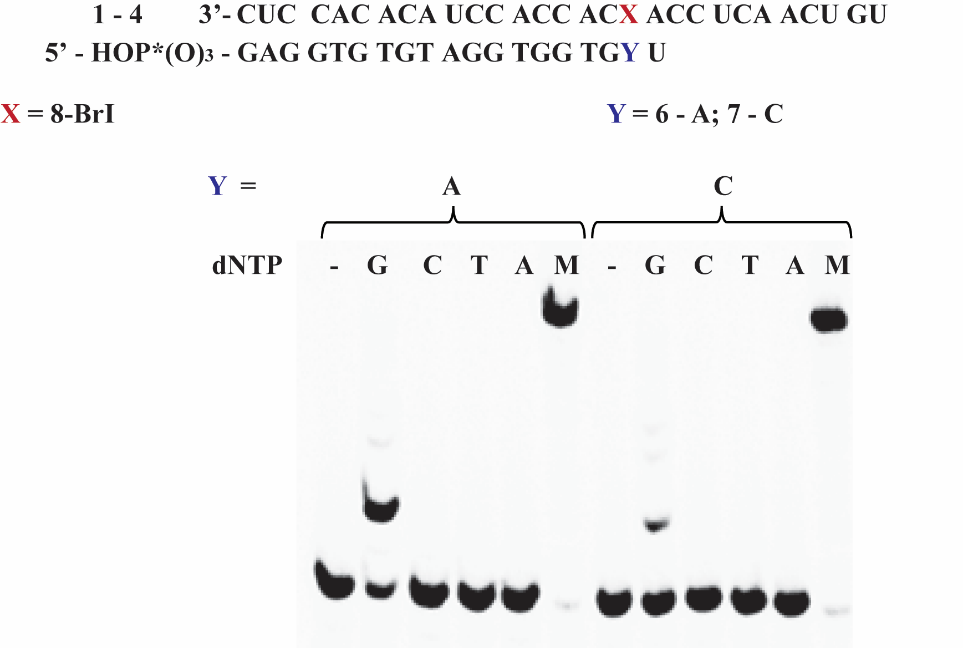


**Figure S22:** Duplexes **9**:**6** and **9**:**7** in the presence of AMV-RT.


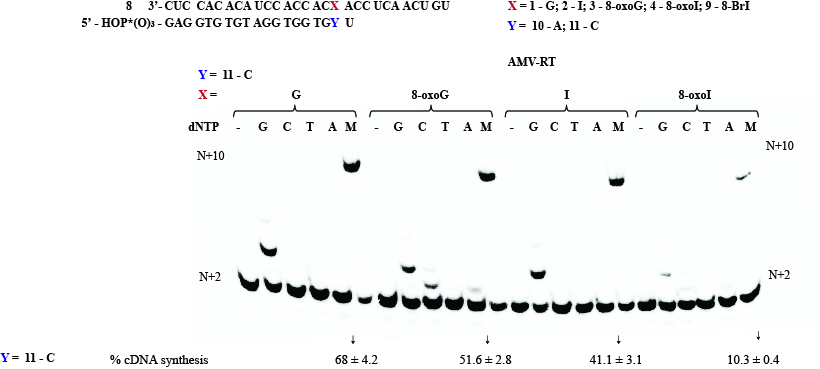


**Figure S23:** Duplexes **1**:**11** - **4**:**11** in the presence of AMV-RT.


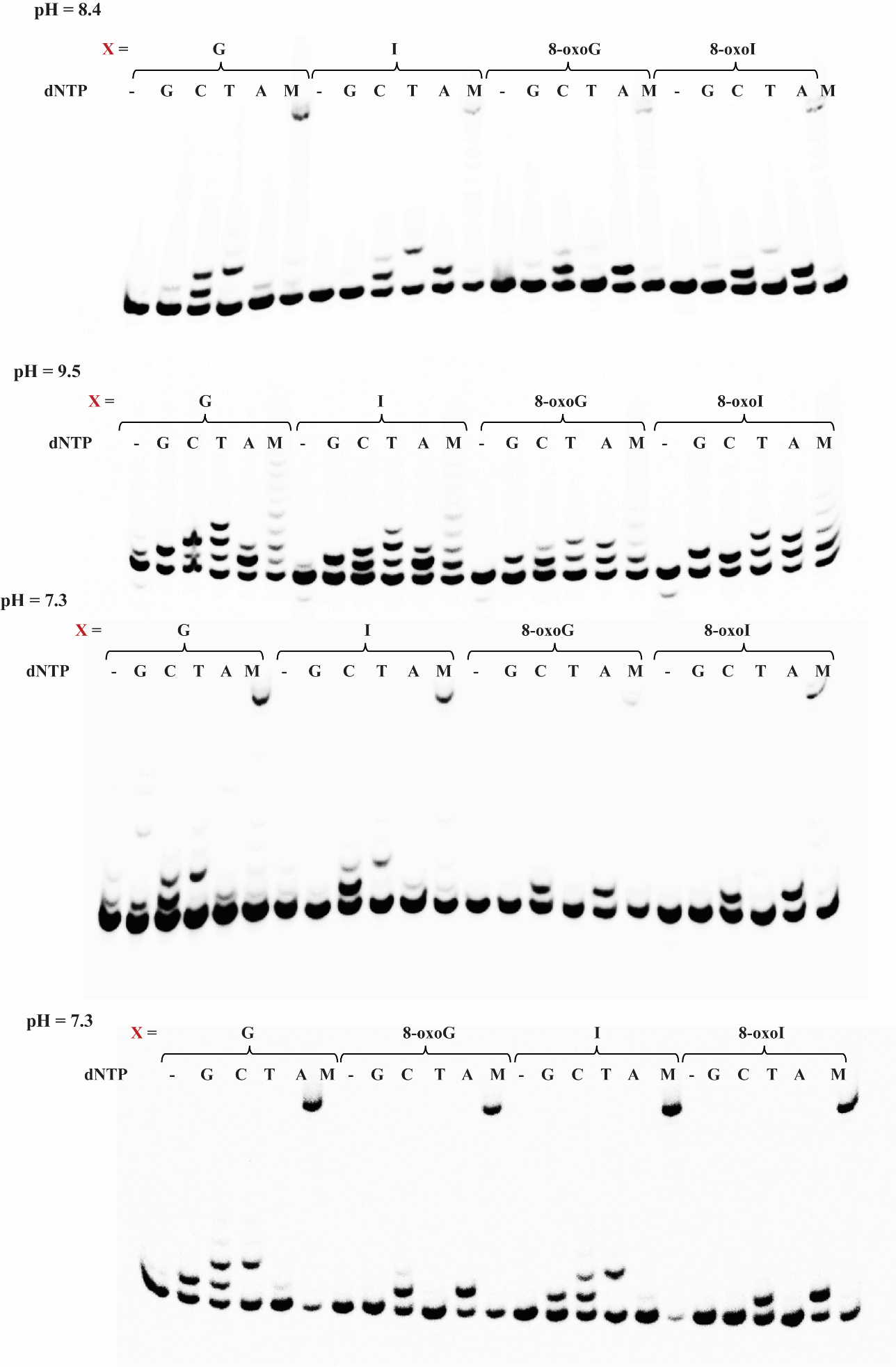


**Figure S24:** Duplexes **1**:**5** - **4**:**5** in the presence of AMV-RT at various pH values. Top three experiments represent incubation times of 5 min and the bottom experiment represents incubation time of 40 min.

1-4 3’-CUC CAC ACA UCC ACC ACX ACC UCA ACU GU

5 5’-HOP*(O)_3_- GAG GTG TGT AGG TGG TG

**Figure S25:** RNA:DNA **1**:**5**-**4**:**5** at higher (top) and lower (bottom) [MMLV-RT], see table in experimental section for description.

1-4 3’-CUC CAC ACA UCC ACC ACX ACC UCA ACU GU

5’-HOP*(O)_3_- GAG GTG TGT AGG TGG TGY

**Figure S26:**  RNA:DNA **1**:**6**-**4**:**6** & **1**:**7**-**4**:**7** at lower [MMLV].

1-4 3’-CUC CAC ACA UCC ACC ACX ACC UCA ACU GU

5 5’-HOP*(O)_3_- GAG GTG TGT AGG TGG TG

**Figure S27:** RNA:DNA **1**:**5**-**4**:**5** using SSII (Superscript II).


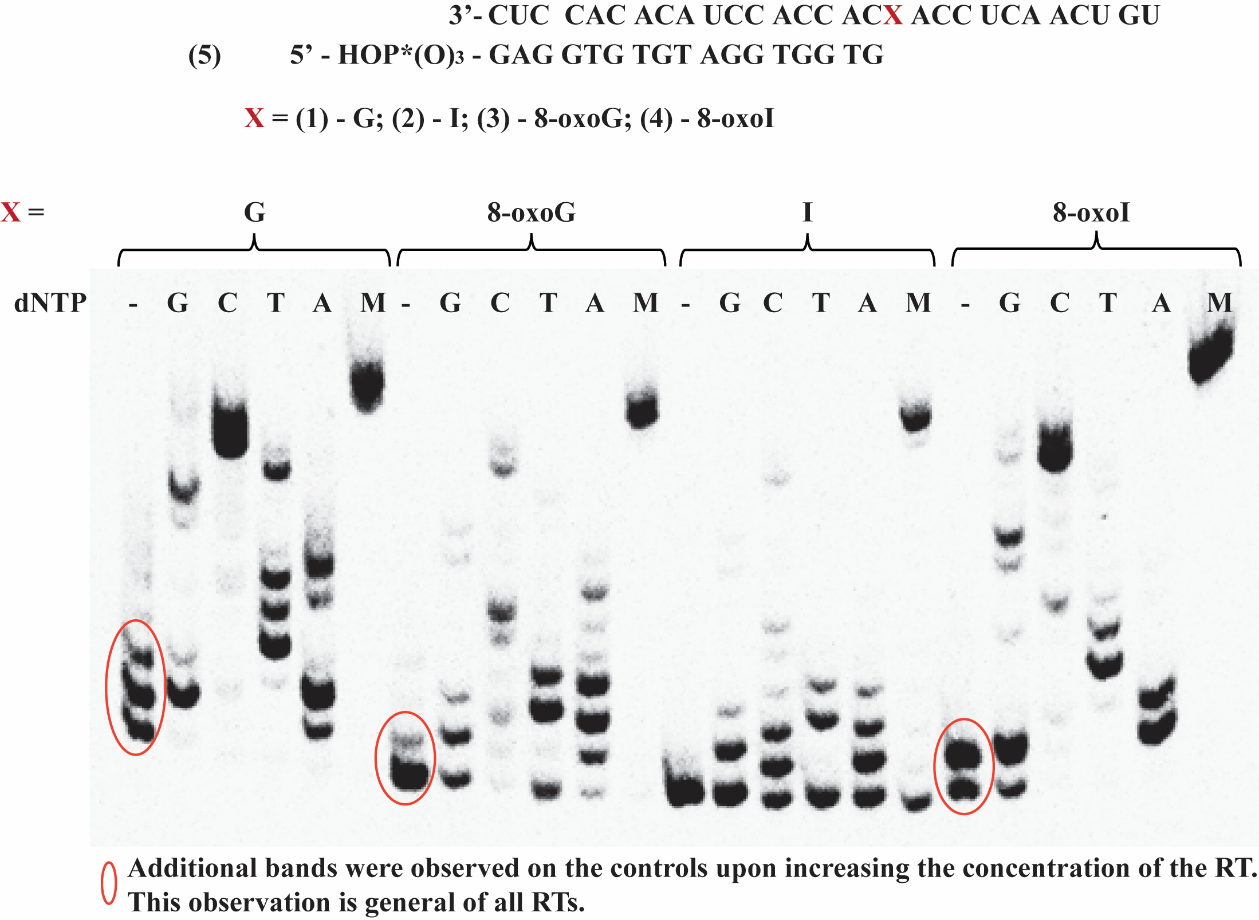


**Figure S28:** Duplexes **1**:**5** – **4**:**5** in the presence of higher [HIV-RT]. HIV units used were ca. 0.187 per well, 30 times more than the experiments described on the manuscript.


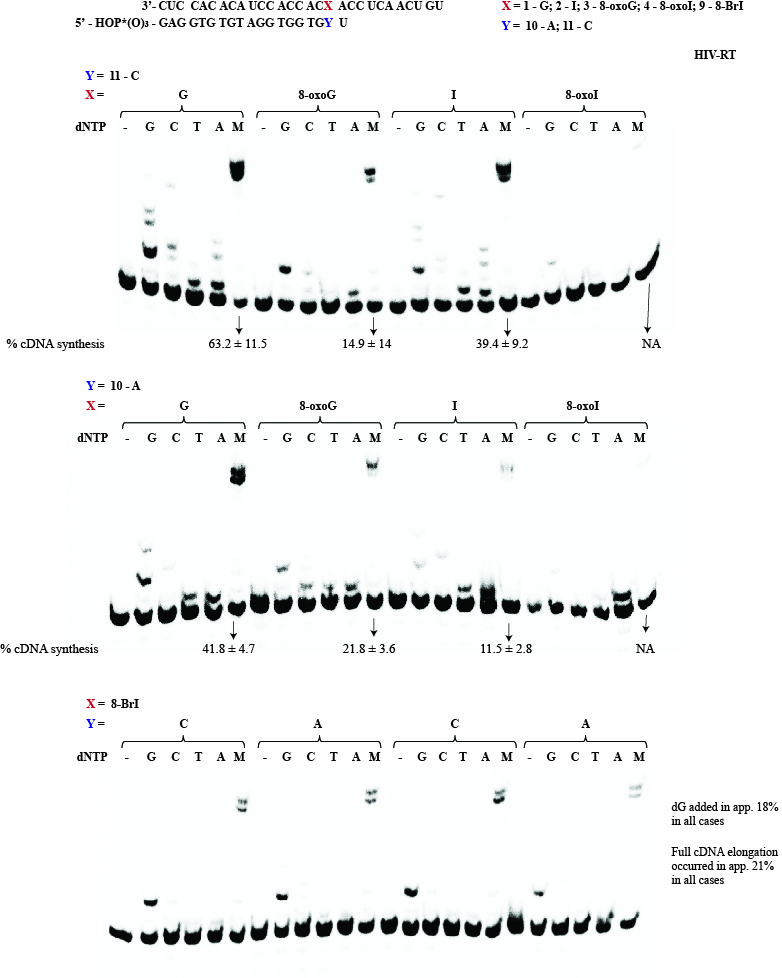


**Figure S29:** Duplexes **1**:**10** – **4**:**10**, **1**:**11** – **4**:**11** and **9**:**10** / **9**:**11** in the presence of HIV-RT


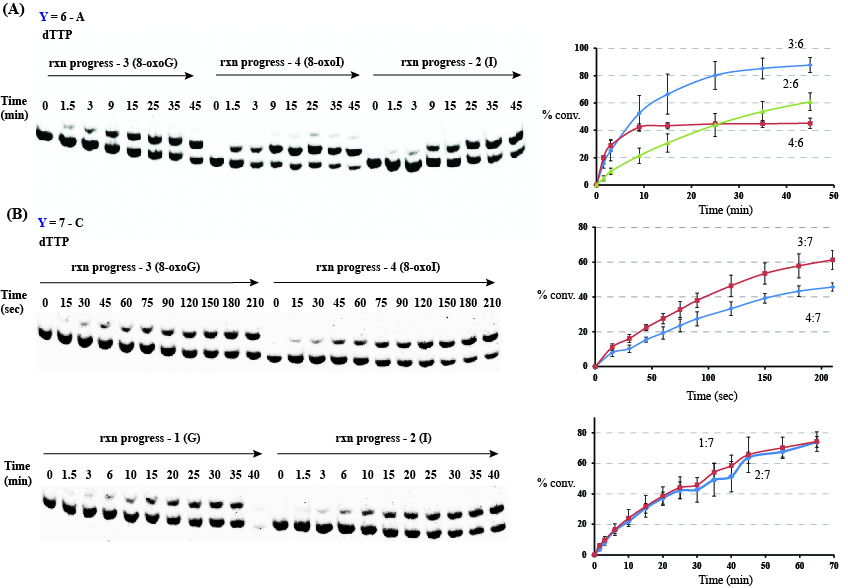


**Figure S30:** Relative rates for **2**:**6** – **4**:**6** with dTTP and **1**:**7** – **4**:**7** with dTTP at constant [dNTP] and [AMV-RT] as a function of time. Reactions carried out at rt.


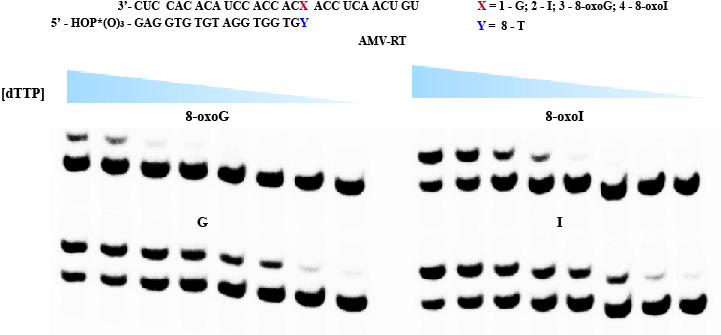


**Figure S31:** Steady state kinetics sample gel for **1**:**8** – **4**:**8** with dTTP at decreasing [dNTP] and constant [AMV-RT] with incubation at 38 °C for 5 min.

**References**

(1) Green, R.; Szostak, J. W.; Benner, S. A.; Rich, A.; Usman, N. Synthesis of RNA containing inosine: analysis of the sequence requirements for the 5’ splice site of the *Tetrahymena* group I intron. *Nucleic Acids Res.* **1991**, 19, 4161-4166.

(2) Matulic-Adamic, J.; Beigelman, L. An improved synthesis of inosine 3’-phosphoramidite. *Syn. Commun.* **2000**, 30, 3963-3969.

(3) Lena, S.; Cremonini, M. A.; Federiconi, F.; Gottarelli, G.; Graziano, C.; Laghi, L.; Mariani, P.; Masiero, S.; Pieraccini, S.; Spada, G. P. The supramolecular helical architecture of 8-oxoinosine and 8-oxoguanosine derivatives. *Chem. Eur. J.* **2007**, 13, 3441-3449.

(4) Lin, T-S.; Cheng, J-C.; Ishiguro, K.; Sartorelli, A. C. Purine and 8-substituted purine arabinofuranosyl and ribofuranosyl nucleoside derivatives as potential inducers of the differentiation of the friend erythroleukemia. *J. Med. Chem.* **1985**, 28, 1481-1485.
