# Supplementary material for: Translesion synthesis by AMV, HIV, and MMLVreverse transcriptases using RNA templates containing inosine, guanosine, and their 8-oxo-7,8-dihydropurine derivatives
Source: PLoS One. 2020 Aug 28;15(8):e0235102. doi: 10.1371/journal.pone.0235102 (PMC7455023; doi:10.1371/journal.pone.0235102)
Supplement: S17 File — (PDF) [file pone.0235102.s017.pdf]

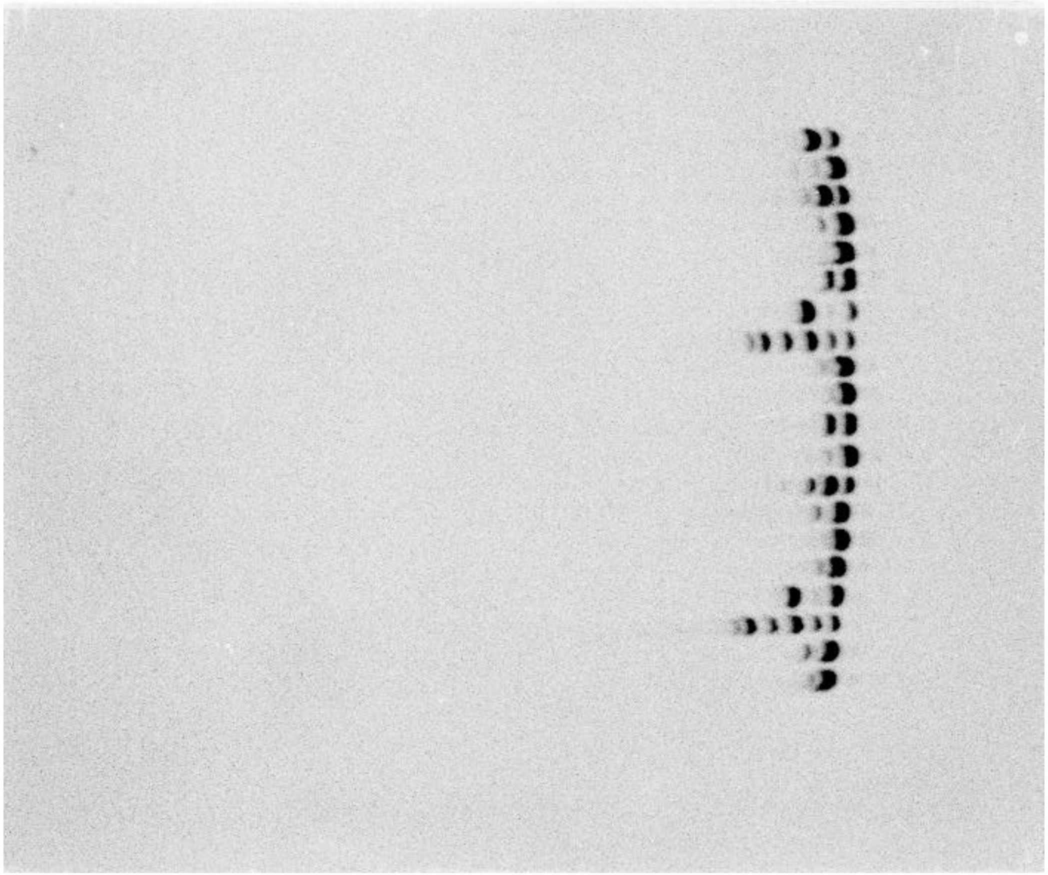

Figure 2B

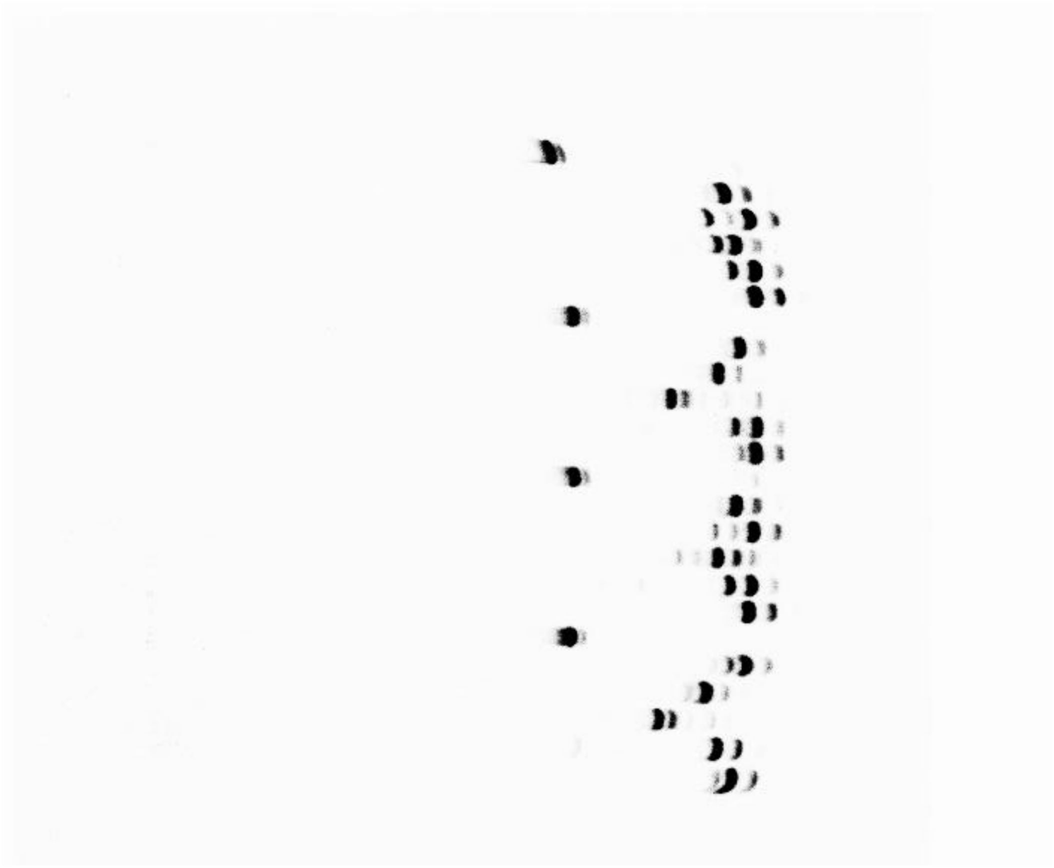

Figure 2D

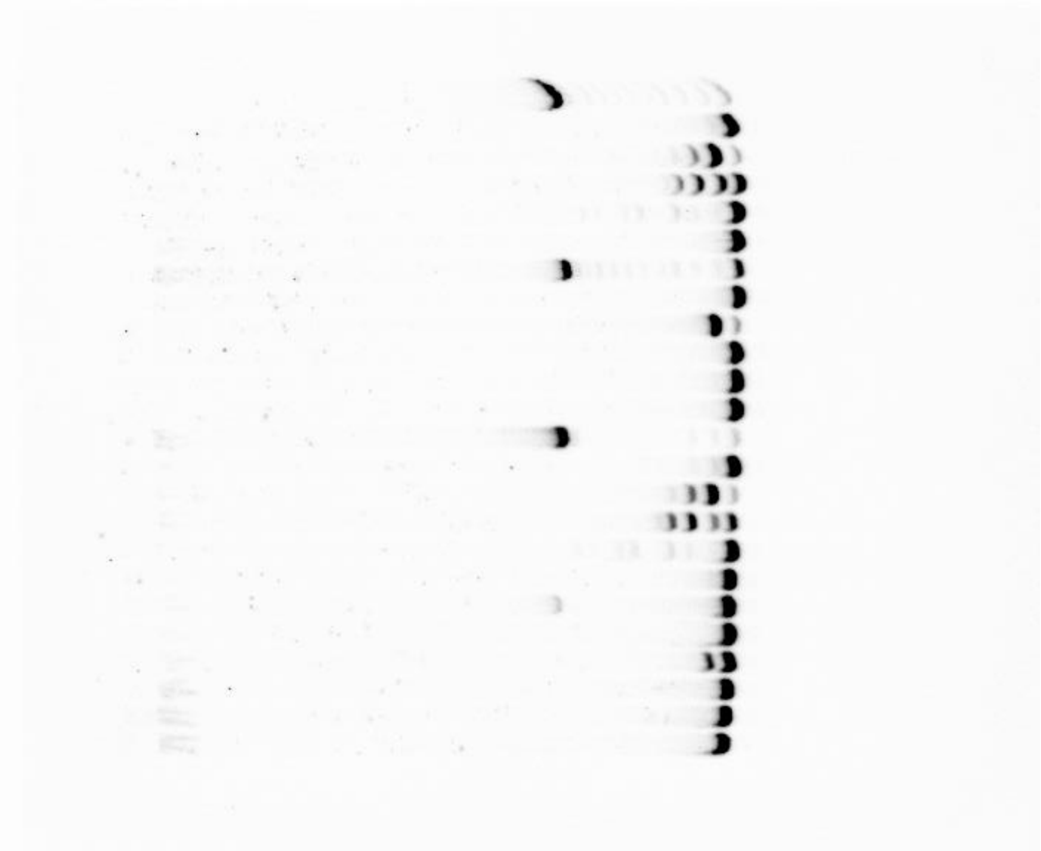

Figure 3B

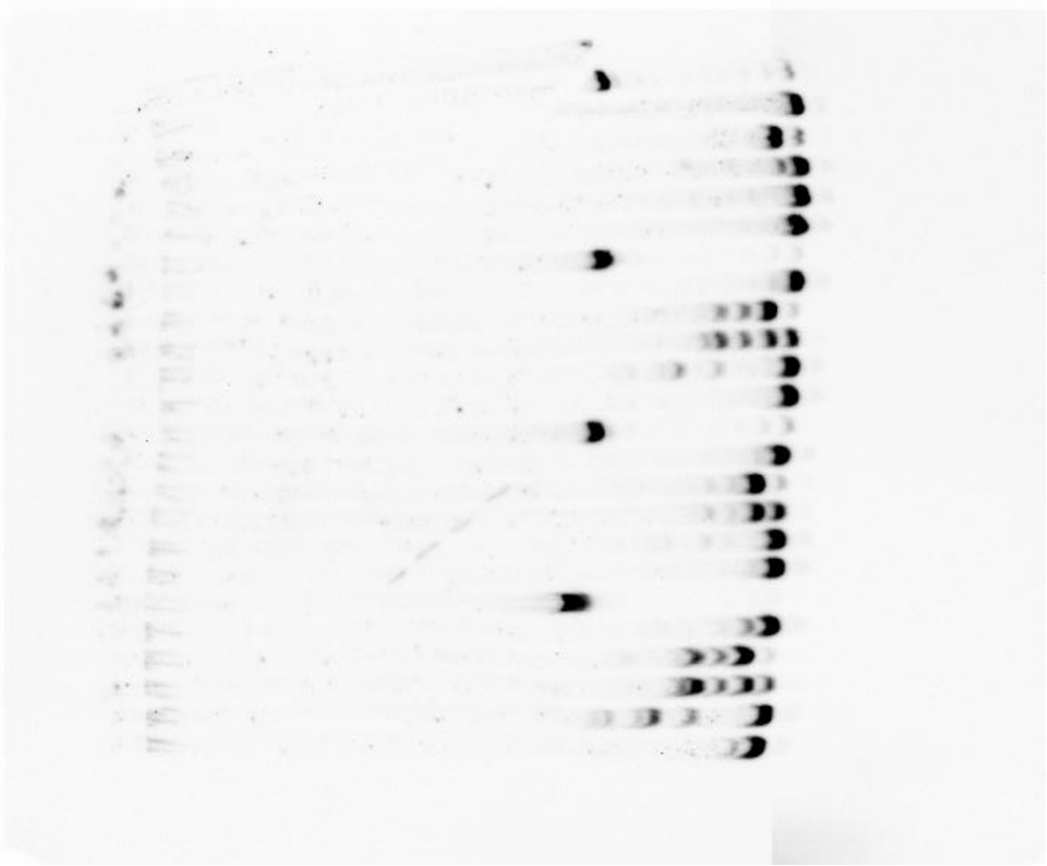

Figure 3C

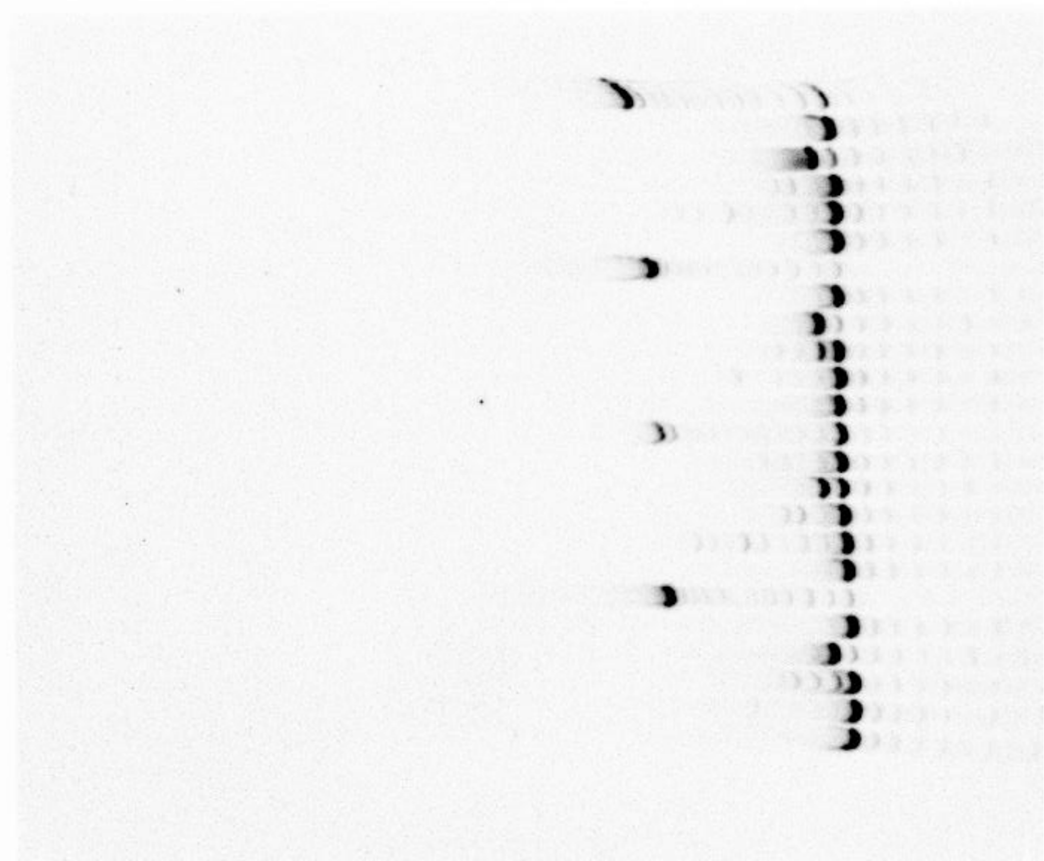

Figure 3D

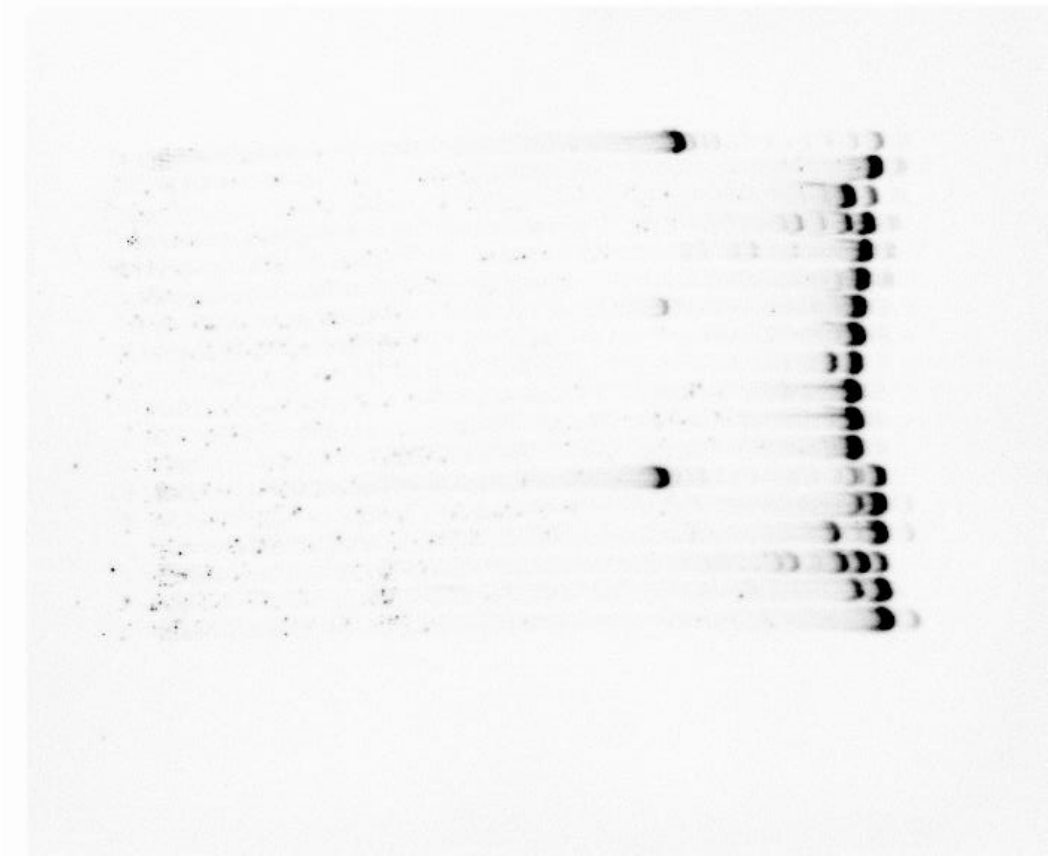

Figure 4B

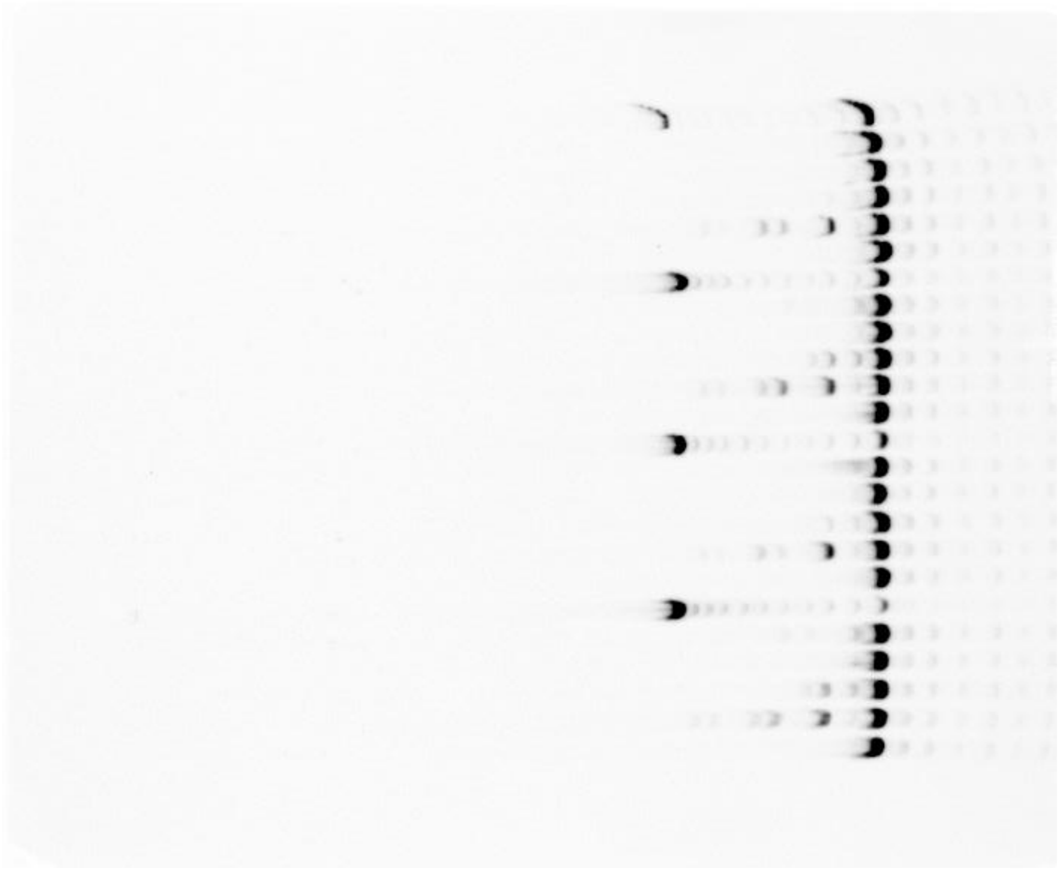

Figure 5A

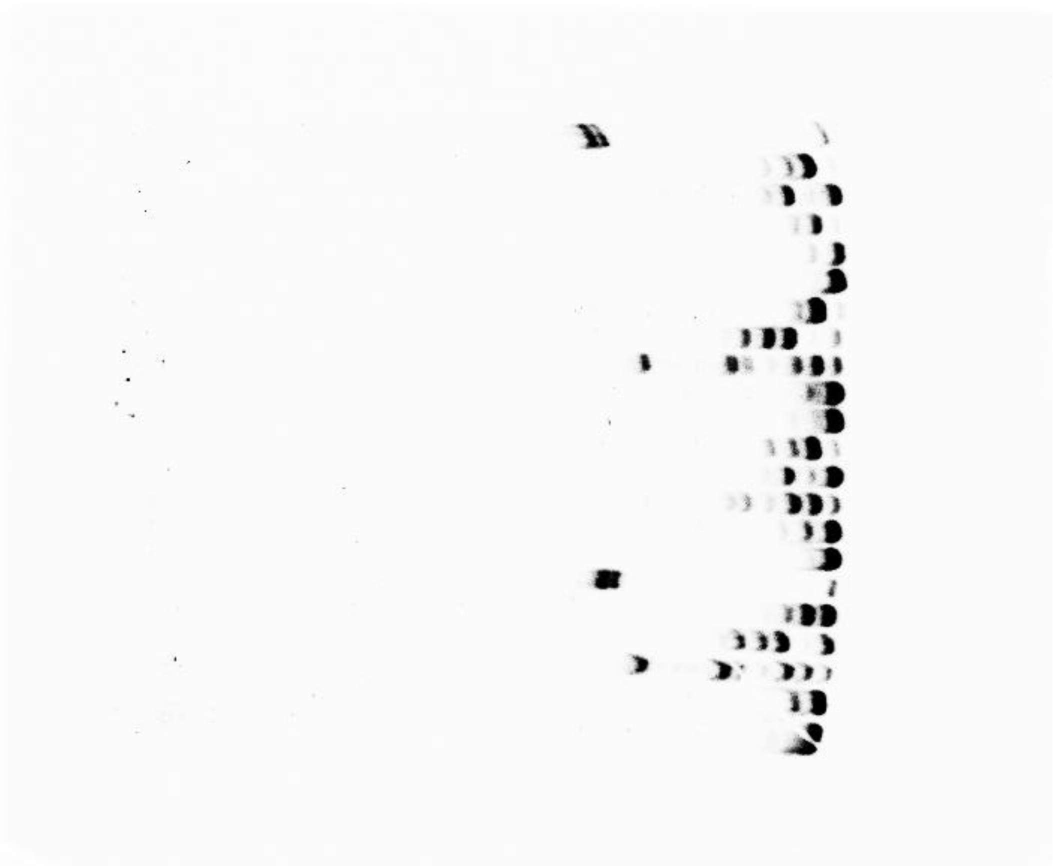

Figure 6

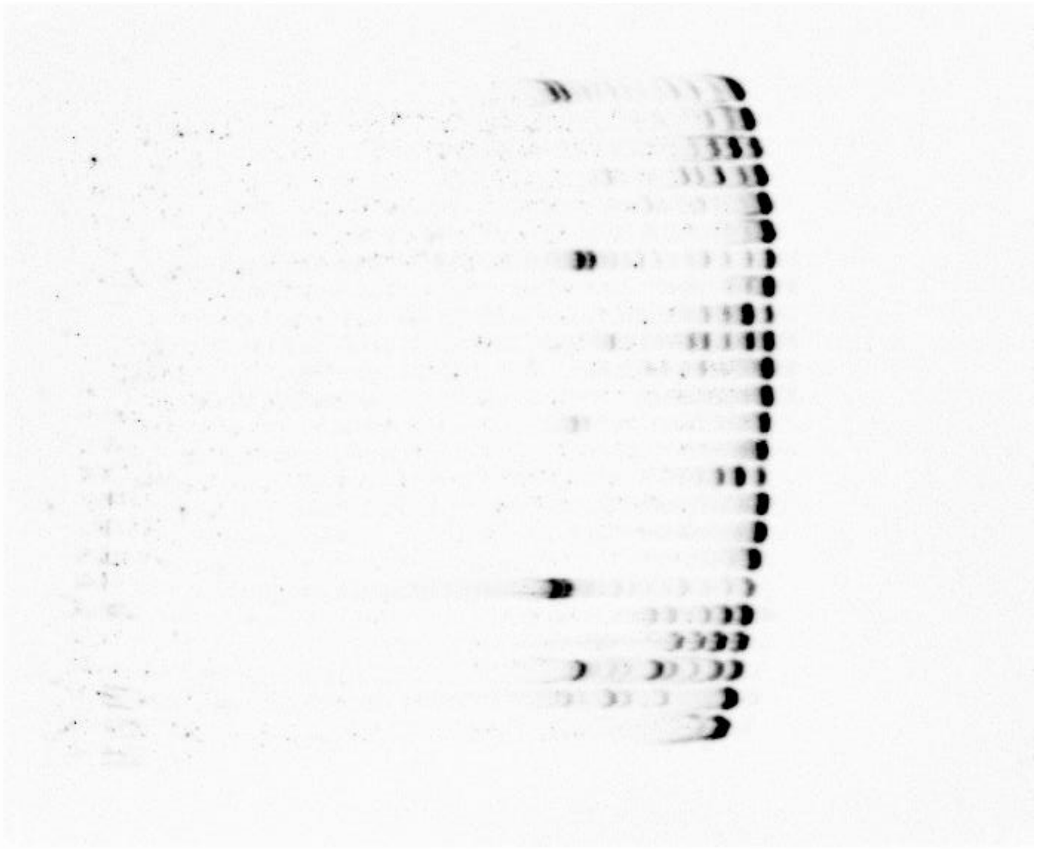

Figure 7A

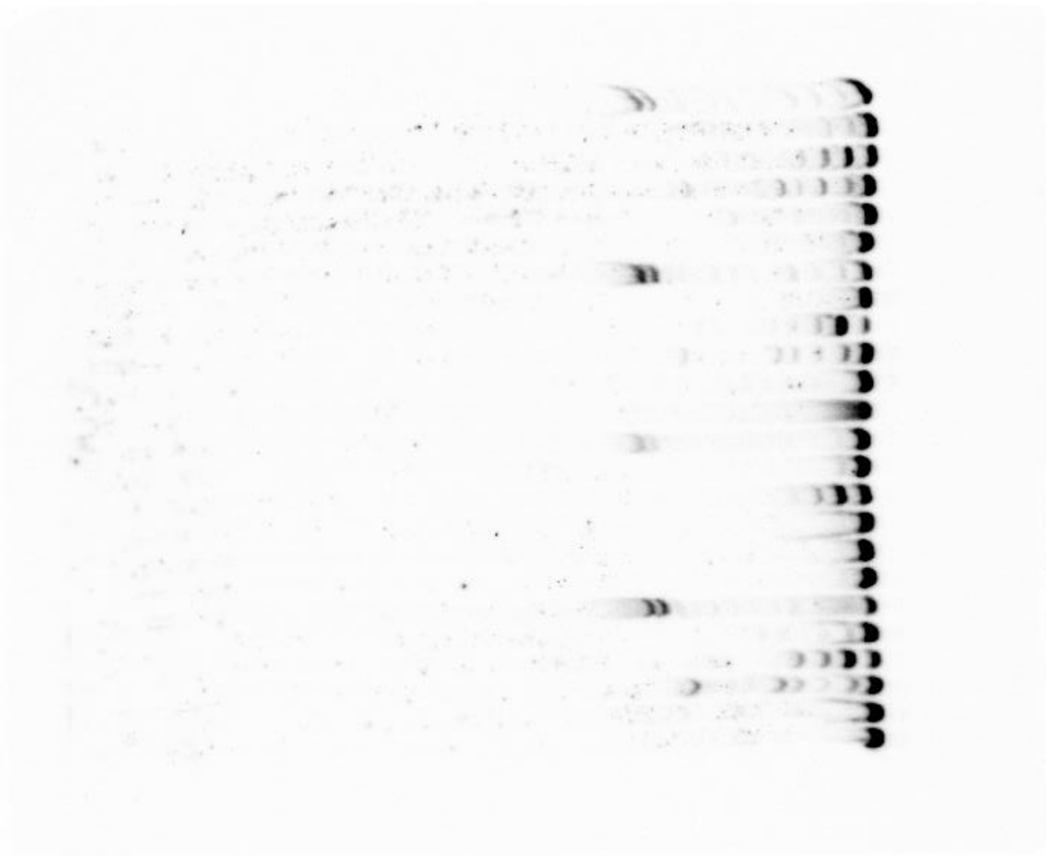

Figure 7B

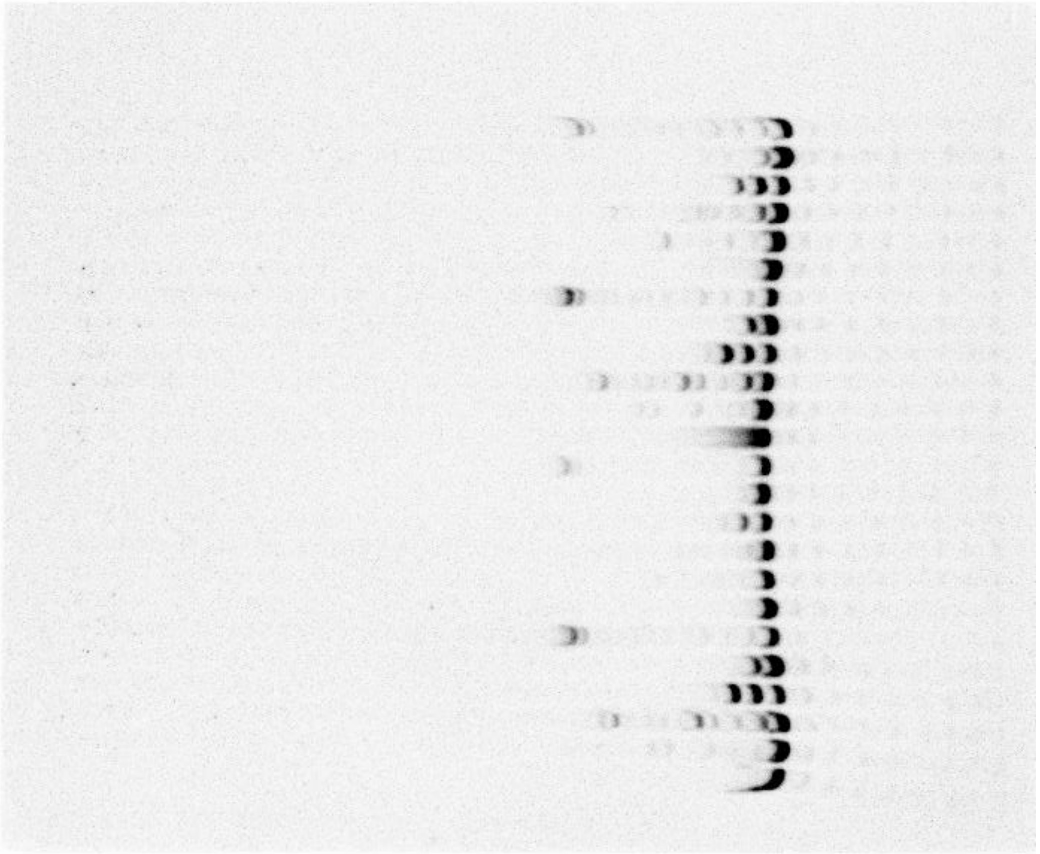

Figure 7C

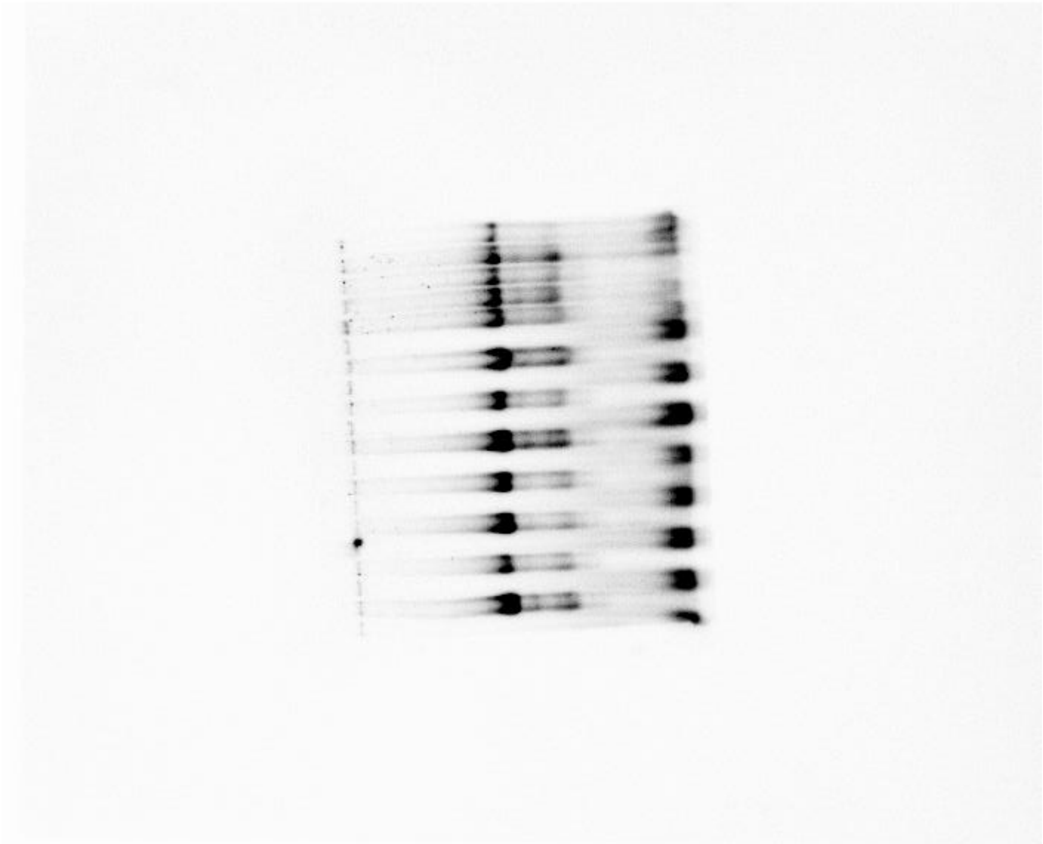

S6 File

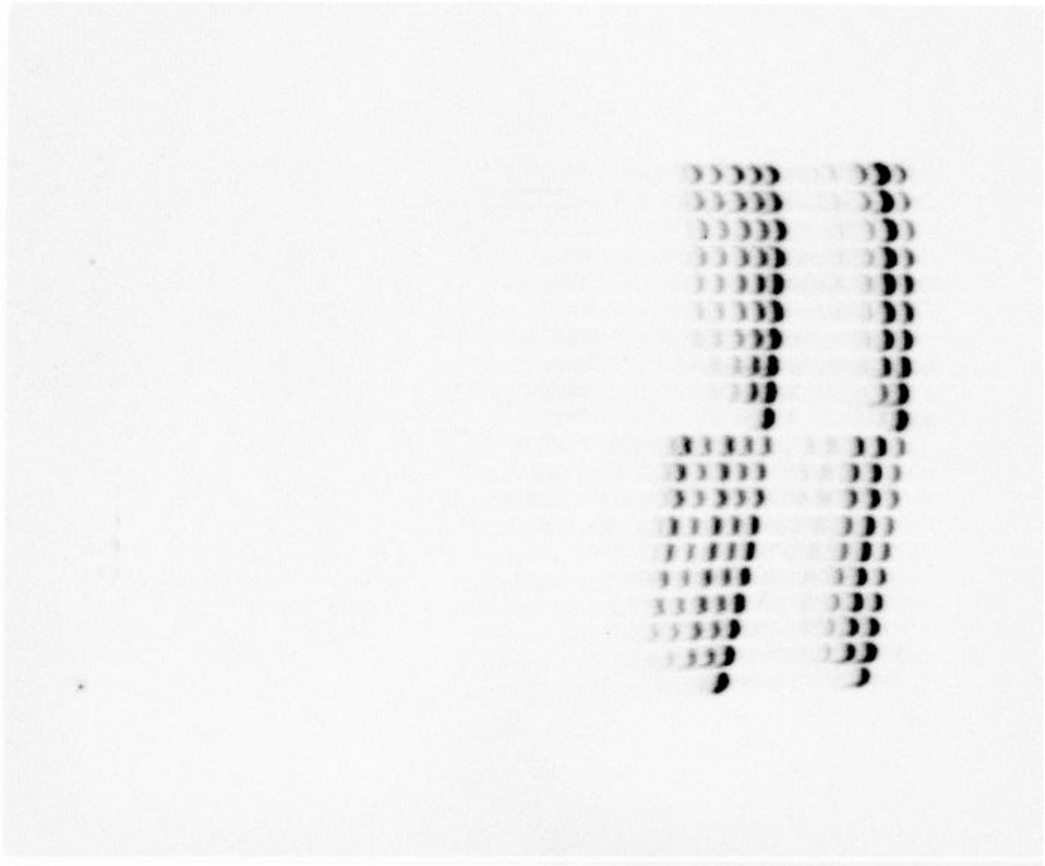

S7 FileA

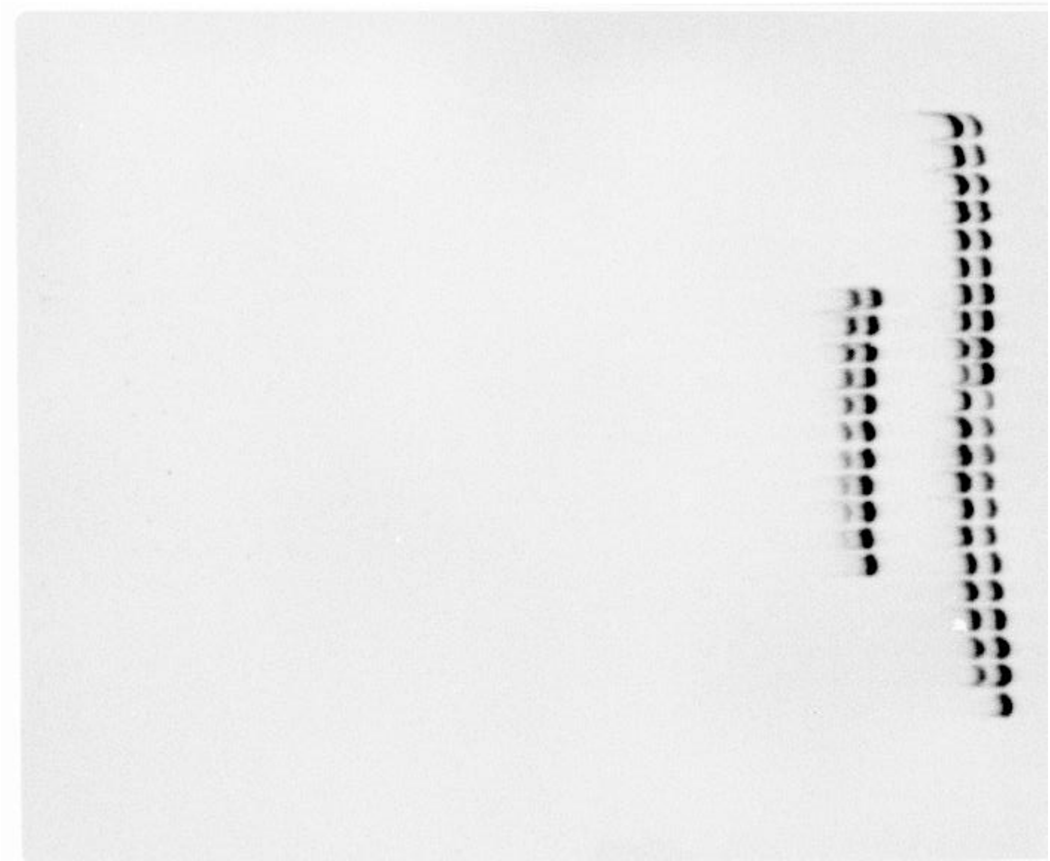

S7 File B

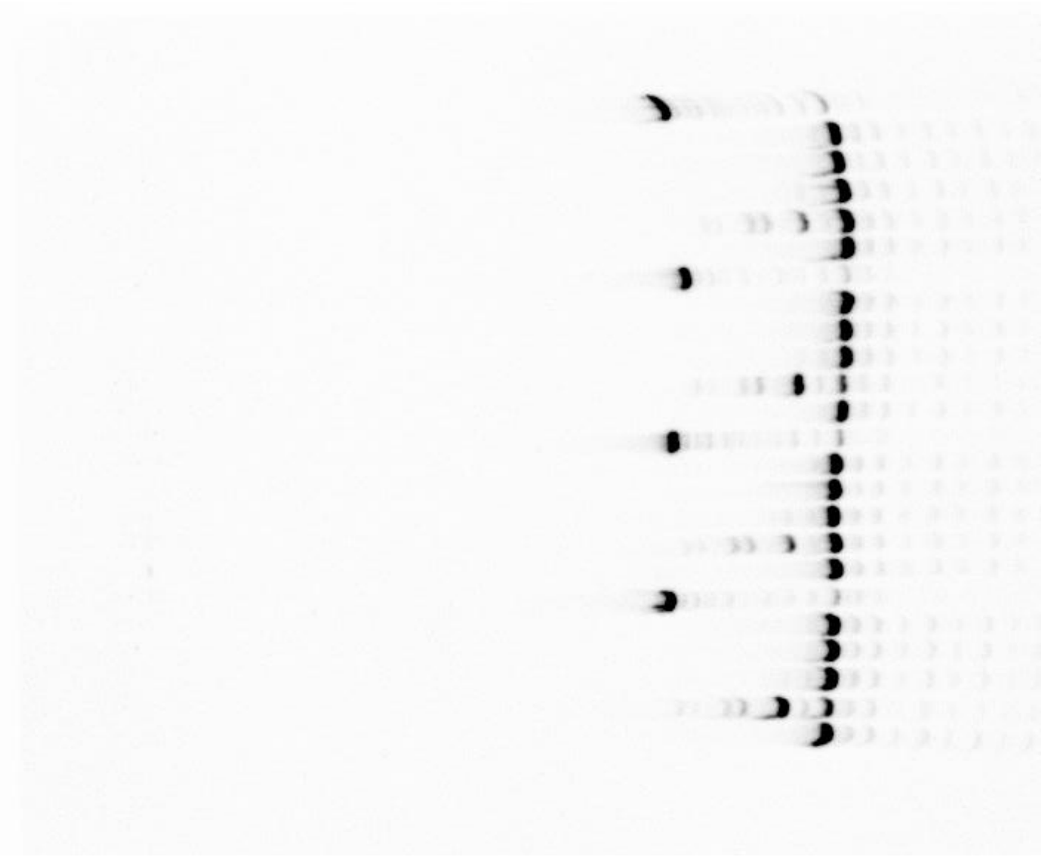

S8 File

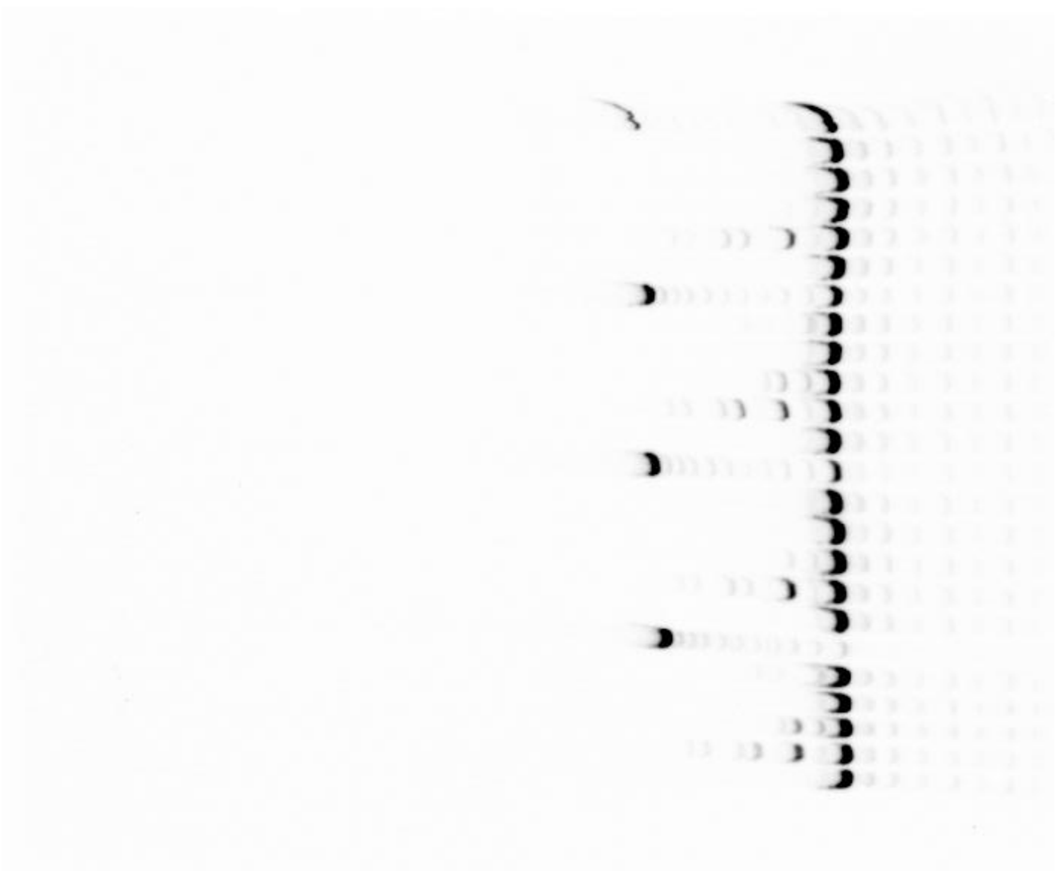

S9 File

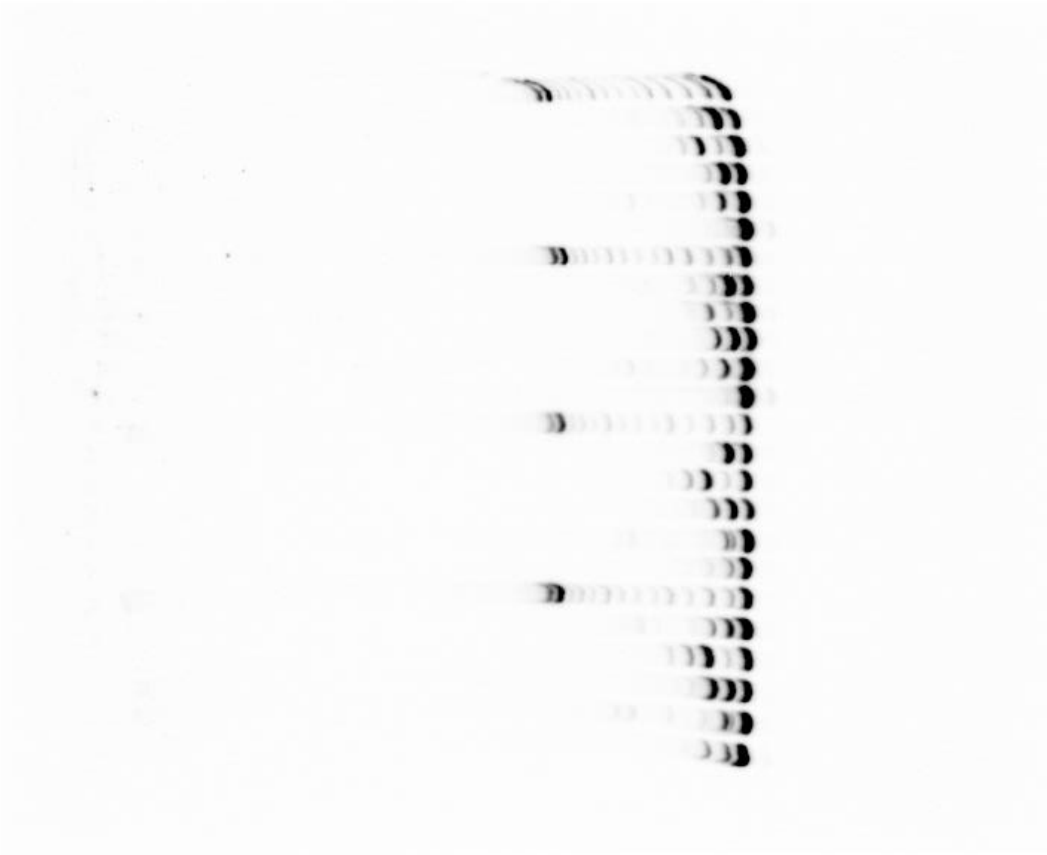

S10 File-top

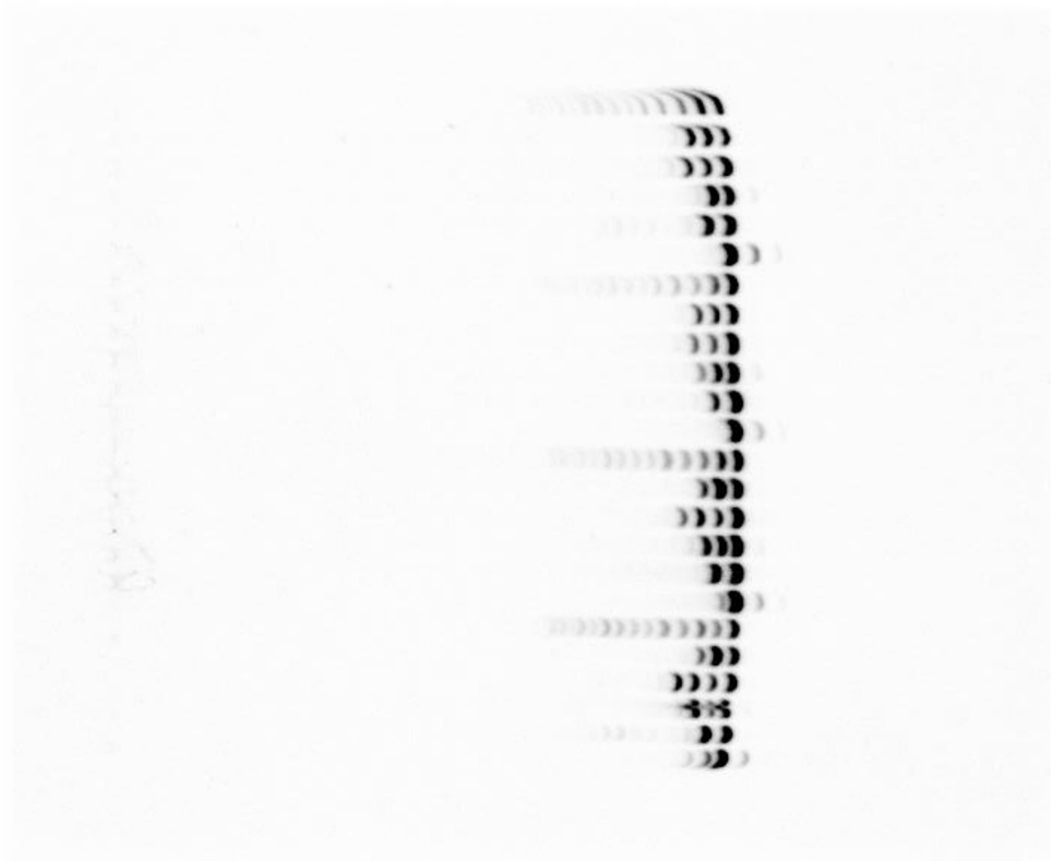

S10 File 2nd top, pH 7.3

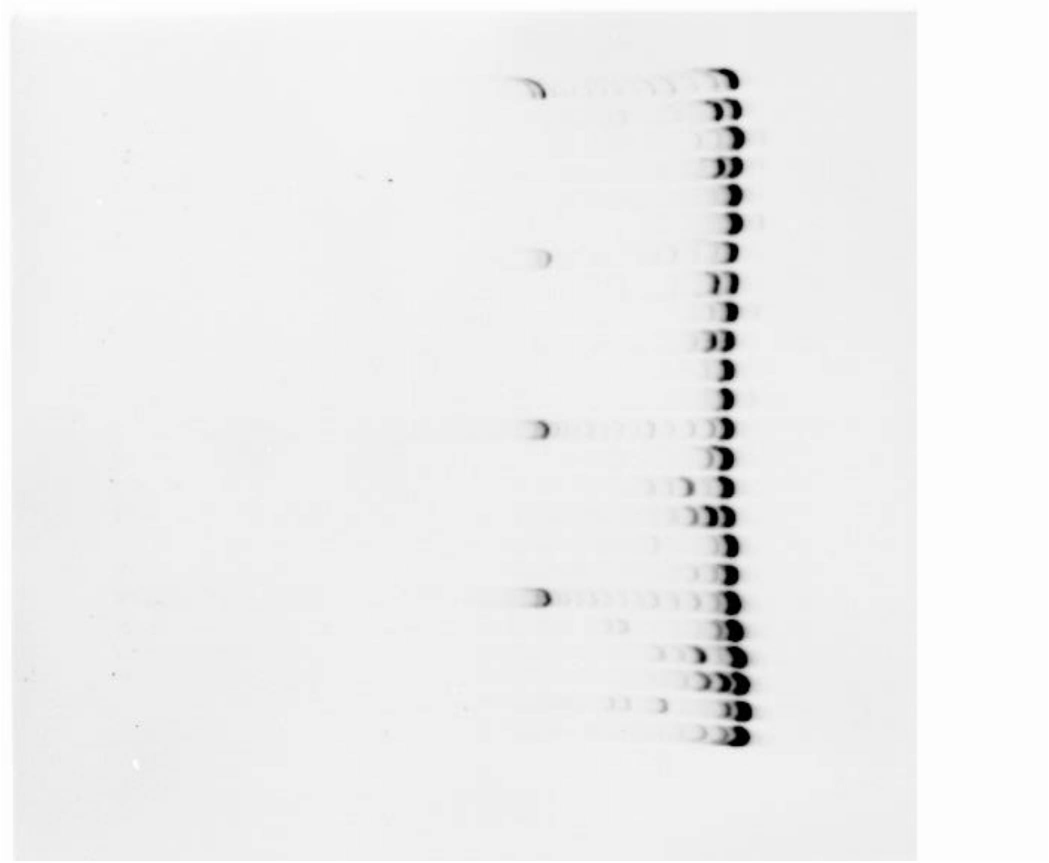

S10 File 3rd top

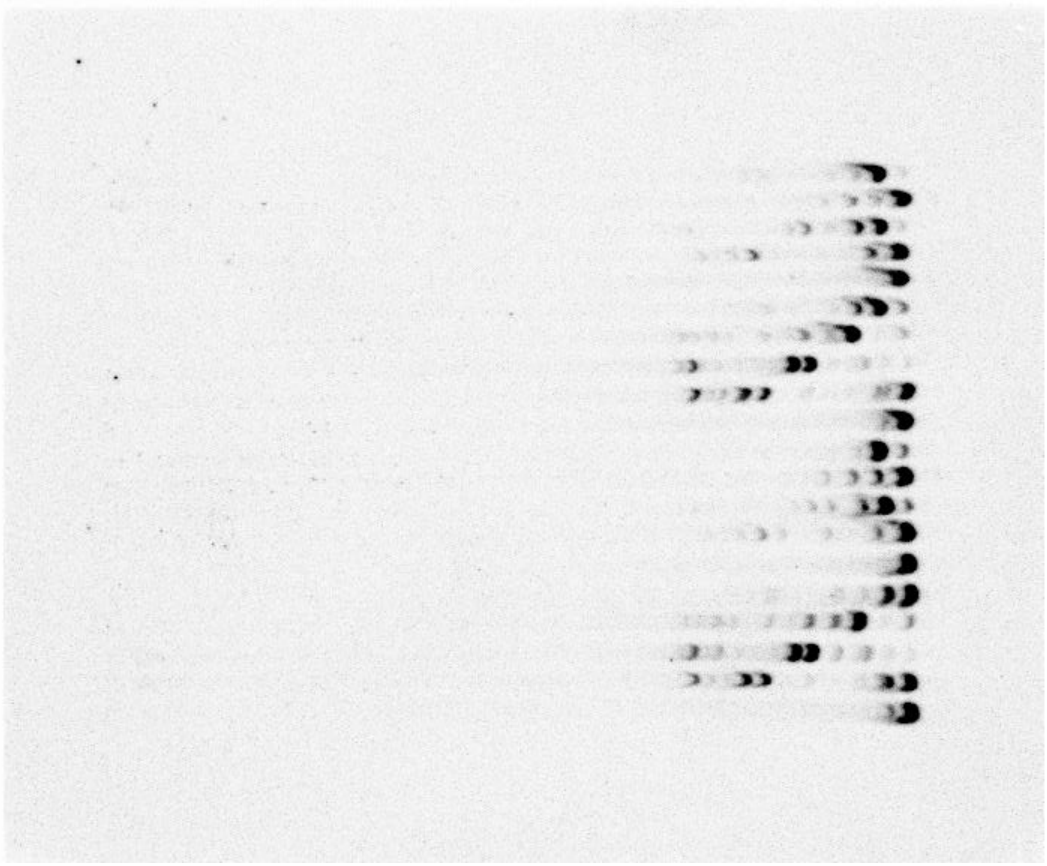

S11 File top

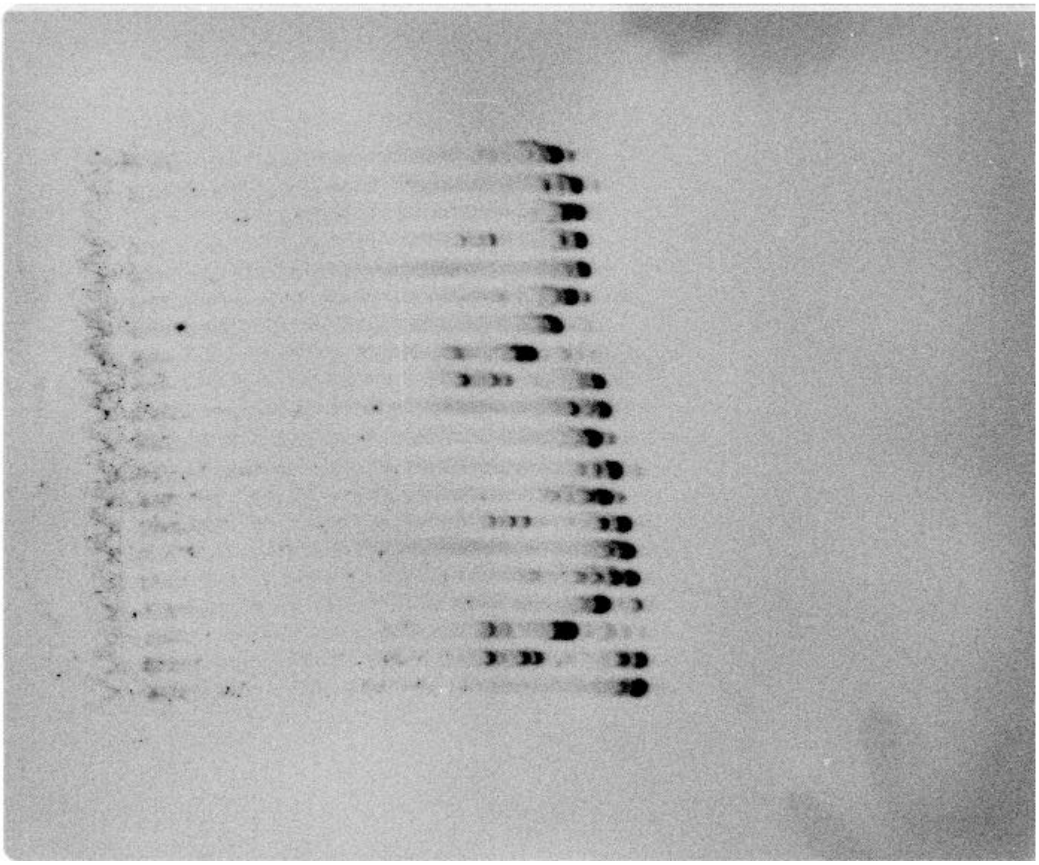

S11 Filebottom

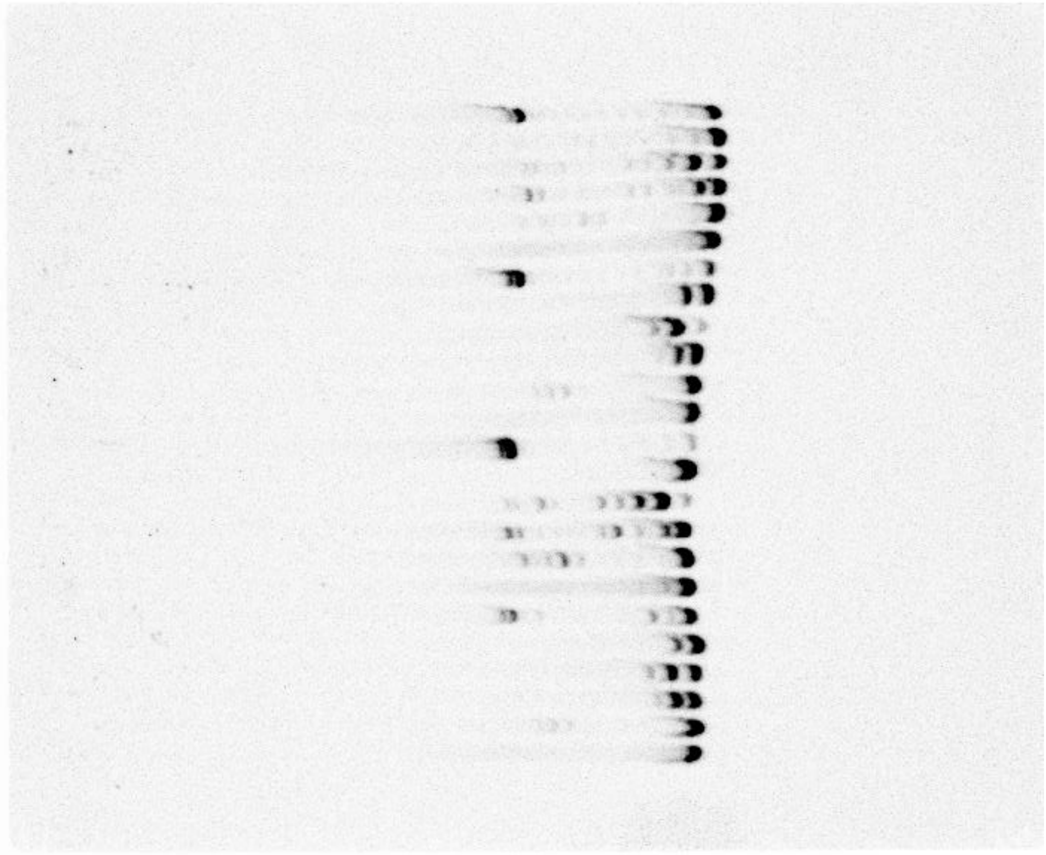

S11 File top – 2nd page

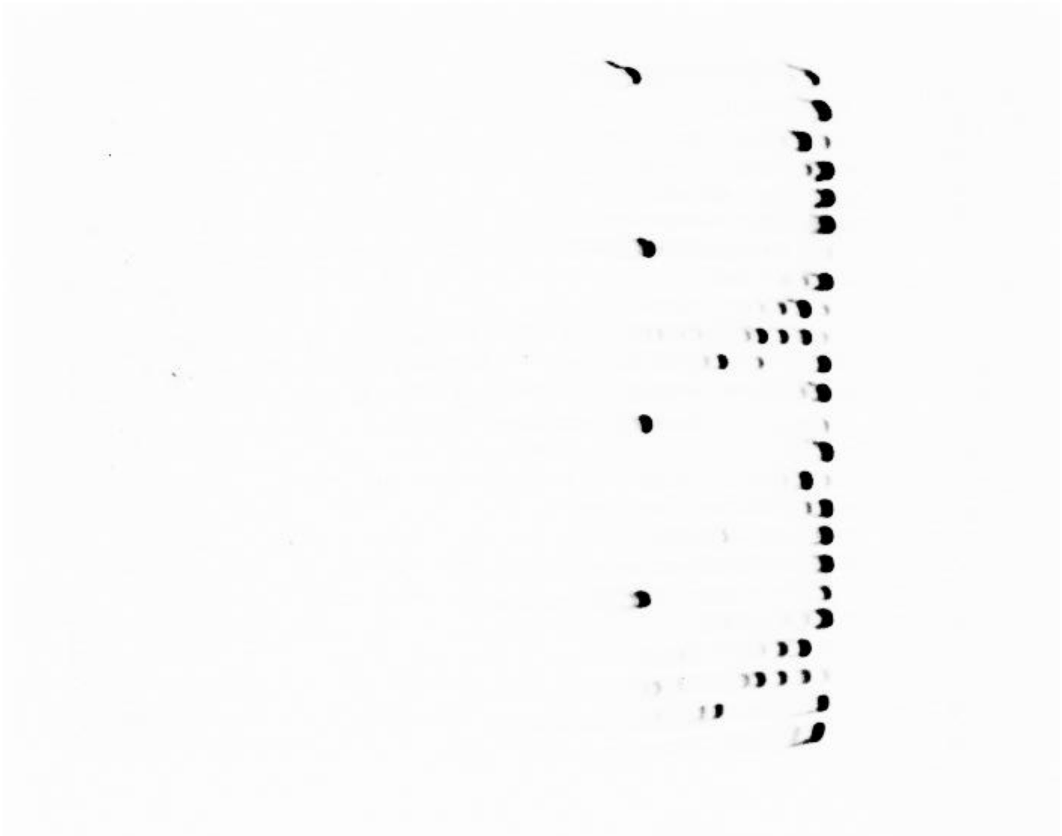

S11 File bottom – 2nd page

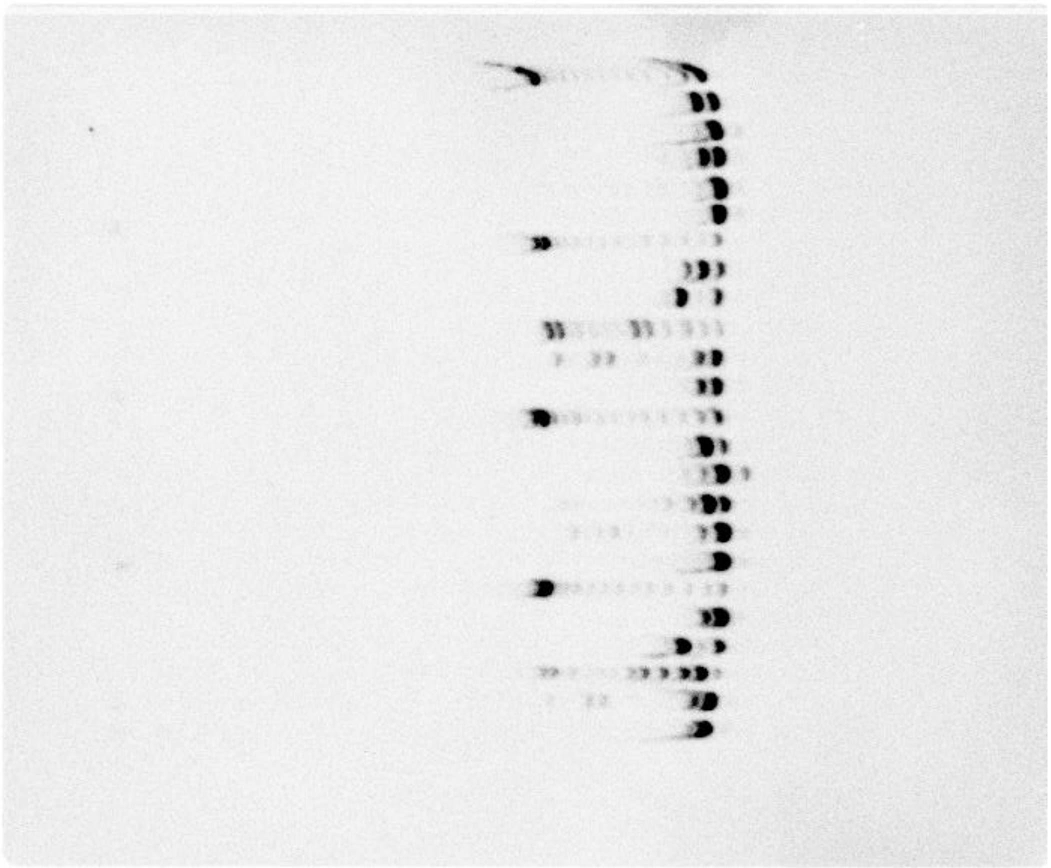

S12 File

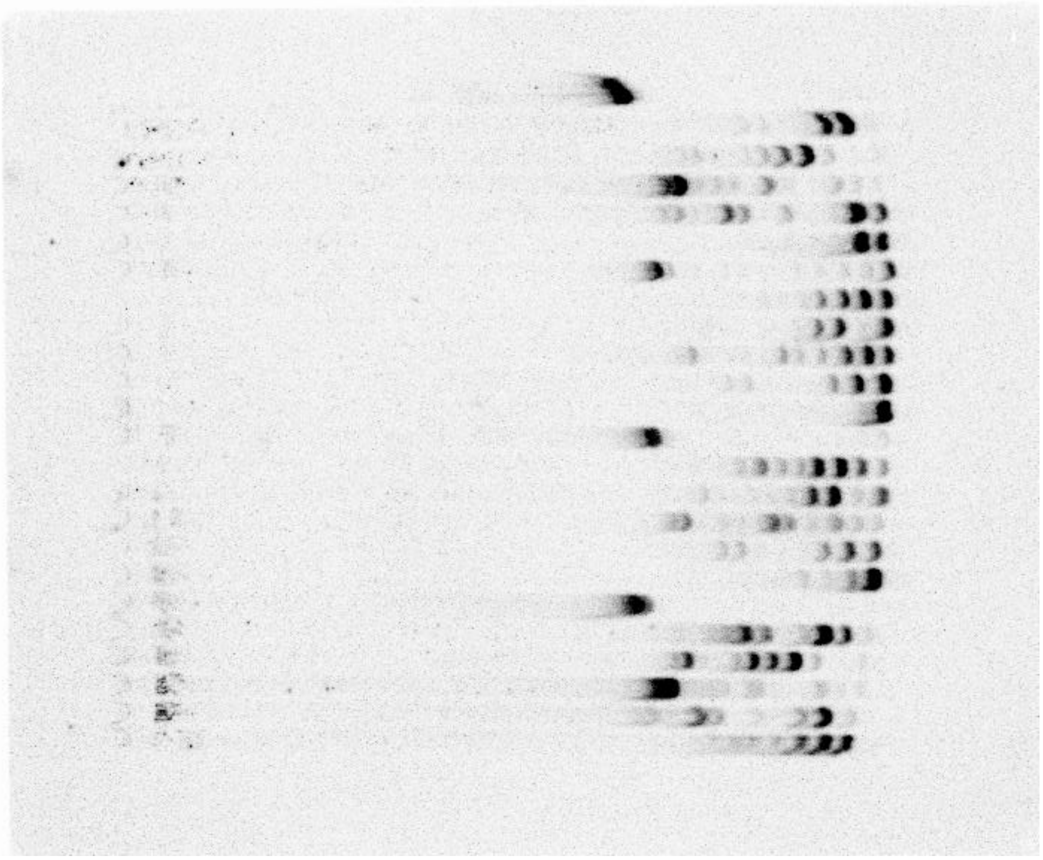

S13 File

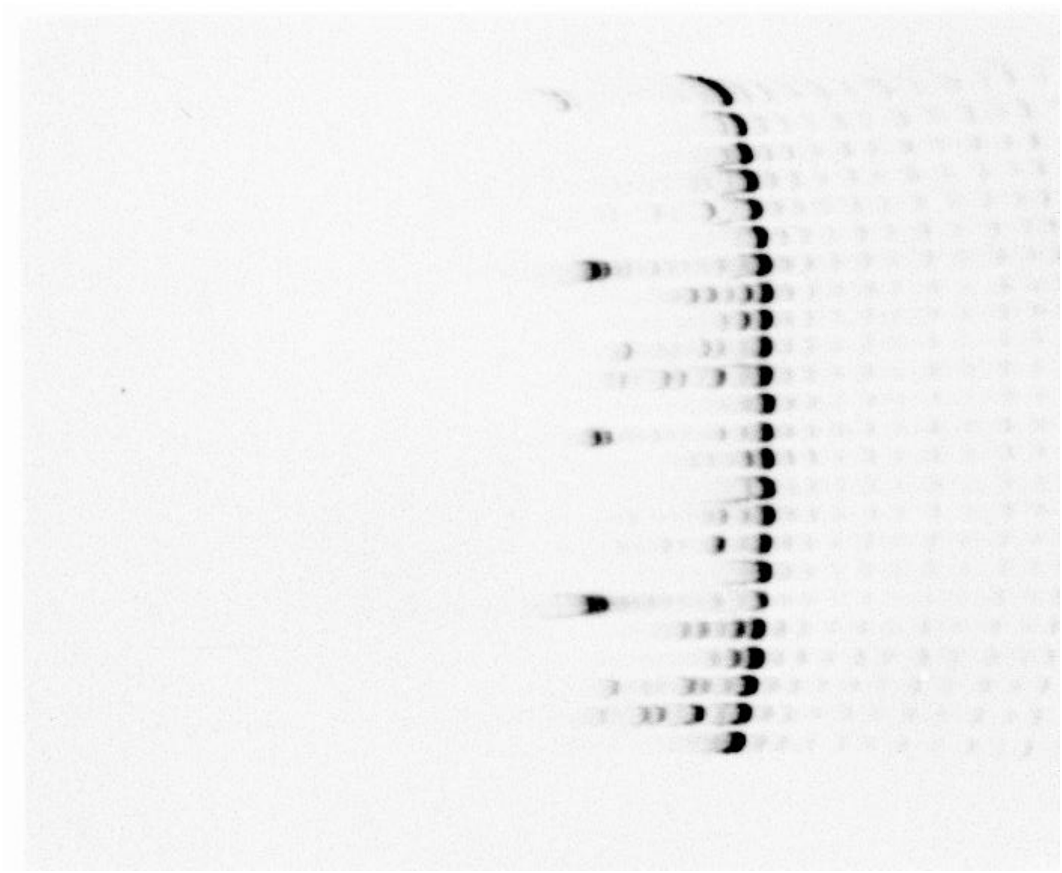

S14 File top

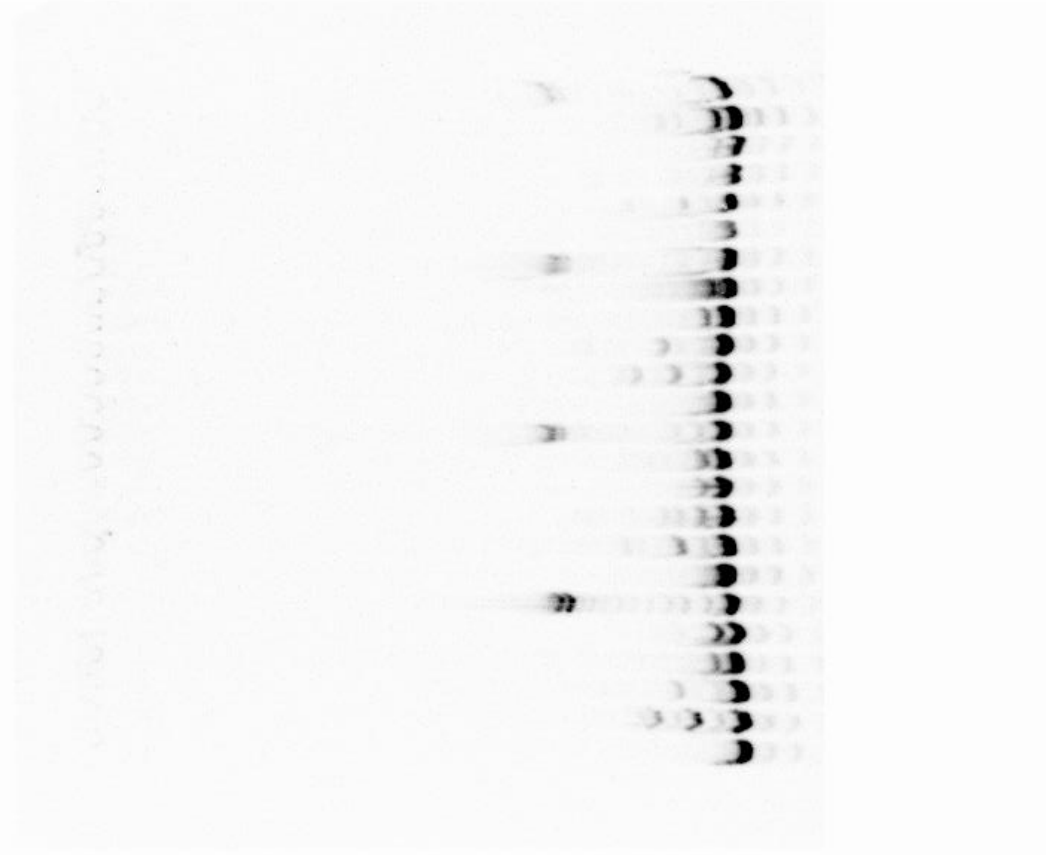

S14 File middle

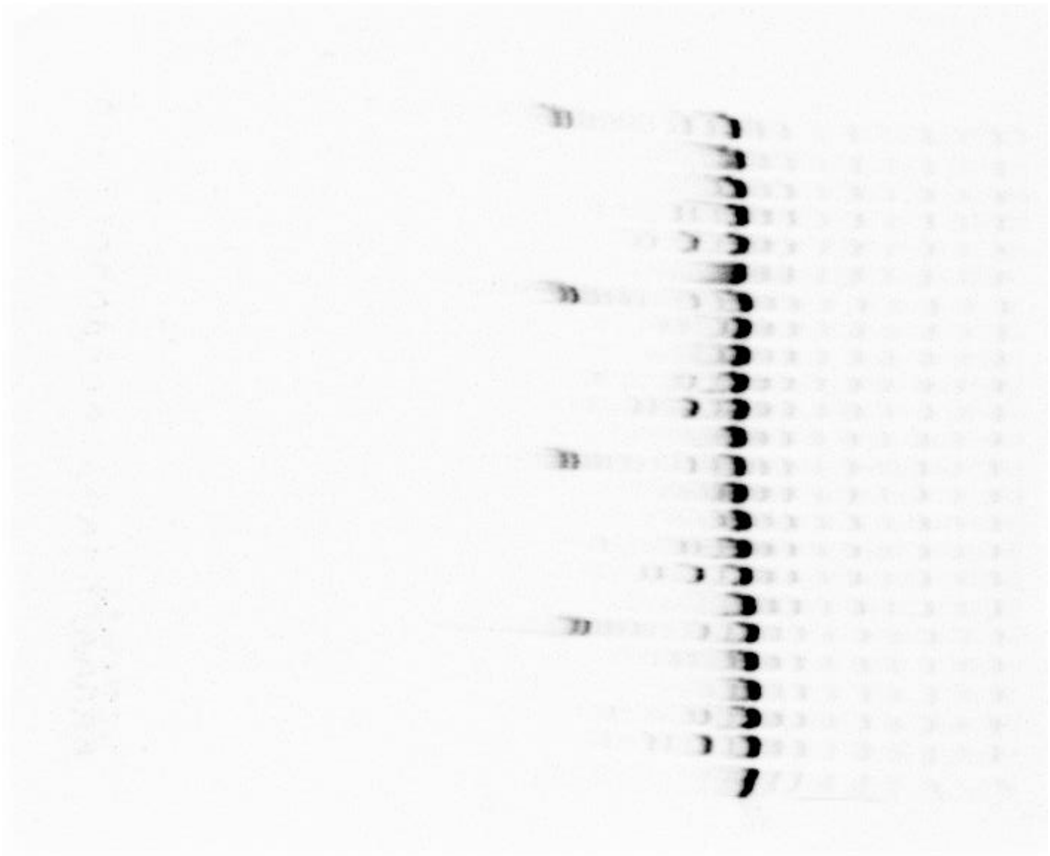

S14 File bottom

S15 File-A

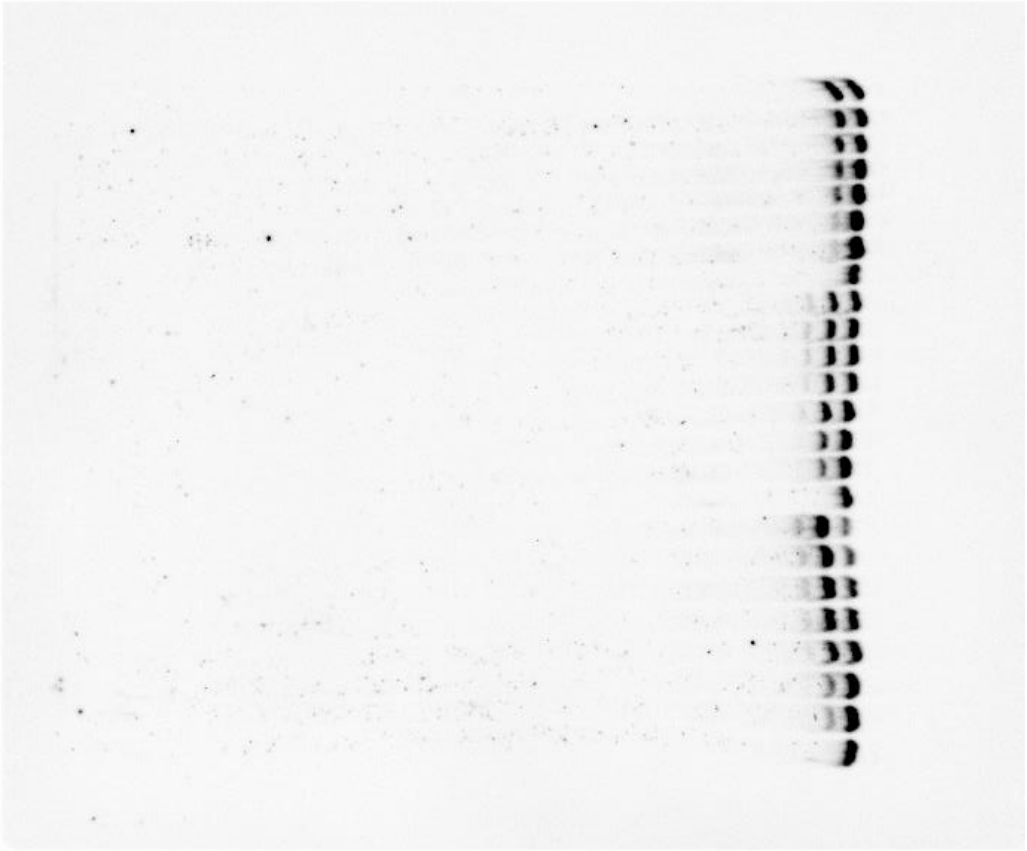

S15 File-A

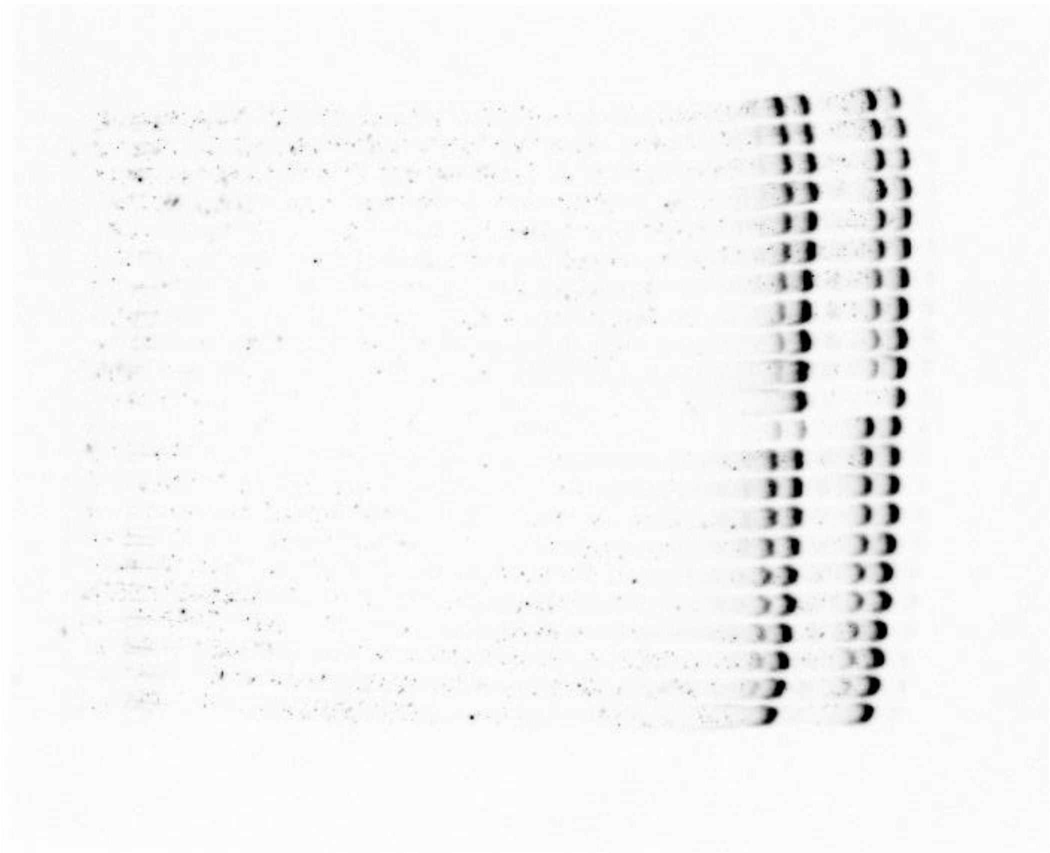

S15 FileB

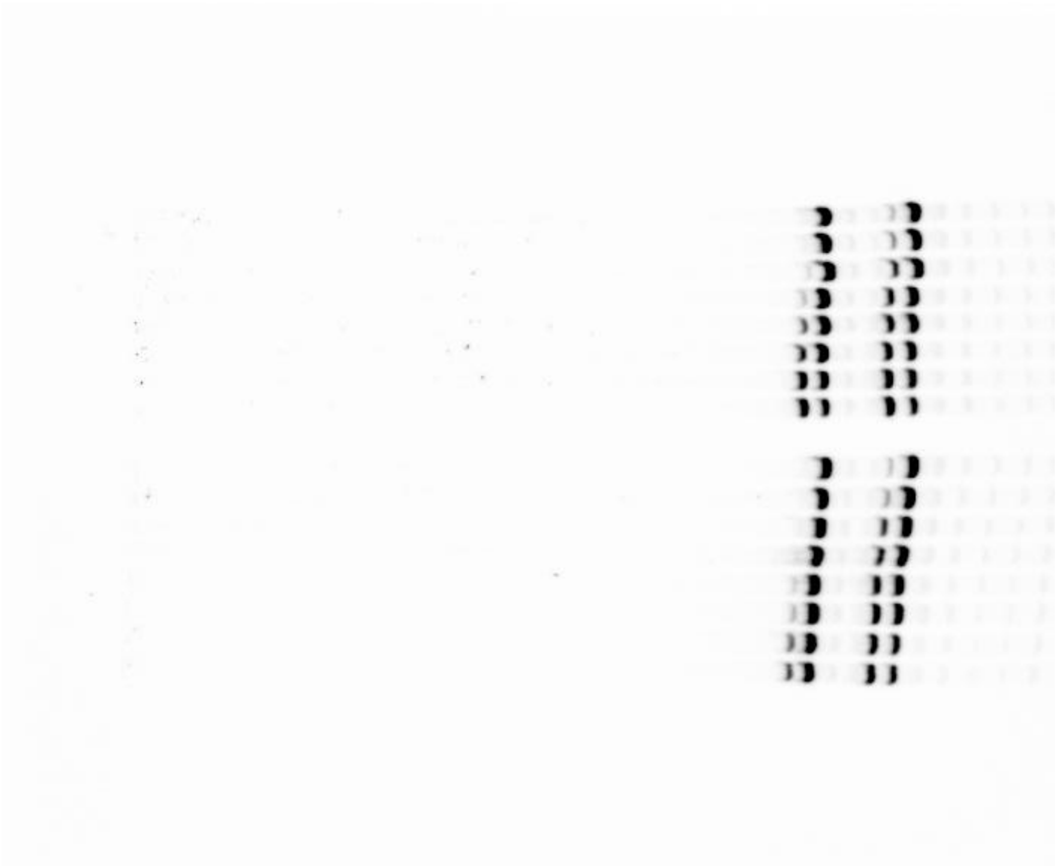

S15 File C
