# Supplementary material for: Translesion synthesis by AMV, HIV, and MMLVreverse transcriptases using RNA templates containing inosine, guanosine, and their 8-oxo-7,8-dihydropurine derivatives
Source: PLoS One. 2020 Aug 28;15(8):e0235102. doi: 10.1371/journal.pone.0235102 (PMC7455023; doi:10.1371/journal.pone.0235102)
Supplement: Attachment — Submitted filename: Response to Reviewers.pdf [file pone.0235102.s019.pdf]

COLLEGE OF LIBERAL ARTS AND SCIENCES
Department of Chemistry
UNIVERSITY OF COLORADO DENVER

Marino J. E. Resendiz
Associate Professor
University of Colorado Denver
Campus Box 194 P.O. Box 173364
Denver, CO 80217-3364
Phone: 303-315-7658
e-mail: marino.resendiz@ucdenver.edu

Re: PONE-D-20-17213

Monday, July 20, 2020

Dear Dr. Menéndez-Arias and members of the Editorial Board,

We appreciate the opportunity to re-submit a revised version of this manuscript for consideration as a Research Article. We appreciate the reviewer's comments and were able to address all of their comments/suggestions satisfactorily, as follow (response in blue for clarity):

Reviewer 1:

1. According to the title, readers may think this study mainly focus on the comparison of MMLV reverse transcriptase (RT), AMV RT, and HIV-1 RT. However, there is no data for MMLV RT in the main figures. I would like the authors to reconsider if the current title and the order of figures are appropriate.

We agree with this reviewer in that a lack of figures on the manuscript, using MMLV-RT, may be misleading with the title. The experiments were carried out and are included in the supporting information, we believe that leaving MMLV in the title is granted. We believe that the MMLV results are of some value and this data on the manuscript. In this regard, the order in which the RTs are mentioned in the title was changed. Also, to make this more clear, a few statements were added throughout the manuscript to remind the audience of these experiments, e.g., a statement was added at the end of the abstract: Similar results were obtained between AMV- and MMLV-RT.

2. I think tables in the figures should be put together into one or two tables.

As requested, the tables for the kinetic parameters were combined on Figure 3. We believe this helped in making this figure clearer. Figures containing a table for Tm values and kinetic parameters were left in its original format, we believe that leaving these two sets of data separately aid in dissecting the information.

3. Resolution of all figures should be increased.

As requested, new figures were uploaded.

4. Figure legends should be more informative. Enzyme species and concentration, pH, temperature, and reaction time are missing.

Although the overall procedure is described within the experimental information, we agree that it is easier to have that information more accessible. Each relevant figure legend (Figures 2-7) was edited to include this information.

5. Generally, the fidelity of reverse transcriptase depends on reaction condition. The error rate increased with increasing MgCl2 concentration. Please discuss possible effects of MgCl2 concentration on the results.

As also noted from Reviewer #2, the following was added:

-Added the following statement on the experimental section: ‘It is important to note that the Mg ion concentrations varied with each of the recommended conditions, and the amount of free Mg2+ was calculated as previously reported,41 to yield values of: 10, 312, or 371 µM for AMV and HIV RTs; 6, 12.2, or 15.8 µM for MMLV RT; and 0.45, 1, or 1.1 mM for SSII RT. Where values correspond to dNTP concentrations of 1.7, 0.5, or 0.425 mM respectively. Along with ref. 41(Achuthan, V.; Keith, B. J.; Connolly, B. A.; DeStefano, J. J. Human immunodeficiency virus reverse transcriptase displays dramatically higher fidelity under physiological magnesium condition in vitro. *J. Virol.* **2014**, 88, 8514-8527)

-The concentration of free Mg^{2+} was also added to all figure legends.

-The following statement was added in the discussion section:

Another important aspect to take into consideration regards to the relationship between concentration of divalent ions (in this case Magnesium) and RT fidelity, where Mg concentration can impact the outcome depending on the biological system, where lower fidelity occurs with HIV RT and not with MMLV RT;[60] or may alter the overall translation mechanism in other systems.[61] The work described herein was carried out in free Mg2+ concentrations that ranged from 10-370 µM range, which approaches physiological ranges of some biological systems (although the concentration of dNTP used is much higher than what would be encountered in vivo) and is on the lower end of concentrations typically used. Another aspect of consideration is the dNTP concentration used in this work, where increasing the concentration of dNTP led to transcription inhibition presumably due to a large amount of dNTPs

chelating to the divalent cation. Interestingly, dNTP concentrations up to 20 mM have been effectively used (while using the concentrations for all other species reported in this work) with other RNA modifications and the three reverse transcriptases used,[62] which suggests that the concentration of Mg at trace levels is sufficient to carry out the desired reactions in vitro.

1. TOC: At a first glance, each color represents dATP, dCTP, dGTP, or dTTP. If it is true, the color of primer is inadequate. Please change colors.

We agree with this reviewer and the figure was edited with a different color.

2. Page 5, line 15: “N” should be italicized. Fixed.

3. Page 10, line 6: I think “(2:5)” should be “(1:5)”. Fixed.

4. Page 11, line 6: I think “C:dT” should be “U:dC”. The original statement is correct, however the following parentheses were added for clarity: (such as in +3N-, and +4N-positions) and (such as at position +5N, where DNA synthesis stalled).

5. Please indicate what “WC” means. This has now been defined on its first appearance as: Watson-Crick (WC).

6. Experimental Procedures: The styles of subtitle are different including large capitals of all words or only the first word and italicized or not, which should be uniformed. The titles were made uniform. The only exception is where we opted to leave italicized sections under the ‘Reverse transcription experiments’ subtitle. In our opinion, this aids in separating the different type of experiments that were carried out under this section.

7. Figure 2: The difference between Fig. 2B and D is enzyme concentration (0.7 and 2.1 Units, respectively). Almost all substrate remained (lane 2 in Fig. 2B), while no substrate remained (lane 2 in Fig. 2D). Please discuss it.

The following statement was added: 2) that higher enzyme concentrations lead to lower fidelity on addition of dG (comparison between lane 2 and 22, where the latter displayed a more efficient addition);

8. Page 17, line 8 from the bottom: “dC” should be italicized. Fixed.

Reviewer #2

There were some concerns. The writing was not particularly clear. There were several grammatical errors, too many to list here. Therefore, a PDF document has been provided with several recommendations as comments.

We thank this reviewer for the careful assessment of the manuscript and for sending a separate pdf document. As suggested, all of the recommendations were adopted.

There is a general concern that the level of free Mg in reactions may have been very low. With MMLV-RT for example, it is not clear how the reactions worked with 0.3 mM Mg and levels of dNTPs that were above this value. Since dNTPs chelate Mg this would mean the free concentration of Mg in these reactions may have been near 0, essentially trace levels. This needs to be explained. Is it possible that this effected the outcome of some experiments? The reviewers need to reconsider their results after calculating how much free Mg was present. Finally, the fidelity of DNA replication by all the viruses that these RTs were derived from is similar in cells and more recent data indicates that it is also similar when physiological levels of Mg are used in vitro. There are clear differences in fidelity in vitro with high Mg and HIV is less accurate under this condition but is this is not the case with low Mg (see Achuthan et al. 2014, 15:8514). It appears that the results with HIV RT here were mixed and did not necessarily support it being less accurate than the other enzymes. The authors may want to take this into consideration.

We thank this reviewer for the input on this topic. Since this is an important point, the following changes/edits were made:

-Added the following statement on the experimental section: ‘It is important to note that the Mg ion concentrations varied with each of the recommended conditions, and the amount of free Mg2+ was calculated as previously reported,41 to yield values of: 10, 312, or 371 μM for AMV and HIV RTs; 6, 12.2, or 15.8 μM for MMLV RT; and 0.45, 1, or 1.1 mM for SSII RT. Where values correspond to dNTP concentrations of 1.7, 0.5, or 0.425 mM respectively. Along with ref. 41(Achuthan, V.; Keith, B. J.; Connolly, B. A.; DeStefano, J. J. Human immunodeficiency virus reverse transcriptase displays dramatically higher fidelity under physiological magnesium condition in vitro. *J. Virol.* **2014**, 88, 8514-8527)

-The concentration of free Mg^{2+} was also added to all figure legends.

-The following statement was added in the discussion section:

Another important aspect to take into consideration regards to the relationship between concentration of divalent ions (in this case Magnesium) and RT fidelity, where Mg concentration can impact the outcome depending on the biological system, where lower fidelity occurs with HIV RT and not with MMLV RT;[60] or may alter the overall translation mechanism in other systems.[61] The work described herein was carried out in free Mg2+ concentrations that ranged from 10-370 μM range, which approaches physiological ranges of some biological systems (although the concentration of dNTP used is much higher than what would be encountered in vivo) and is on the lower end of concentrations typically used. Another aspect of consideration is the dNTP concentration used in this work, where increasing the concentration of dNTP led to transcription inhibition presumably due to a large amount of dNTPs chelating to the divalent cation. Interestingly, dNTP concentrations up to 20 mM have been effectively used (while using the concentrations for all other species reported in this work) with other RNA modifications and the three reverse transcriptases used,[62] which suggests that the concentration of Mg at trace levels is sufficient to carry out the desired reactions in vitro.

As far as the outcomes with the HIV RT, the following was edited to read:
The ability of three reverse transcriptases to enable the synthesis of cDNA past I, 8-oxoG, 8-oxoI, or 8-BrI, was examined by focusing on position 18 of a 29-nt long RNA template. The use of AMV RT led to similar results to those obtained with MMLV RT, or HIV RT (slightly varying outcomes observed).
The following statement was added to the discussion:
The results obtained with duplexes 1:5 – 4:5 in the presence of HIV -RT were generally similar with differences that may be due to experimental conditions, e.g., was this was the only RT that enabled incorporation of dA opposite an RNA template containing G but less efficiently than the corresponding I-analogue

Reviewer #3.
1. What is the active site concentration of the enzyme preparations used in this study? This would affect the comparison of different reverse transcriptases, and Table 1 indicates that the polymerases were compared by unit concentration. As the unit assay and definition can vary between different enzymes and different companies, it seems appropriate to measure this for the study and compare the different reverse transcriptases appropriately.

We thank the reviewer for this suggestion, unfortunately we are not able to measure the active site concentration at this time. We agree that a comparison of the enzymes and more information about the amounts used is granted. As a consequence, we made the following edits:

Table 1 was edited and it now provides the concentration of enzyme provided by manufacturer, the amount of enzyme used per experiment, and the amount of enzyme used per experiment (as measured via UV-vis). The following is the label corresponding to Table 1:

- Concentration of reverse transcriptase used in this work. * These columns represent the number of units used per well (single experiment). † This value represents the concentration of the stock vial provided by manufacturer (different batches of enzyme varied slightly). § This value represents the number of grams used for both low and high concentrations used in this work, per well/experiment. ‡ This value was obtained from UV-vis measurements of each RT using reported extinction coefficients (ε values for HIV RT is known42 while AMV and MMLV values have not been reported). Unit definition: HIV -One Unit incorporates 1 nmole of tritiated d-TMP into acid precipitable products using poly(A)/oligo(dT)12-18 as the template/primer in 20 minutes at 37°C, pH 8.3; MMLV or AMV - One unit incorporates 1 nmol of dTTP into acid-insoluble material in a total reaction volume of 50 µl in 10 minutes at 37°C using poly(rA).oligo(dT) as template primer with 50 mM Tris-HCI (pH 8.3), 6 mM $MgCl_2$, 10 mM dithiothreitol, 0.5 mM [3H]-dTTP and 0.4 mM poly(rA).oligo(dT)12-18.
- UV-vis spectra of the three enzymes was recorded and added to the supporting information (Figure S10).

We believe that the mentioned edits are sufficient and that the overall conclusions do not change, while giving the reader a sense of comparison between different reverse transcriptases.

2. Regarding the pH dependence of nucleotide incorporation opposite deoxyinosine: the authors did not address the stability of the RNA template over the pH range tested.

We thank this reviewer for the comment. A statement explaining the stability of the RNA over this pH range was added: Importantly, no bands corresponding to degradation/cleavage products were observed upon subjecting the oligonucleotides to this pH range under the described experimental conditions.

In addition, a statement supporting the trend dependability with respect to pH was added (along with the corresponding reference: Sci. Rep. 2018, 8:627).

Journal requirements:

1. All the formatting of PLOS One was applied to the manuscript.

2. The reporting of the kinetic parameters were adjusted according to STRENDA guidelines. In this regard, the following edits were made:

- K_{cat} values were added to the pertinent tables.

- a more detailed explanation on how everything was calculated is now provided in the experimental section: Reaction velocities were measured at various substrate concentrations and the values for V_{max} and K_m were obtained by plotting [S]/V vs [S] to obtain a linear graphical representation where: slope = $1/V_{max}$; K_m = y-intercept•V_{max}; K_{cat} was calculated by dividing V_{max} over 0.7 (units used per experiment). . Excell was used in all calculations. Different enzyme batches, from same manufacturer, were used throughout.

3. The original files for all gels have been included within the supporting information.

4. The table has been included within the text of the manuscript.

Protocol for reverse transcription was added on the Materials and methods section:

The protocol for the reverse transcription experiments is available at: dx.doi.org/10.17504/protocols.io.birskd6e

The following paragraph comes from the original cover letter. As none of the conclusions were changed, although we believe the manuscript is much stronger after following the reviewer's suggestions, the original cover letter is the same:

This manuscript explores the impact that inosine, 8-oxo-7,8-dihydroguanosine, and 8-oxo-7,8-dihydroinosine have on reverse transcription (RTn). Where interesting/unprecedented reactivity with RNA templates containing these modifications/lesions is presented. Primers of different size were employed to explore the implications that the modifications have as a function of position within the vicinity of transcription. The findings herein are novel since this process has not been explored with these modifications in a position dependent manner, and using templates longer than one or two nucleotides long past the modification. There is one report by the Burrows' group (ref. 38) where RTn was explored using 8-oxoG within the template strand, however the impact of this modification was explored at one position and with templates that only allowed for the addition of one or two deoxynucleotides. To obtain the modified strands of RNA, the synthesis corresponding to the

phosphoramidites of 8-oxoI and 8-BrI was developed and is reported herein for the first time. The modifications under study are of interest given that oxidation of RNA has been a topic that is gaining relevance and attention from researchers worldwide; and that inosine is ubiquitous and potentially related to viral processes. Our findings and data are of appeal to a broad audience expanding on fields such as nucleic acid chemistry, bioorganic chemistry, organic chemistry, biochemistry, structural biology and biophysics amongst others.

Please do not hesitate to contact me should you need additional information.

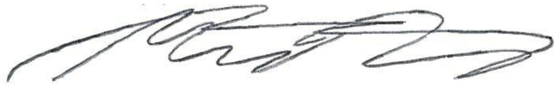

Prof. Marino J. E. Resendiz
